# Age-period-cohort analysis of autism spectrum disorders-related prevalence and DALYs: based on the Global Burden Of Disease Study 2021

**DOI:** 10.3389/fpsyt.2025.1570276

**Published:** 2025-04-25

**Authors:** Dong Li, Zhide Liang, Chuyuan Miao, Lan Li, Chenmu Li

**Affiliations:** ^1^ School of Physical Education and Health, Zhaoqing University, Zhaoqing, China; ^2^ School of Health Sciences and Sports, Macau Polytechnic University, Macau, China; ^3^ Day Surgery Centre, Shenzhen Nanshan People's Hospital, Shenzhen, China; ^4^ School of Social Sciences, Vitebsk State University, Vitebsk, Belarus; ^5^ School of Physical Education, Guangzhou Sport University, Guangzhou, China

**Keywords:** autism spectrum disorder, developmental disorder, global burden, incidence, health policy

## Abstract

**Background:**

The Sustainable Development Goals (SDGs) call for systematic monitoring to optimize child development outcomes. As a developmental disorder affecting children and adults, Autism Spectrum Disorder (ASD) not only impacts individual social functioning but also places a burden on families and society. A detailed analysis of the latest global burden data on ASD can assist stakeholders in formulating support policies and interventions, thereby helping to meet the health needs of ASD.

**Methods:**

We used data from the Global Burden of Disease Study 2021 (GBD 2021), compiled by the Institute for Health Metrics and Evaluation (IHME). Data were obtained through the Global Health Data Exchange (GHDx) and covered 204 countries and territories from 1990 to 2019. Variables included ASD-related prevalence, mortality, disability-adjusted life years (DALYs), age-standardized rates, and the sociodemographic index (SDI).

**Results:**

Exposure to autism spectrum disorders contributed to 61823540 prevalence and 11544038 DALYs globally in 2021. Males and younger adults were high-risk populations. Higher socio-demographic index (SDI) regions were high-risk areas. The disease burden varied considerably across the GBD regions and the countries. From 1990 to 2021, the number of cases increased. The predicted results showed that the disease burden for both genders would still increase from 2022 to 2046. Countries or regions with a higher SDI have greater burden improvement potential.

**Conclusion:**

The global burden of ASD has shown a continuous upward trend, with some differences observed across gender, age groups, and SDI regions. In terms of gender, the burden of ASD among females may be underestimated. Regarding age groups, the aging process has highlighted the urgent need to address ASD in the elderly population. High-SDI regions should place greater emphasis on improving diagnostic methods and implementing precise interventions, while middle- and low-SDI regions should focus on raising public awareness and enhancing screening capabilities.

## 1 Introduction

Autism Spectrum Disorder (ASD) is a complex developmental disorder characterized by difficulties in social interaction, restricted interests, and repetitive behaviors (1, 2). It typically manifests in early childhood and can have lifelong effects on an individual’s social functioning, including reduced opportunities for education, employment, and social inclusion (3). Additionally, individuals with ASD often experience comorbid conditions such as anxiety, depression, and attention-deficit hyperactivity disorder (ADHD) (4), which further exacerbate the decline in their quality of life and difficulties in social integration (5, 6). However, the specific causes and mechanisms underlying ASD remain unclear, potentially involving complex interactions between genetic, environmental, and social factors (7). With the global prevalence of ASD continuing to rise, particularly in high-income countries, the disorder has become a significant challenge in the field of global public health (8–10). Therefore, a comprehensive understanding of the prevalence and disease burden of ASD across different regions and populations is crucial for developing effective public health policies and interventions.

Recent studies have explored the use of machine learning models for the early prediction of autism in children, highlighting the potential of computational approaches in improving ASD diagnosis and monitoring (11). The Global Burden of Disease (GBD) study is a global health research project that aggregates the burden of various diseases, injuries, and risk factors across different regions and populations (12). Data for the GBD study come from multiple sources, including hospital records, disease surveillance systems, community surveys, health service usage, and demographic data (13). The GBD study also provides estimates of the prevalence, incidence, mortality, and disability-adjusted life years (DALYs) for ASD, offering valuable data to researchers for in-depth analysis of ASD’s global and regional impacts (14). The development of the Global Burden of Disease (GBD) database has advanced researchers’ ability to identify disease-specific patterns and has provided objective and reliable evidence for future studies (15).

Disability-adjusted life years (DALYs) are a comprehensive metric for measuring the burden of disease, proposed and widely adopted by the World Health Organization (WHO) and the GBD study. DALYs consist of two components: years of life lost due to premature mortality (YLL) and years lived with disability (YLD), and are used to assess the overall health loss caused by different diseases in populations (16). This indicator reflects both the fatal and non-fatal burden of diseases and serves as an important tool internationally for informing public health policies and setting intervention priorities (17).

In the GBD study, DALYs have been widely applied in health assessments and disease trend monitoring across numerous countries and regions, particularly in evaluating the societal burden of long-term health conditions such as chronic diseases, mental disorders, and developmental disorders (17). Therefore, incorporating DALYs into the burden assessment of ASD is beneficial for understanding the impact of this condition on public health systems from a macro-level perspective. However, there is still a lack of up-to-date information on the spatial distribution, temporal trends, and future projections of ASD burdens at the global, regional, and national levels, particularly concerning the differentiated impacts across regions with varying levels of social demographic development (SDI). This is crucial for formulating targeted public policies and reducing public health expenditures in regions with different development levels.

To better understand the temporal trends in the incidence and mortality of ASD, the Age-Period-Cohort (APC) model has been widely applied in epidemiological and public health research. This model separates the effects of three key time-related factors—age effects, period effects, and cohort effects—to help researchers identify changes in risk across different age groups, the influence of external events or environmental changes during specific historical periods, and the unique health risks faced by individuals born in different time cohorts (18, 19). Due to the strong linear dependency among the three factors (Cohort = Period – Age), traditional statistical methods struggle to simultaneously identify their independent effects. To address this “identifiability problem,” recent studies have developed various approaches such as constraint-based methods, decomposition techniques, and Bayesian models (20). In disease surveillance and forecasting, the APC model provides reliable evidence-based support for policymakers and has proven particularly valuable in the fields of mental disorders, chronic diseases, and developmental disorders in children, such as ASD.

To more accurately predict the future burden of ASD, this study aims to leverage data from GBD 2021 to conduct a comprehensive analysis of ASD prevalence and DALYs at global, regional, and national levels from 1990 to 2021. By exploring temporal trends and regional disparities in ASD burden, the study will identify key factors driving these trends. Additionally, the APC model will be employed to forecast changes in ASD burden over the next 25 years. Cutting-edge analytical methods will be utilized to assess the relationship between disease burden and social demographic development, revealing potential opportunities for health improvement across regions with varying development levels. These findings will help visualize global and regional epidemiological trends and burden changes of ASD, providing critical reference points for the development of targeted intervention strategies and resource allocation plans. ASD Differences in disease burden across regions may be associated with variations in access to healthcare resources, diagnostic capacity, and public awareness. Li et al. (2019) found that regions with higher socioeconomic status and more developed healthcare systems tend to report higher prevalence rates of ASD, which may be closely related to improved diagnostic capabilities and increased public awareness (10). Therefore, when evaluating the global burden of ASD, it is essential to consider contextual factors such as regional levels of social development and healthcare system capacity.

Although several studies have utilized the GBD database to analyze the burden of ASD, most have focused on descriptive analyses within the 1990–2019 period, primarily addressing temporal trends or regional disparities, without employing predictive models or advanced statistical approaches (21). Solmi et al. (2022) analyzed global and regional ASD burden trends using GBD 2019 data but did not incorporate advanced methods such as the APC model (21). Similarly, the study by Li et al. (2022) provided insights into the distribution of ASD burden in China and globally but did not project future disease burden or explore its associations with sociodemographic factors (22). Therefore, this study integrates GBD 2021 data with APC modeling, ARIMA forecasting, exponential smoothing, and frontier analysis to conduct a comprehensive temporal assessment and prediction of the ASD burden. This multi-method, advanced analytical approach offers a deeper understanding of historical changes and future trends in ASD burden and provides novel evidence to inform public health policies at both global and regional levels (23).

The GBD study was initially launched by the World Health Organization and is currently led by the Institute for Health Metrics and Evaluation (IHME) at the University of Washington. It is one of the most authoritative and comprehensive global health data projects to date. Since its inception in the 1990s, the GBD has evolved into a standardized assessment framework covering 204 countries and territories and encompassing 369 diseases and injuries. It provides estimates of incidence, prevalence, mortality, and DALYs, stratified by age, sex, region, and year (24). In recent years, GBD data have been widely applied in burden assessments of neurodevelopmental and mental disorders, including ASD (17, 22). Existing studies have explored temporal trends, regional disparities, and associations with sociodemographic factors; however, few have incorporated predictive models or in-depth decomposition methods. This study, based on the latest GBD 2021 data, aims to comprehensively evaluate and forecast the burden of ASD across regions by integrating APC analysis, ARIMA forecasting, exponential smoothing, and frontier efficiency analysis, thereby addressing existing research gaps.

## 2 Methods

### 2.1 Data sources

The present study is based on the GBD 2021, which represents the most up-to-date and comprehensive dataset currently available for estimating global and regional disease burden (25). Compared to previous GBD iterations (e.g., GBD 2019), GBD 2021 incorporates updated data sources, revised modeling strategies, and extended time series, thereby providing a more accurate and contemporary picture of ASD-related disability and mortality. To ensure the rigor and reliability of the statistical analyses applied in this study, including the APC model, ARIMA forecasting, exponential smoothing, and frontier efficiency analysis, all methods were reviewed and validated by an experienced statistician.

The primary data sources for this study were the Global Health Data Exchange (GHDx) and the IHME database (26, 27). These databases compile comprehensive information on autism spectrum disorders prevalence and DALYs from various countries and regions globally. We accessed the most recent estimates of autism spectrum disorders prevalence and DALYs, spanning from 1990 to 2021.

To ensure the robustness of our analyses, the GBD 2021 dataset already incorporates standardized protocols for dealing with missing or incomplete data through advanced statistical modeling. In this study, we adhered to the default data preprocessing methods provided by the Institute for IHME. Additionally, for country-year-age groups with missing estimates, the GBD database provides modeled estimates with corresponding 95% uncertainty intervals (UIs), which were used directly in our analyses to maintain data consistency and comparability. No further manual imputation was conducted, and sensitivity analyses using ARIMA and ES models were applied to validate the stability of the results derived from modeled inputs.

For the purpose of geographical analysis, the global encompassing 204 countries was systematically partitioned into 54 distinct GBD regions (27). Additionally, these 204 countries and territories were stratified into five SDI tiers: low, low-middle, middle, high-middle, and high, providing a nuanced understanding of socioeconomic disparities (28). The methodological underpinnings of the GBD 2021 framework have been thoroughly detailed in preceding publications (29, 30), ensuring reproducibility and transparency. Data acquisition was multifaceted, drawing upon an array of reputable sources such as cancer registries, peer-reviewed studies, surveillance systems, census records, and other pertinent databases (31). Each dataset was accompanied by its corresponding 95% uncertainty intervals (UIs), facilitating robust error propagation and assessment of the estimates’ precision. To quantify the overall burden of disease, DALYs were computed by the aggregation of Years of Life Lost (YLLs) due to premature mortality and Years Lived with Disability (YLDs) attributed to each individual cause of health loss (32).

### 2.2 Statistical analyses

First, the prevalence and causes of DALYs attributable to autism spectrum disorders, along with their Age-Standardized Rates (ASRs), were assessed for 2021 on a global scale, further stratified by sex, age, SDI regions, GBD regions, and countries. Next, a linear regression model was used to calculate the Estimated Annual Percentage Change (EAPC) values and to evaluate the temporal trends in the global burden from 1990 to 2021. Additionally, the association between the EAPCs in 1990 and the ASRs related to autism spectrum disorders, as well as the Human Development Index (HDI) values in 2021, was assessed. Given that these three factors do not follow a normal distribution, Spearman correlation analysis was employed to explore the associations among them. Furthermore, hierarchical clustering analysis was conducted using EAPC values, categorizing the 54 GBD regions into four distinct groups: significantly increasing, slightly increasing, stable or slightly decreasing, and significantly decreasing, to identify evolving patterns of disease burden in different GBD regions. To project the future disease burden from 2022 to 2046, an APC model integrated with nested Laplace approximations was employed. Sensitivity analyses were performed using the Autoregressive Integrated Moving Average (ARIMA) model and Exponential Smoothing (ES) model. These two time-series forecasting models are widely used in the field of epidemiology, as they can reveal data trends from different perspectives, complement each other, and enhance the scientific rigor and credibility of the projections. The ARIMA model is well-suited for handling non-stationary data by capturing linear temporal dependencies through autoregressive, differencing, and moving average components. It has been extensively applied in disease trend forecasting, including studies on mental disorders and developmental conditions (33). ES model is more suitable for forecasting data with relatively stable trends or short-term fluctuations, making it particularly useful for rapid response needs in public health management (34). In this study, the use of both ARIMA and ES models not only validated the stability of the APC model’s predictions but also provided a more comprehensive perspective for evaluating future trends in the burden of autism.

Finally, frontier analysis was used to assess the association between disease burden and socioeconomic development. A nonlinear frontier was established using a nonparametric Data Envelopment Analysis (DEA) method to determine the minimum achievable burden based on the current development levels of countries or regions. The efficiency gap represents the difference between observed ASRs and the frontier, indicating the potential for health improvements under current development conditions.

If the P-value was under 0.05, it was deemed statistically significant. R software (version 4.0.2) was employed for database creation, organization, and analysis.

## 3 Results

### 3.1 The disease burden attributable to autism spectrum disorders in 2021

In 2021, the number of autism spectrum disorders-related prevalence was 61823540 [95% uncertainty intervals (UI): 52067673-72711238], with the corresponding age-standardized prevalence rate (ASPR) was 788.34 (95% UI: 663.76-927.21) per 100,000 population. The number of DALYs cases was 11544038 (95% UI: 7842315-16288865), and the corresponding ASR of DALYs was 147.56 (95% UI: 100.21-208.16) per 100,000 population (**Tables 1**, **2**).

**Table 1:** The number of prevalence cases and the age-standardized prevalence rate attributable to autism spectrum disorders in 1990 and 2021, and its trends from 1990 to 2021 globally.

	1990		2021		
Characteristics	Number of prevalence cases (95% UI)	The age-standardized prevalence rate/100000 (95% UI)	Number of prevalence cases (95% UI)	The age-standardized prevalence rate/100000 (95% UI)	EAPC (95% CI)
**Global**	41929996 (35349448-49633546)	773.25 (651.35-914.71)	61823540 (52067673-72711238)	788.34 (663.76-927.21)	0.07 (0.07-0.08)
Sex					
Female	13349055 (11142957-15864043)	497.32 (414.72-590.83)	19689661 (16462872-23416673)	508.08 (424.62-604.33)	0.09 (0.08-0.1)
Male	28580940 (24105174-33702935)	1046.01 (883.1-1233.08)	42133879 (35549495-49294565)	1064.71 (898.47-1245.7)	0.06 (0.06-0.07)
Age					
<5 years	5280984 (4431768-6250170)	851.86 (714.87-1008.19)	5776990 (4866465-6810367)	877.73 (739.39-1034.74)	0.1 (0.09-0.11)
5-9 years	4848675 (4073795-5723075)	830.92 (698.13-980.77)	5861519 (4942505-6894127)	853.14 (719.38-1003.43)	0.12 (0.1-0.13)
10-14 years	4388638 (3698027-5184030)	819.26 (690.34-967.74)	5606055 (4728896-6581795)	840.95 (709.37-987.31)	0.11 (0.1-0.12)
15-19 years	4182362 (3520103-4946274)	805.19 (677.69-952.26)	5185365 (4372351-6086212)	831.01 (700.72-975.39)	0.09 (0.08-0.11)
20-24 years	3910409 (3291729-4620444)	794.66 (668.93-938.95)	4899809 (4131439-5745809)	820.52 (691.85-962.19)	0.08 (0.06-0.1)
25-29 years	3533582 (2972689-4175288)	798.33 (671.61-943.31)	4768910 (4011192-5592515)	810.57 (681.78-950.55)	0.07 (0.05-0.09)
30-34 years	3098310 (2604270-3668426)	803.87 (675.69-951.79)	4823728 (4053340-5662970)	798 (670.55-936.83)	0.02 (0-0.05)
35-39 years	2790471 (2345232-3313765)	792.2 (665.8-940.76)	4453162 (3746269-5242198)	793.98 (667.94-934.66)	0.01 (-0.01-0.04)
40-44 years	2281286 (1916659-2704528)	796.31 (669.03-944.05)	3944563 (3324042-4657038)	788.52 (664.48-930.94)	-0.04 (-0.06–0.01)
45-49 years	1812036 (1520596-2146319)	780.39 (654.88-924.36)	3650661 (3076103-4309607)	770.99 (649.65-910.15)	-0.06 (-0.08–0.04)
50-54 years	1621506 (1360606-1915817)	762.81 (640.07-901.26)	3345041 (2810907-3947679)	751.82 (631.77-887.27)	-0.06 (-0.09–0.04)
55-59 years	1366689 (1143771-1612549)	737.95 (617.59-870.71)	2900842 (2425070-3421920)	733.04 (612.81-864.72)	0 (-0.02-0.03)
60-64 years	1134869 (939784-1334065)	706.6 (585.14-830.63)	2301053 (1910658-2709002)	718.97 (596.99-846.44)	0.06 (0.04-0.08)
65-69 years	785444 (647676-923967)	635.42 (523.97-747.49)	1826905 (1506791-2156439)	662.3 (546.25-781.77)	0.16 (0.14-0.18)
70-74 years	458047 (376292-542385)	541.04 (444.47-640.65)	1240193 (1019197-1471770)	602.51 (495.14-715.01)	0.34 (0.32-0.36)
75-79 years	272449 (220327-327825)	442.61 (357.93-532.57)	671616 (544299-807970)	509.25 (412.71-612.63)	0.51 (0.48-0.55)
80-84 years	119327 (93004-148243)	337.31 (262.9-419.05)	365059 (287977-448612)	416.81 (328.8-512.21)	0.74 (0.7-0.77)
85-89 years	36650 (26845-48053)	242.54 (177.65-318)	149107 (112289-192249)	326.12 (245.59-420.48)	1.03 (1-1.06)
90-94 years	7154 (4875-10165)	166.94 (113.76-237.21)	43369 (30483-59005)	242.43 (170.4-329.83)	1.28 (1.26-1.3)
95+ years	1107 (670-1682)	108.69 (65.77-165.18)	9592 (6113-14067)	175.99 (112.15-258.09)	1.54 (1.53-1.56)
SDI region					
High-middle SDI	8301430 (6971718-9847647)	777.19 (652.71-922.06)	10045486 (8419995-11895394)	796.49 (668.01-941.36)	0.11 (0.1-0.12)
High SDI	8972557 (7567751-10578501)	1038.81 (875.46-1223.08)	11056986 (9259242-13087468)	1057 (887.17-1248.84)	0.06 (0.05-0.08)
Low-middle SDI	8496267 (7125138-10072685)	698.66 (585.96-828.18)	14051075 (11803670-16488637)	716.95 (601.88-841.51)	0.08 (0.07-0.08)
Low SDI	4210210 (3540870-4983755)	789.11 (663.27-936.77)	9543180 (8011930-11232297)	809.1 (679.47-951.25)	0.08 (0.07-0.08)
Middle SDI	11909533 (10029100-14130928)	669.36 (563.76-793.44)	17078128 (14382176-20164787)	703.96 (592.93-831.21)	0.17 (0.16-0.18)

UI, uncertainty interval; CI, Confidence Interval; EAPC, Estimated Annual Percentage Change; ASD, autism spectrum disorder; SDI, socio-demographic index.

**Table 2:** The number of DALYs cases and the age-standardized DALYs rate attributable to autism spectrum disorders in 1990 and 2021, and its trends from 1990 to 2021 globally.

	1990		2021		
Characteristics	Number of DALYs cases (95% UI)	The age-standardized DALYs rate/100000 (95% UI)	Number of DALYs cases (95% UI)	The age-standardized DALYs rate/100000 (95% UI)	EAPC (95% CI)
**Global**	7868387 (5351164-11069617)	144.51 (98.3-203.28)	11544038 (7842315-16288865)	147.56 (100.21-208.16)	0.08 (0.08-0.09)
Sex					
Female	2488810 (1714941-3506510)	92.39 (63.7-130.09)	3647200 (2492450-5139412)	94.45 (64.55-133.02)	0.1 (0.09-0.11)
Male	5379577 (3669209-7561979)	195.93 (133.71-274.94)	7896838 (5388268-11137855)	199.8 (136.29-281.96)	0.07 (0.07-0.08)
Age					
<5 years	1010859 (687189-1419351)	163.06 (110.85-228.95)	1113595 (756819-1562446)	169.2 (114.99-237.39)	0.13 (0.12-0.14)
5-9 years	931318 (636381-1310658)	159.6 (109.06-224.61)	1129791 (767076-1591202)	164.44 (111.65-231.6)	0.13 (0.12-0.14)
10-14 years	839763 (575216-1184708)	156.76 (107.38-221.16)	1074673 (724429-1513402)	161.21 (108.67-227.02)	0.11 (0.1-0.12)
15-19 years	796264 (542868-1119477)	153.3 (104.51-215.52)	988072 (667651-1389761)	158.35 (107-222.73)	0.1 (0.09-0.11)
20-24 years	740451 (503091-1042237)	150.47 (102.24-211.8)	928297 (628035-1308251)	155.45 (105.17-219.08)	0.09 (0.07-0.11)
25-29 years	664742 (451947-937475)	150.18 (102.11-211.8)	898053 (609591-1264383)	152.64 (103.61-214.91)	0.08 (0.06-0.1)
30-34 years	579874 (394395-821213)	150.45 (102.33-213.07)	904004 (616860-1277149)	149.55 (102.05-211.28)	0.03 (0.01-0.06)
35-39 years	519446 (354304-734723)	147.47 (100.58-208.58)	829035 (562319-1170076)	147.81 (100.26-208.62)	0.02 (0-0.04)
40-44 years	421585 (289677-592767)	147.16 (101.12-206.91)	729517 (496701-1025322)	145.83 (99.29-204.96)	-0.03 (-0.05-0)
45-49 years	332326 (227263-467088)	143.12 (97.88-201.16)	670336 (457577-940973)	141.57 (96.64-198.73)	-0.06 (-0.07–0.04)
50-54 years	294989 (200915-413557)	138.77 (94.52-194.55)	609305 (416883-849407)	136.95 (93.7-190.91)	-0.06 (-0.08–0.03)
55-59 years	245913 (169236-340198)	132.78 (91.38-183.69)	522274 (359523-726503)	131.98 (90.85-183.59)	0.01 (-0.02-0.04)
60-64 years	201660 (139668-279657)	125.56 (86.96-174.12)	408487 (281069-570357)	127.63 (87.82-178.21)	0.06 (0.04-0.09)
65-69 years	137401 (94099-190236)	111.16 (76.13-153.9)	319260 (219230-445941)	115.74 (79.48-161.67)	0.16 (0.14-0.19)
70-74 years	78712 (53820-109351)	92.97 (63.57-129.16)	212943 (145123-296170)	103.45 (70.5-143.88)	0.34 (0.32-0.36)
75-79 years	45984 (31689-64117)	74.7 (51.48-104.16)	113240 (77610-158345)	85.86 (58.85-120.06)	0.51 (0.48-0.54)
80-84 years	19797 (13478-27721)	55.96 (38.1-78.36)	60449 (41243-85362)	69.02 (47.09-97.46)	0.73 (0.7-0.77)
85-89 years	5970 (3980-8484)	39.51 (26.34-56.14)	24260 (16557-34559)	53.06 (36.21-75.59)	1.03 (1-1.06)
90-94 years	1154 (735-1725)	26.93 (17.15-40.25)	6929 (4571-10191)	38.73 (25.55-56.97)	1.25 (1.23-1.27)
95+ years	178 (103-286)	17.52 (10.16-28.1)	1518 (931-2333)	27.85 (17.08-42.8)	1.48 (1.47-1.5)
SDI region					
High-middle SDI	1558810 (1062324-2198989)	145.95 (99.46-205.82)	1870636 (1283602-2637309)	149.98 (102.81-211.41)	0.12 (0.11-0.13)
High SDI	1675311 (1153040-2336379)	195 (134.18-272.18)	2039244 (1402970-2848881)	197.87 (136.14-276.33)	0.06 (0.04-0.07)
Low-middle SDI	1592281 (1096805-2244889)	129.9 (89.59-182.8)	2633269 (1788418-3697132)	133.94 (90.98-188.04)	0.1 (0.09-0.1)
Low SDI	786675 (544352-1109792)	146.11 (101.28-205.15)	1795739 (1229370-2535527)	150.86 (103.45-212.08)	0.11 (0.1-0.12)
Middle SDI	2247820 (1524658-3165425)	125.63 (85.32-176.72)	3196076 (2163936-4515251)	132.25 (89.53-186.65)	0.18 (0.17-0.19)

UI, uncertainty interval; CI, Confidence Interval; DALYs, disability-adjusted life years; EAPC, Estimated Annual Percentage Change; ASD, autism spectrum disorder; SDI, socio-demographic index.

In 2021, the disease burden was higher in males than females. (**Supplementary Figure S1**, **Tables 1**, **2**). Prevalence and DALYs across age groups separately in 2021 were available in **Supplementary Figure S2**. The disease burden decreased with age (**Supplementary Figure S2**, **Tables 1**, **2**).

At the SDI region level, the high SDI region had the most ASRs, and the lowest ASPR and ASDAR were observed in the middle SDI regions (**Supplementary Figure S3**, **Tables 1**, **2**). As for the number of cases, the middle SDI region had the highest number of cases and the low SDI regions had the lowest number of cases. The regular pattern of SDI levels and disease burden was stable across countries and territories. From **Supplementary Figure S4**, the ASRs for prevalence and DALYs showed a “U” relationship with SDI, with the rate being lowest when the SDI was around 0.50.

Across the 54 GBD regions, High-income Asia Pacific ranked the top one in autism spectrum disorders-related ASPR and ASDAR (ASPR: 1559.53, 95% UI: 1311.3-1832.39; ASDAR: 293.92, 95% UI: 203.17-413.01), followed by Australasia and High-income North America. While Tropical Latin America ranked the bottom one (ASPR: 614.52, 95% UI: 514.73-732.29; ASDAR: 114.44, 95% UI: 77.06-160.26), followed by East Asia and Oceania. For the number of cases, the top GBD region was Asia (prevalence: 33291200, 95% UI: 27983185-39014585; DALYs: 6224807, 95% UI: 4229135-8809052), followed by World Bank Lower Middle Income and Limited Health System. The bottom number of cases was observed in Oceania (prevalence: 96966, 95% UI: 81551-116331; DALYs: 18288, 95% UI: 12475-25630), followed by the Caribbean and Australasia (**Supplementary Figure S5**, **Supplementary Tables S1-S2**).

**Figure 1:**
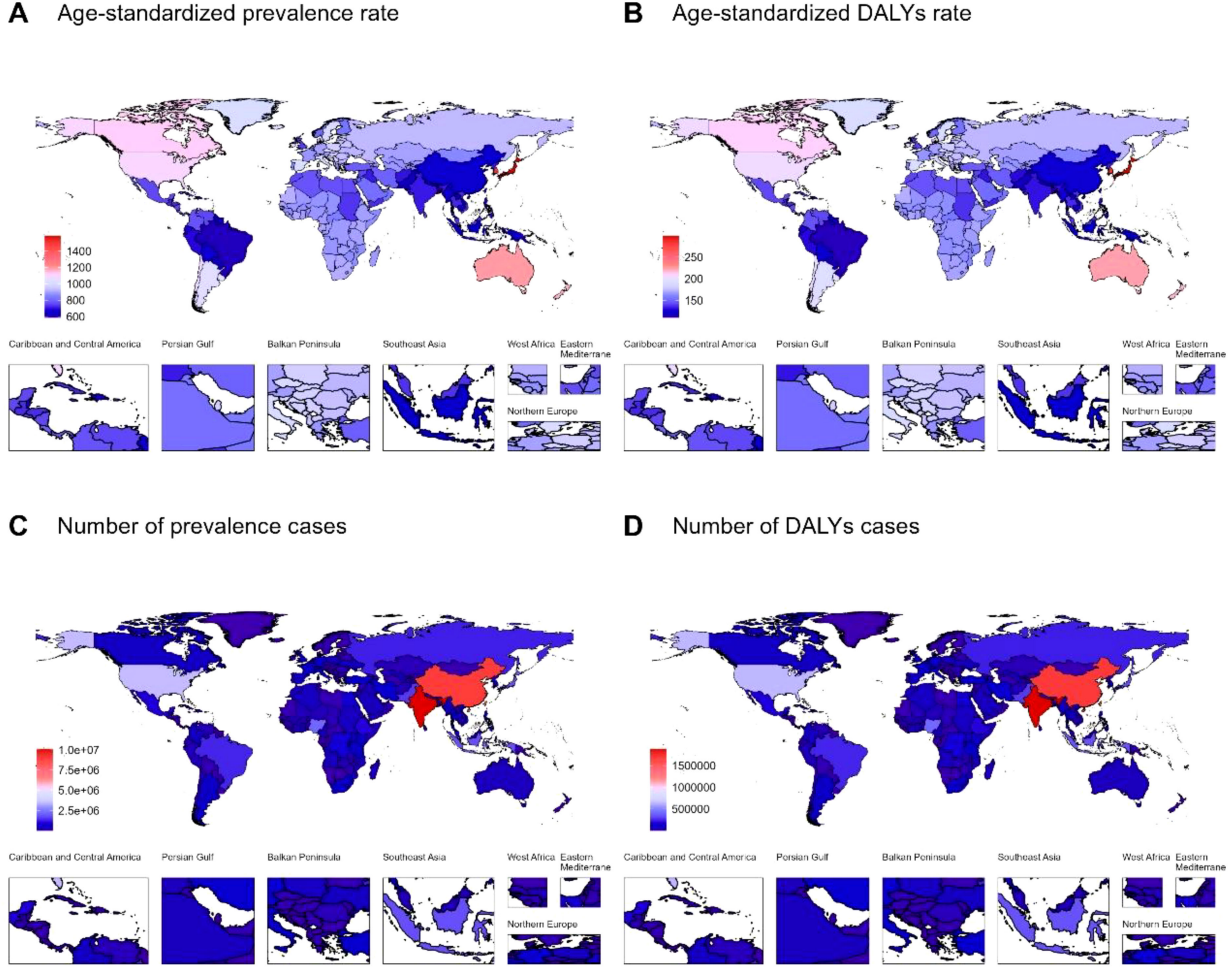
**(A)** Age-standardized prevalence rate, **(B)** Age-standardized DALYs rate, **(C)** Number of prevalence cases, and **(D)** Number of DALYs cases of autism spectrum disorders across countries and territories in 2021. DALYs: disability-adjusted life years.

The disease burden of autism spectrum disorders varied considerably across the world, with the top ASPR and ASDAR observed in Japan (ASPR: 1586.87, 95% UI: 1333.18-1864.1; ASDAR: 299.14, 95% UI: 207.18-420.04), followed by Korea and Singapore. The bottom ASRs of prevalence and DALYs observed in Bangladesh (ASPR: 588.23, 95% UI: 486.73-696.61; ASDAR: 110.26, 95% UI: 75.38-154.62), followed by Brazil. As for the absolute number, the highest number of prevalence (10120042, 95% UI: 8503425-11876619) and DALYs cases (1887143, 95% UI: 1285675-2649048) were observed in India, followed by China and America. And the lowest number of prevalence (10, 95% UI: 8-12) and DALYs cases (2, 95% UI: 1-3) was observed in Tokelau in 2021, followed by Niue and Nauru (**Figure 1**, **Supplementary Tables S1-S2**).

### 3.2 Temporal trend for autism spectrum disorders-related disease burden from 1990 to 2021

The number of prevalence cases of autism spectrum disorders increased from 41929996 (95% UI: 35349448-49633546) in 1990 to 61823540 (95% UI: 52067673-72711238) in 2021 globally. However, the corresponding ASPR changed in the same direction, increasing from 773.25 (95% UI: 651.35-914.71) to 788.34 (95% UI: 663.76-927.21). However, the prevalence estimate followed a different pattern, with an increase in the number of DALYs cases from 7868387 (95% UI: 5351164-11069617) to 11544038 (95% UI: 7842315-16288865), and an increase in ASDAR from 144.51 (95% UI: 98.3-203.28) to 147.56 (95% UI: 100.21-208.16) (**Supplementary Figure S6**, **Tables 1**, **2**).

The trends in males and females alone were consistent with the whole population (**Supplementary Figure S7**, **Tables 1**, **2**). As for the subtype of age group, for the number of cases, the trends for all age groups were the same as global. For ASRs, the trend was also consistent for most age groups except for 40-44 years, 45-49 years, and 50-54 years (**Supplementary Figure S8**, **Tables 1**, **2**). At the SDI region level, the trends for all SDI regions were also consistent with the whole population (**Supplementary Figure S9**, **Tables 1**, **2**).

Across GBD regions, the autism spectrum disorders-related disease burden varied. To find areas with similar variation in disease burden, a hierarchical clustering analysis was conducted in this study. The results are shown in **Supplementary Figure S8**. The significant increase in ASRs occurred in World Bank Upper Middle Income, Commonwealth low-income, Basic Health System, High-income Asia Pacific, East Asia, and Commonwealth High Income, while the significant decrease was in Oceania, Caribbean, Region of the Americas, America, Tropical Latin America, Latin America & Caribbean -WB, Western Sub-Saharan Africa, Western Africa, South Asia -WB, and South Asia (**Supplementary Figure S10**).

Across countries and territories, the most pronounced increase in ASPR from 1990 to 2021 was observed in Equatorial Guinea [EAPC=0.34, 95% confidence interval (CI): 0.32-0.35]. And the most pronounced increase in ASDAR was also in Equatorial Guinea (EAPC=0.39, 95% CI: 0.37-0.41). The most pronounced decrease was observed in Zimbabwe for both ASPR and ASDAR (ASPR: EAPC=-0.09, 95% CI: -0.11–0.08; ASDAR: EAPC=-0.09, 95% CI: -0.11–0.08) (**Supplementary Tables S1-S2**).

### 3.3 The influential factors for EAPC

The association between EAPCs and ASRs in 1990, and HDIs in 2021 was detected, respectively (**Supplementary Figure S11**). The ASRs of autism spectrum disorders in 1990 reflected the disease pool at baseline, and the HDIs in 2021 could be used as the availability of health care and a proxy for the level in each country. A significant association between EAPCs and ASRs of prevalence and DALYs was discovered (prevalence: ρ=0.34, p<0.01; DALYs: ρ=0.32, p<0.01). Moreover, the positive association was also significant between EAPCs and HDIs (prevalence: ρ =0.59, p<0.01; DALYs: ρ=0.56, p<0.01) (**Supplementary Figure S11**).

### 3.4 The predicted results from 2022 to 2046

**Figure 2:**
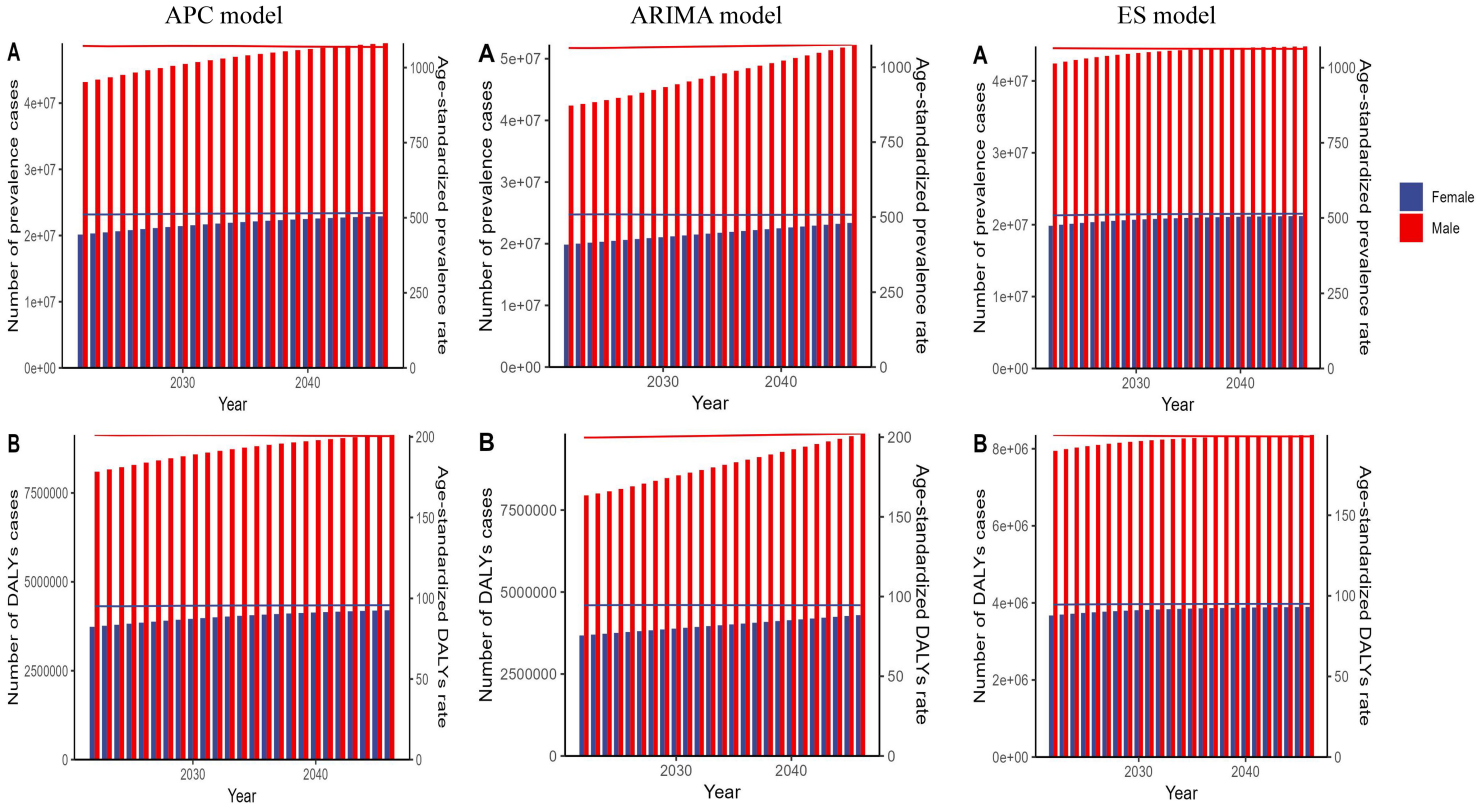
The predicted global results for autism spectrum disorder (ASD)-related numbers and age-standardized rates of prevalence and DALYs by sex from 2022 to 2046, based on the APC model, ARIMA model, and ES model. Panel **(A)** shows the projected number of prevalence cases and age-standardized prevalence rate for ASD. Panel **(B)** shows the projected number of DALYs cases and age-standardized DALYs rate for ASD. DALYs, disability-adjusted life years; APC, age–period–cohort; ARIMA, autoregressive integrated moving average; ES, exponential smoothing.

The predicted results of the APC model showed that the number of prevalence and DALYs cases for both genders would increase from 2022 to 2046. However, the corresponding ASRs will remain relatively stable, with little change, over the next 25 years (**Figure 2**, **Supplementary Table S3**). The results of the ARIMA and ES models remained consistent with the APC model, verifying the stability of the results (**Figure 2**, **Supplementary Tables S4-S5**).

### 3.5 Frontier analysis for ASRs of autism spectrum disorders

**Supplementary Figure S12** presents the unrealized health advancements in ASRs of autism spectrum disorders, stratified by sociodemographic development levels across various countries and regions from 1990 to 2021. Our analysis reveals discernible patterns in the and their respective efficacy disparities across these strata. Notably, as sociodemographic development advances, the effective difference in autism spectrum disorders burden generally escalates, suggesting a heightened potential for improvement among higher SDI nations or regions in 2021. This observation underscores the intricate interplay between societal advancement and autism spectrum disorders health outcomes, necessitating targeted interventions tailored to the unique challenges faced by each developmental tier.

## 4 Discussion

This study utilizes data from GBD 2021 to conduct a comprehensive analysis of the prevalence and DALYs of ASD at global, regional, and national levels from 1990 to 2021 and applies the APC model to predict trends in the disease burden over the next 25 years.

The results indicate that the global prevalence and DALYs of ASD have significantly increased over the past three decades, with the number of ASD cases worldwide rising from 41.93 million in 1990 to 61.82 million in 2021. Although the age-standardized prevalence rate and age-standardized DALY rate have remained relatively stable, the overall increase in the burden suggests that the diagnosis and reporting of ASD have improved globally. This trend reflects positive developments, such as increased public awareness of ASD diagnosis and improvements in healthcare services. However, it also highlights the various challenges countries face in terms of resource allocation and public health policies to address the growing burden of ASD.

From the perspective of global and regional trends in the ASD burden, there has been an overall increasing trend in the prevalence and DALYs of ASD from 1990 to 2021. This increase is closely related to the SDI level, being particularly evident in high-SDI regions, while the increase in ASD burden is less significant in middle and low-SDI regions, with some low-SDI areas even experiencing a decline. The findings of this study are consistent with previous research, which has generally suggested that regional differences in the burden of ASD are closely related to the distribution of healthcare resources, diagnostic capacity, and the level of public awareness about ASD (35, 36). Li et al. (2019) emphasized that socioeconomic development and the quality of healthcare systems significantly influence the reported prevalence of ASD (10).

The global burden of autism spectrum disorder (ASD) has shown a continuous upward trend, particularly in high-SDI regions. Solmi et al. (2022), using GBD 2019 data, reported a consistent increase in ASD prevalence and disease burden worldwide, especially in high-income countries (9). However, their study did not incorporate predictive modeling. In contrast, our study integrated the APC, ARIMA, and Exponential Smoothing models to provide a forward-looking assessment of the ASD burden through 2046. While Li et al. (2022) described the distribution of ASD burden in China and globally, their study did not forecast future trends or conduct stratified analyses by age and gender (10). Our study addressed these gaps, revealing persistent gender differences in ASD burden and a rising trend among the elderly population, particularly among older females. Baxter et al. (2015) highlighted the high rate of underdiagnosis of ASD in low-income countries, which our study further confirmed through frontier efficiency analysis (37). In addition, Lai et al. (2014) pointed out that females with ASD are more likely to engage in “social camouflaging” behaviors, leading to lower diagnostic rates—an observation that aligns closely with our findings on gender differences (38). Elsabbagh et al. (2012) emphasized the lack of ASD screening and surveillance systems in many resource-limited countries, which supports our recommendation to strengthen diagnostic capacity and public awareness in middle- and low-SDI regions (35). Overall, our study expands the analytical depth and temporal scope of ASD burden research and provides more comprehensive evidence to support the development of targeted public health policies across regions with varying levels of social development.

High-SDI regions benefit from better economic conditions, sufficient healthcare resources, and greater public awareness of ASD (39), leading to higher attention to and feasibility of diagnosing the disorder. Additionally, due to improvements in diagnostic capabilities, high-SDI areas have not only correctly reclassified individuals who were previously misdiagnosed with personality disorders as having ASD (40), but they have also extended the diagnostic scope to adults (41) while identifying some common comorbidities associated with ASD. In contrast, low-SDI regions often face various challenges, such as insufficient healthcare resources and lower public awareness (42), resulting in lower diagnostic rates. Therefore, it is necessary for relevant authorities to enhance the accessibility of healthcare services and improve public education to narrow the gap.

From the perspective of gender and age, the prevalence and DALYs of ASD are higher in males than in females, although this gap has gradually narrowed over the past three decades, it still remains significant. This gender difference may be attributed to higher rates of internal difficulties and social camouflaging behaviors in female ASD patients (43, 44), making the diagnosis of ASD in females more challenging. With increasing attention to female ASD in recent years (45, 46), the diagnostic rate for female patients has shown an upward trend. There are also significant differences across age groups. Due to improvements in early screening, early diagnosis of ASD has led to an increased diagnostic rate in children (41, 47), with the 0-4 age group having the highest confirmation rate of ASD. As age increases, the prevalence of ASD gradually decreases, however, the diagnostic rate and DALYs of ASD among individuals aged 60 and older have shown an increasing trend globally. Therefore, it is necessary to develop targeted diagnostic, treatment, and support measures for ASD patients across different age groups in the future, with a particular focus on enhancing the management and care of elderly ASD patients in the context of an aging population.

The prediction results based on the APC model indicate that from 2022 to 2046, the global prevalence and DALYs of ASD will continue to increase in absolute numbers, while the age-standardized rates will remain relatively stable. This trend aligns with population growth and aging, suggesting that the overall burden of ASD will increase in the future, although the distribution across different age groups and regions may remain relatively balanced. However, high-SDI regions will face a greater burden of ASD due to the intensifying aging trend. Therefore, policymakers in high-SDI areas should prioritize increasing the accessibility of ASD diagnosis and treatment services, as well as strengthening targeted support services. Meanwhile, middle- and low-SDI regions need to enhance early diagnosis and intervention measures in the future to mitigate the potential impact of ASD on society.

## 5 Limitations

Although this study provides a detailed analysis of the burden of ASD at global, regional, and national levels, there are some limitations. First, differences in data sources and quality may affect the accuracy of the results, especially in middle- and low-SDI regions where many ASD cases remain undiagnosed and untreated. Second, the significant variation in diagnostic criteria, data collection methods, and public awareness across countries and regions may increase the heterogeneity in estimating the ASD burden. Third, this study does not fully account for the issue of comorbidity between ASD and other mental and neurodevelopmental disorders, which are common among ASD patients (48–50), potentially leading to an underestimation of the ASD burden. Fourth, gender-related differences may contribute to the underdiagnosis and lack of intervention for females with ASD (51, 52), resulting in a possible underestimation of the burden among this group. Therefore, future research should further explore the relationship between ASD and other comorbidities, place more emphasis on the diagnosis and intervention of females with ASD, and incorporate more detailed data from various regions and countries to analyze the specific impact on the burden of ASD.

## 6 Conclusion

This study, by analyzing the burden of ASD (Autism Spectrum Disorder) at global, regional, and national levels from 1990 to 2021, elucidates the temporal trends and key determinants influencing ASD burden across these levels. It provides crucial insights for formulating targeted public health policies and interventions tailored to regions with varying levels of development. The findings indicate that, over the past three decades, the prevalence and associated burden of ASD have significantly increased in high-SDI regions. Although some progress has been made in management and intervention strategies, these efforts have not been sufficient to effectively curb the disability burden associated with ASD.

Future strategies should be tailored to the specific characteristics of ASD, with a focus on disease screening, comorbidities, social support, and public awareness. In high-SDI regions, there is a need to actively develop age-specific treatment strategies that not only address the high prevalence among children but also cater to the treatment needs of ASD patients across other age groups. Conversely, middle- and low-SDI regions should prioritize enhancing public awareness and improving ASD screening efforts to mitigate the associated burden. At the same time, it is recommended that future research adopt interdisciplinary approaches that integrate clinical, social, and policy perspectives to develop more effective strategies for early screening, diagnosis, and intervention (53). Attention should also be given to evaluating the long-term effects of public health interventions across different gender and age groups (54), as well as examining the impact of sociocultural factors and healthcare accessibility on disparities in ASD diagnosis (55, 56), in order to provide a foundation for more inclusive and targeted policy development. Moreover, recent developments in intelligent diagnostic systems demonstrate that hybrid ensemble learning techniques can enhance the accuracy of ASD detection and risk stratification, offering promising directions for personalized screening tools in future research (57).

## Author’s note

The inclusion of any map (including the depiction of any boundaries therein), or of any geographic or locational reference, does not imply the expression of any opinion whatsoever on the part of BMJ concerning the legal status of any country, territory, jurisdiction or area or of its authorities. Any such expression remains solely that of the relevant source and is not endorsed by BMJ. Maps are provided without any warranty of any kind, either express or implied.

## Data Availability

The original contributions presented in the study are included in the article/**Supplementary Material**. Further inquiries can be directed to the corresponding author.
