# Supplementary material for: Age-period-cohort analysis of autism spectrum disorders-related prevalence and DALYs: based on the Global Burden Of Disease Study 2021
Source: Front Psychiatry. 2025 Apr 25;16:1570276. doi: 10.3389/fpsyt.2025.1570276 (PMC12061943; doi:10.3389/fpsyt.2025.1570276)

**Age-Period-Cohort Analysis of Autism Spectrum Disorders-Related Prevalence and DALYs : Based on the Global Burden of Disease Study 2021**

Contents

[Table legend 3](#_Toc31960)

[Table S1. The number of prevalence cases and the age-standardized prevalence rate attributable to autism spectrum disorders in 1990 and 2021, and its trends from 1990 to 2021 globally 3](#_Toc25028)

[Table S2. The number of DALYs cases and the age-standardized DALYs rate attributable to autism spectrum disorders in 1990 and 2021, and its trends from 1990 to 2021 globally. 16](#_Toc10996)

[Table S3. The predicted results in the autism spectrum disorders-related numbers and age-standardized rates of prevalence and DALYs by sex globally from 2022 to 2046 of the APC model. 33](#_Toc26934)

[Table S4. The predicted results in the autism spectrum disorders-related numbers and age-standardized rates of prevalence and DALYs by sex globally from 2022 to 2046 of the ARIMA model. 36](#_Toc7146)

[Table S5. The predicted results in the autism spectrum disorders-related numbers and age-standardized rates of prevalence and DALYs by sex globally from 2022 to 2046 of the ES model 39](#_Toc19089)

[Figure legend 42](#_Toc31943)

[Figure S1. Numbers and age-standardized rates of autism spectrum disorders-related prevalence and DALYs for both sexes in 2021. Abbreviations: DALYs, disability-adjusted life years. 42](#_Toc13398)

[Figure S2. Numbers and age-standardized rates of autism spectrum disorders-related prevalence and DALYs for different age groups in 2021. Abbreviations: DALYs, disability-adjusted life years. 43](#_Toc18695)

[Figure S3. Numbers and age-standardized rates of autism spectrum disorders-related prevalence and DALYs for different SDI regions in 2021. Abbreviations: DALYs, disability-adjusted life years. 44](#_Toc9993)

[Figure S4. Age-standardized rates of prevalence and DALYs attributable to autism spectrum disorders across countries and territories by socio-demographic index for both sexes, 1990-2021. The black line was an adaptive association fitted with adaptive Loess regression based on all data points. Abbreviations: DALYs, disability-adjusted-life-years. 45](#_Toc3631)

[Figure S5. Numbers and age-standardized rates of autism spectrum disorders-related prevalence and DALYs for different GBD regions in 2021. Abbreviations: DALYs, disability-adjusted life years. 46](#_Toc5353)

[Figure S6. Trends in the numbers and age-standardized rates of autism spectrum disorders-related prevalence and DALYs globally from 1990 to 2021. Abbreviations: DALYs, disability-adjusted-life-years. 47](#_Toc19752)

[Figure S7. Trends in the numbers and age-standardized rates of autism spectrum disorders-related prevalence and DALYs globally by sexes from 1990 to 2021. Abbreviations: DALYs, disability-adjusted-life-years. 48](#_Toc31827)

[Figure S8. Trends in the numbers and age-standardized rates of autism spectrum disorders-related prevalence and DALYs globally by age groups from 1990 to 2021. Abbreviations: DALYs, disability-adjusted-life-years. 49](#_Toc28556)

[Figure S9. Trends in the numbers and age-standardized rates of autism spectrum disorders-related prevalence and DALYs globally by SDI regions from 1990 to 2021. Abbreviations: DALYs, disability-adjusted-life-years. 50](#_Toc14949)

[Figure S10. Results of cluster analysis based on the EAPC values of the autism spectrum disorders-related age-standardized rates for prevalence and DALYs from 1990 to 2021. Abbreviations: EAPC, estimated annual percentage change; DALYs, disability-adjusted-life-years. 51](#_Toc14403)

[Figure S11. The association between EAPCs and autism spectrum disorders-related ASRs in 1990 and HDIs in 2021. The circles represent countries that were available on HDI data. The size of the circle is increased with the cases of autism spectrum disorders. The ρ indices and p values presented were derived from Spearman correlation analysis. Abbreviations: EAPC, estimated annual percentage change; ASR, age-standardized rate; HDI, human development index. 52](#_Toc32669)

[Figure S12. Frontier analysis based on SDI and ASRs of the autism spectrum disorders in 2021. The frontier is delineated in solid black color; countries and territories are represented as dots. The top 15 countries with the largest effective difference (largest ASRs gap from the frontier) are labeled in black; examples of frontier countries with low SDI (< 0.5) and low effective difference are labeled in blue, and examples of countries and territories with high SDI (> 0.85) and relatively high effective difference for their level of development are labeled in red. Red dots indicate an increase in age-standardized ASRs from 1990 to 2021; blue dots indicate a decrease in ASRs between 1990 and 2021. Abbreviations: ASRs: Age-Standardized Rates; SDI: Socio-demographic index. 53](#_Toc4643)

# **Table legend**

## Table S1. The number of prevalence cases and the age-standardized prevalence rate attributable to autism spectrum disorders in 1990 and 2021, and its trends from 1990 to 2021 globally

|  | 1990 | | 2021 | |  |
| --- | --- | --- | --- | --- | --- |
|  | Number of prevalence cases (95% UI) | The age-standardized prevalence rate/100000 | Number of prevalence cases (95% UI) | The age-standardized prevalence rate/100000 | EAPC (95% CI) |
|  |  | (95% UI) |  | (95% UI) |  |
| **GBD region** |  |  |  |  |  |
| Advanced Health System | 12990526 (10964619-15269212) | 1001.35 (844.24-1176.17) | 15025803 (12605126-17742386) | 1026.76 (862.07-1207.94) | 0.09 (0.08-0.11) |
| Africa | 5656557 (4753547-6677609) | 848.49 (712.68-1003.24) | 12558402 (10555368-14744420) | 866.78 (728.8-1016.97) | 0.07 (0.07-0.07) |
| African Region | 4696298 (3945520-5538439) | 871.24 (732.26-1029.3) | 10754521 (9053456-12607525) | 885.91 (745.51-1037.19) | 0.05 (0.05-0.06) |
| America | 6190018 (5206462-7321169) | 858.55 (722.5-1015.67) | 8632250 (7265743-10188491) | 853.74 (719.67-1007.16) | 0 (-0.01-0.01) |
| Andean Latin America | 262167 (219787-312866) | 663.53 (556.59-792.24) | 455160 (380330-541452) | 684.26 (571.59-814.13) | 0.1 (0.1-0.11) |
| Asia | 23056533 (19363305-27286760) | 709.76 (596.09-839.16) | 33291200 (27983185-39014585) | 724.24 (608.83-848.89) | 0.07 (0.07-0.08) |
| Australasia | 233589 (194335-276313) | 1162.13 (967.34-1374.82) | 357327 (297947-426205) | 1191 (993.05-1422.73) | 0.08 (0.08-0.09) |
| Basic Health System | 15769556 (13242084-18720239) | 665.45 (557.56-790.23) | 21742720 (18229858-25632744) | 701.47 (588.52-827.31) | 0.19 (0.18-0.2) |
| Caribbean | 247324 (207053-294010) | 687.39 (575.29-816.98) | 320815 (268947-382574) | 682.49 (572.14-813.77) | -0.01 (-0.02--0.01) |
| Central Africa | 617214 (517550-735370) | 866 (725.86-1031.38) | 1566451 (1311928-1856156) | 887.27 (743.86-1049.18) | 0.08 (0.07-0.09) |
| Central Asia | 625276 (524332-739890) | 876.88 (736.06-1038) | 858328 (720725-1011956) | 886.03 (744.24-1044.38) | 0.05 (0.04-0.06) |
| Central Europe | 1155444 (976289-1361825) | 934.71 (789.43-1101.84) | 1055437 (879991-1249997) | 964.38 (809.99-1140.6) | 0.11 (0.11-0.11) |
| Central Latin America | 1275976 (1072627-1515111) | 745.71 (628.42-887.36) | 1917271 (1614924-2268960) | 758.59 (639.01-897.84) | 0.06 (0.05-0.06) |
| Central Sub-Saharan Africa | 507732 (429071-604524) | 865.32 (729.89-1031.01) | 1281661 (1073126-1516828) | 885.37 (739.7-1046.54) | 0.07 (0.06-0.08) |
| Commonwealth High Income | 978063 (822580-1156393) | 903.47 (760.53-1067.34) | 1360411 (1140002-1608794) | 969.81 (814.27-1146.28) | 0.24 (0.22-0.26) |
| Commonwealth Low Income | 1610198 (1350969-1905198) | 722.65 (605.94-854.21) | 3096227 (2600690-3639039) | 762.98 (640.94-900.51) | 0.16 (0.15-0.17) |
| Commonwealth Middle Income | 8865581 (7446848-10478776) | 721.44 (606.15-852.4) | 15752932 (13237481-18493252) | 733.98 (616.74-861.8) | 0.05 (0.04-0.05) |
| East Asia | 7670293 (6401272-9090627) | 618.25 (515.62-732.31) | 9470370 (7884683-11279497) | 660.67 (549.41-785.94) | 0.24 (0.22-0.27) |
| East Asia & Pacific - WB | 13500834 (11286477-15942685) | 713.81 (596.67-842.39) | 17230507 (14454540-20385367) | 736.38 (617.87-869.69) | 0.12 (0.11-0.14) |
| Eastern Africa | 1603130 (1348478-1884036) | 857.61 (722.78-1007.48) | 3575805 (3007836-4186494) | 877.2 (738.32-1024.62) | 0.07 (0.07-0.08) |
| Eastern Europe | 2016644 (1691870-2375404) | 906.09 (760.78-1067.25) | 1828389 (1532203-2170232) | 928.54 (779.82-1102.3) | 0.09 (0.08-0.1) |
| Eastern Mediterranean Region | 2829218 (2376463-3351659) | 719.93 (603.78-854.68) | 5643385 (4724323-6684483) | 731.13 (612.87-865.9) | 0.05 (0.05-0.06) |
| Eastern Sub-Saharan Africa | 1782239 (1498551-2101195) | 874.61 (735.84-1031.59) | 4016785 (3385223-4713345) | 893.46 (752.45-1045.25) | 0.07 (0.06-0.07) |
| Europe | 6957906 (5880210-8211538) | 885.79 (748.03-1044.93) | 7267730 (6093990-8578197) | 902.47 (757.56-1062.7) | 0.07 (0.07-0.08) |
| Europe & Central Asia - WB | 7395393 (6253498-8730015) | 885.17 (748.03-1044.53) | 7920716 (6647411-9354510) | 901.12 (756.93-1061.98) | 0.07 (0.07-0.07) |
| European Region | 7456316 (6304181-8801556) | 884.72 (747.56-1043.96) | 8019188 (6730233-9471633) | 900 (756.23-1060.78) | 0.07 (0.06-0.07) |
| High-income Asia Pacific | 2475304 (2088848-2899501) | 1442.14 (1217.6-1688.66) | 2681978 (2253495-3157801) | 1559.53 (1311.3-1832.39) | 0.25 (0.23-0.27) |
| High-income North America | 2964103 (2498734-3501887) | 1072.08 (904.82-1265.08) | 3892297 (3248391-4605299) | 1097.16 (919.18-1296.68) | 0.1 (0.08-0.13) |
| Latin America & Caribbean - WB | 3253382 (2736864-3873957) | 720.14 (606.09-856.82) | 4763564 (4006581-5646492) | 726.36 (610.82-861.09) | 0.03 (0.03-0.03) |
| Limited Health System | 11898987 (9997218-14072308) | 722.17 (606.75-853.96) | 21917488 (18405895-25669865) | 742.79 (624.18-870.11) | 0.08 (0.08-0.09) |
| Middle East & North Africa - WB | 2035807 (1709825-2406297) | 759.93 (637.22-898.91) | 3750633 (3154791-4426890) | 776.32 (652.44-916.06) | 0.07 (0.07-0.08) |
| Minimal Health System | 1230927 (1032900-1466790) | 854.24 (716.96-1016.83) | 3088844 (2588455-3636928) | 865.32 (723.68-1017.55) | 0.05 (0.04-0.05) |
| North Africa and Middle East | 2667505 (2236579-3150684) | 755.54 (633.94-893.03) | 4884140 (4104186-5765658) | 771.8 (648.48-910.69) | 0.07 (0.07-0.08) |
| North America | 2963887 (2498512-3501670) | 1072.02 (904.76-1265.02) | 3892114 (3248232-4605100) | 1097.11 (919.16-1296.64) | 0.1 (0.08-0.13) |
| Northern Africa | 947745 (789529-1121543) | 754.88 (628.65-895.12) | 1644098 (1380720-1938997) | 769.11 (645.61-906.95) | 0.06 (0.06-0.06) |
| Oceania | 46427 (38901-54939) | 678.31 (568.57-803.5) | 96966 (81551-116331) | 673.2 (566.15-807.01) | -0.03 (-0.03--0.02) |
| Region of the Americas | 6190018 (5206462-7321169) | 858.55 (722.5-1015.67) | 8632250 (7265743-10188491) | 853.74 (719.67-1007.16) | 0 (-0.01-0.01) |
| South-East Asia Region | 9231924 (7737819-10938927) | 679.72 (569.86-804.68) | 14329586 (12060141-16827762) | 689.34 (580.33-809.62) | 0.04 (0.03-0.05) |
| South Asia | 7795864 (6537961-9216359) | 681.98 (571.95-806.02) | 12848945 (10802916-15015893) | 686.16 (576.62-801.99) | 0.01 (0-0.02) |
| South Asia - WB | 7997829 (6707378-9453570) | 682.94 (572.76-807.01) | 13252845 (11142735-15494373) | 687.45 (577.62-803.82) | 0.01 (0.01-0.02) |
| Southeast Asia | 3201991 (2696864-3790421) | 663.62 (559.77-784.78) | 4784650 (4024215-5673822) | 682.98 (574.58-809.75) | 0.1 (0.09-0.11) |
| Southern Africa | 871285 (731837-1034712) | 881.01 (740.13-1048.94) | 1703509 (1432096-1994976) | 892.54 (750.94-1046.93) | 0.04 (0.03-0.05) |
| Southern Latin America | 516198 (432594-614214) | 1035.24 (867.49-1231.97) | 700575 (586987-826296) | 1056.55 (885.87-1245.37) | 0.05 (0.04-0.05) |
| Southern Sub-Saharan Africa | 488296 (410059-576133) | 890.22 (746.83-1049.8) | 741683 (623701-878038) | 903.63 (760.08-1069.17) | 0.06 (0.04-0.07) |
| Sub-Saharan Africa - WB | 4724005 (3971219-5575226) | 871.11 (732.19-1028.28) | 10944024 (9210458-12831440) | 884.3 (743.72-1035.65) | 0.05 (0.05-0.05) |
| Tropical Latin America | 960545 (799233-1140397) | 608.68 (506.46-723.02) | 1381083 (1156544-1646422) | 614.52 (514.73-732.29) | 0.03 (0.03-0.04) |
| Western Africa | 1617182 (1357883-1900301) | 880.28 (738.8-1037.12) | 4068540 (3420294-4805412) | 885.69 (743.66-1045.16) | 0.02 (0.01-0.03) |
| Western Europe | 3247150 (2744661-3836563) | 876.83 (740.75-1034.33) | 3672369 (3082109-4334850) | 896.6 (751.64-1054.5) | 0.09 (0.08-0.09) |
| Western Pacific Region | 11277581 (9426672-13315201) | 723.29 (604.16-853.8) | 14161867 (11861717-16791895) | 750.54 (628.69-888.95) | 0.14 (0.13-0.16) |
| Western Sub-Saharan Africa | 1789928 (1503876-2104809) | 879.6 (737.7-1035.65) | 4577312 (3847537-5399715) | 886.16 (745.12-1045.59) | 0.03 (0.02-0.03) |
| World Bank High Income | 10460711 (8838072-12302523) | 1036.81 (875.22-1217.62) | 12511348 (10508660-14775420) | 1060.87 (890.99-1247.5) | 0.09 (0.07-0.1) |
| World Bank Low Income | 2728999 (2295451-3233429) | 829.47 (695.41-983.1) | 6193519 (5202881-7285612) | 851.84 (715.7-999.36) | 0.09 (0.09-0.1) |
| World Bank Lower Middle Income | 14868398 (12486538-17606100) | 715.16 (600.8-846.82) | 25446353 (21395979-29956699) | 730.58 (614.58-860.21) | 0.06 (0.06-0.07) |
| World Bank Upper Middle Income | 13831719 (11576588-16432278) | 679.89 (569.15-807.24) | 17623487 (14782180-20875859) | 711.39 (597.47-841.63) | 0.17 (0.16-0.18) |
| **Country** |  |  |  |  |  |
| Afghanistan | 72336 (60253-86235) | 692.1 (575.23-826.9) | 234672 (193720-279519) | 703.12 (581.75-839.48) | 0.07 (0.06-0.07) |
| Albania | 31026 (26155-36829) | 916.04 (772.38-1086.81) | 24352 (20150-28733) | 943.96 (781.58-1112.33) | 0.12 (0.11-0.13) |
| Algeria | 200022 (166706-241034) | 759.24 (632.49-913.81) | 346139 (288435-412073) | 774.58 (644.9-921.94) | 0.07 (0.07-0.07) |
| America | 2667576 (2253831-3150947) | 1069.13 (904.49-1261.27) | 3491378 (2919750-4128390) | 1094.82 (920.23-1291.25) | 0.11 (0.08-0.14) |
| American Samoa | 358 (299-425) | 711.11 (592.79-847.53) | 355 (294-424) | 708.93 (588.86-847.66) | -0.02 (-0.03--0.02) |
| Andorra | 496 (412-592) | 914.03 (760.13-1092.8) | 743 (617-885) | 905.87 (752.86-1077.54) | -0.04 (-0.05--0.03) |
| Angola | 95122 (79450-112839) | 867.77 (724.37-1030.27) | 305831 (256806-359178) | 879.18 (740.95-1029.71) | 0.04 (0.03-0.05) |
| Antigua and Barbuda | 428 (356-505) | 705.08 (586.29-833.04) | 627 (518-744) | 711.62 (588-844.88) | 0.06 (0.04-0.07) |
| Arab Egypt | 431931 (360259-514118) | 747.73 (621.69-890.28) | 827877 (692545-982173) | 763.69 (639.63-905.84) | 0.06 (0.05-0.07) |
| Arab Emirates | 16390 (13614-19767) | 833.09 (690.19-1003.49) | 85811 (71622-101887) | 840.88 (701.01-999.6) | 0.08 (0.06-0.11) |
| Argentine Republic | 342394 (285559-409124) | 1030.07 (859.04-1231.02) | 467333 (387115-549821) | 1045.7 (867.27-1230.92) | 0.03 (0.02-0.03) |
| Armenia | 31561 (26458-37485) | 907.34 (761.6-1078.68) | 26787 (22228-31693) | 921.98 (766.63-1090.48) | 0.08 (0.07-0.09) |
| Australia | 194956 (161736-231515) | 1166.62 (968.16-1385.34) | 298553 (248636-355954) | 1195.41 (996.09-1428.67) | 0.08 (0.08-0.09) |
| Austria | 65473 (54497-77607) | 878.44 (732.39-1040.64) | 75244 (62297-89954) | 892.21 (741.13-1069.09) | 0.05 (0.04-0.06) |
| Azerbaijan | 65698 (54706-77962) | 872.16 (726.43-1035.7) | 93774 (78203-110267) | 893.02 (745.06-1051.18) | 0.1 (0.09-0.11) |
| Bahamas | 1832 (1517-2201) | 692.48 (574.15-829.25) | 2673 (2208-3176) | 692.93 (572.22-823.3) | 0.01 (0.01-0.02) |
| Bahrain | 4316 (3591-5102) | 815.65 (678.52-964) | 13388 (11123-15891) | 848.43 (703.66-1009.13) | 0.17 (0.15-0.18) |
| Bangladesh | 666537 (556520-794367) | 580.22 (484.37-691.08) | 977775 (809418-1157419) | 588.23 (486.73-696.61) | 0 (-0.02-0.02) |
| Barbados | 1768 (1472-2086) | 706.37 (588.28-833.51) | 2029 (1683-2431) | 712.36 (593.84-854.61) | 0.03 (0.03-0.03) |
| Belarus | 94187 (78559-111908) | 917.67 (765.03-1090.09) | 84113 (69832-100487) | 945.19 (783.5-1128.08) | 0.1 (0.09-0.11) |
| Belgium | 84343 (70594-100897) | 878.99 (736.28-1050.83) | 95502 (79179-113504) | 885.28 (734.81-1051.9) | 0.02 (0.02-0.03) |
| Belize | 1330 (1100-1581) | 684.31 (567.88-813.89) | 2986 (2484-3592) | 683 (567.11-821.9) | 0 (-0.01-0) |
| Benin | 44949 (37432-53258) | 867.18 (722.19-1030.14) | 126908 (105326-150724) | 888.97 (736.62-1059.52) | 0.07 (0.07-0.08) |
| Bermuda | 429 (355-513) | 727.21 (602.12-867.96) | 443 (366-534) | 740.51 (611.86-893.56) | 0.07 (0.07-0.07) |
| Bhutan | 4255 (3551-5047) | 637.27 (531.97-757.04) | 4965 (4142-5901) | 648.15 (540.63-770.35) | 0.07 (0.06-0.08) |
| Bolivia | 43394 (36216-51497) | 646.57 (538.71-766.88) | 79586 (66690-95487) | 663.11 (555.09-795.5) | 0.09 (0.08-0.09) |
| Bosnia and Herzegovina | 43123 (35887-50697) | 951.14 (791.57-1118.61) | 30578 (25322-36194) | 972 (806.75-1149.98) | 0.1 (0.09-0.12) |
| Botswana | 12325 (10301-14677) | 881.44 (735.94-1054.07) | 22308 (18648-26512) | 905.99 (757.3-1077.23) | 0.1 (0.08-0.12) |
| Brazil | 934492 (777483-1110612) | 608.37 (506.14-722.58) | 1335516 (1118732-1591670) | 614.1 (514.55-731.89) | 0.03 (0.03-0.04) |
| Brunei Darussalam | 3662 (3050-4305) | 1359.1 (1129.11-1600.21) | 6268 (5277-7473) | 1370.47 (1153.07-1635.38) | 0.03 (0.01-0.04) |
| Bulgaria | 78307 (65612-94198) | 925.09 (776.17-1114.28) | 59412 (49351-70647) | 936.75 (782.91-1115.07) | 0.04 (0.03-0.05) |
| Burkina Faso | 87805 (73835-104346) | 863.64 (727.61-1027.34) | 211002 (177560-248825) | 875.73 (736.92-1033.98) | 0.05 (0.05-0.06) |
| Burundi | 51108 (42748-60772) | 860.58 (719.82-1024.88) | 123763 (103230-145723) | 886.52 (737.51-1042.95) | 0.12 (0.11-0.13) |
| Cabo Verde | 3339 (2831-3982) | 904.28 (765.2-1080.85) | 5388 (4489-6409) | 951.58 (792.38-1131.87) | 0.19 (0.18-0.2) |
| Cambodia | 70004 (58503-84033) | 641.57 (536.73-770.14) | 117092 (97585-140257) | 672.42 (560.56-805.84) | 0.16 (0.15-0.17) |
| Cameroon | 97118 (81027-114905) | 876.74 (727.48-1038.19) | 298747 (250342-353675) | 893.12 (744.39-1057.65) | 0.07 (0.06-0.08) |
| Canada | 295882 (246053-351690) | 1099.56 (915.15-1307.16) | 400293 (333013-471587) | 1117.86 (930.35-1317.62) | 0.05 (0.04-0.06) |
| Central African Republic | 24766 (20659-29370) | 846.56 (706.43-1003.07) | 49603 (41732-60053) | 848.75 (711.72-1026.09) | 0.01 (0-0.01) |
| Chad | 55234 (46126-65881) | 860.41 (716.61-1025.86) | 166040 (138215-198286) | 876.22 (727.5-1049.33) | 0.07 (0.06-0.08) |
| Chile | 141563 (118050-166519) | 1046.44 (871.62-1231.58) | 198698 (165530-234571) | 1082.57 (902.84-1276.97) | 0.1 (0.09-0.1) |
| China | 7347264 (6120001-8699968) | 612.61 (509.84-724.96) | 9078613 (7559642-10816937) | 655.75 (545.06-780.37) | 0.25 (0.23-0.27) |
| Colombia | 252558 (212456-300644) | 751.38 (632.03-895.39) | 374548 (311161-447535) | 771.79 (640.88-921.54) | 0.09 (0.08-0.09) |
| Congo | 352737 (295645-419465) | 865.44 (722.01-1028.8) | 844114 (703189-1006901) | 887.69 (738.2-1055.6) | 0.09 (0.08-0.11) |
| Cook Islands | 141 (117-169) | 727.82 (604.65-874.21) | 126 (104-151) | 724.6 (601.42-867.41) | -0.02 (-0.04--0.01) |
| Costa Rica | 24663 (20404-29587) | 788.64 (652.13-947.28) | 37148 (30730-44470) | 791.99 (655.18-946.79) | 0.01 (0.01-0.01) |
| Croatia | 46323 (38654-54776) | 966.66 (806.57-1142.12) | 39608 (32808-47258) | 996 (824.91-1186.18) | 0.1 (0.1-0.11) |
| Cuba | 78021 (64693-92298) | 717.9 (595.15-849.35) | 77815 (64831-92479) | 723.4 (603.16-859.95) | 0.03 (0.03-0.04) |
| Cyprus | 6793 (5724-8116) | 875.34 (737.91-1045.7) | 11580 (9600-13763) | 882.87 (733.66-1048) | 0.04 (0.04-0.05) |
| Czech Republic | 96907 (80255-114752) | 962.03 (797.26-1138.44) | 100751 (83247-118532) | 996.73 (824.21-1172.93) | 0.11 (0.1-0.12) |
| C么te d'Ivoire | 115096 (96423-136282) | 888.56 (740.87-1051.76) | 265435 (219442-316288) | 909.13 (752.86-1083.92) | 0.08 (0.07-0.09) |
| Denmark | 45191 (38275-53453) | 918.48 (778.12-1086.15) | 52193 (43800-61841) | 946.47 (797.22-1124.2) | 0.09 (0.07-0.11) |
| Djibouti | 3997 (3348-4783) | 909.17 (759.3-1089.86) | 12141 (10115-14422) | 931.61 (775.89-1106.11) | 0.08 (0.07-0.09) |
| Dominica | 509 (423-606) | 689.48 (572.17-821.96) | 459 (384-548) | 694.96 (582.66-829.61) | 0.03 (0.03-0.04) |
| Dominican Republic | 49487 (41205-59262) | 665.44 (553.27-799.73) | 74959 (62615-89409) | 677.06 (565.54-807.54) | 0.06 (0.05-0.08) |
| Eastern Uruguay | 32217 (27042-38370) | 1043.41 (875.95-1243.31) | 34505 (28690-41437) | 1058.09 (880.2-1268.18) | 0.03 (0.02-0.04) |
| Ecuador | 69266 (58083-83089) | 668.02 (560.33-803.21) | 124017 (102804-147821) | 682.66 (565.93-813.82) | 0.07 (0.06-0.08) |
| El Salvador | 40417 (33789-48158) | 731.66 (610.1-872.11) | 48189 (40115-57745) | 745.63 (620.56-893.76) | 0.06 (0.06-0.07) |
| Equatorial Guinea | 3846 (3206-4559) | 849.19 (706.01-1010.85) | 14881 (12415-17591) | 927.42 (773.14-1096.92) | 0.34 (0.32-0.35) |
| Eritrea | 31859 (26703-38227) | 865.64 (722.75-1040.4) | 61870 (51571-73194) | 886.77 (738.32-1053.81) | 0.08 (0.08-0.09) |
| Estonia | 14203 (11916-16881) | 926.38 (777.84-1100.49) | 11867 (9951-14118) | 964.47 (811.2-1146.72) | 0.15 (0.14-0.16) |
| Eswatini | 7528 (6301-8964) | 873.36 (728.39-1044.62) | 10732 (8942-12795) | 886.61 (735.76-1057.88) | 0.06 (0.04-0.07) |
| Ethiopia | 467650 (394757-550491) | 860.94 (726.24-1019.19) | 1026009 (860805-1209152) | 894.56 (751.87-1053.84) | 0.12 (0.12-0.12) |
| Federated States of Micronesia | 737 (614-878) | 679.34 (566.27-809.43) | 717 (603-846) | 683.16 (575.01-804.04) | 0.03 (0.02-0.04) |
| Fiji | 5406 (4522-6438) | 685.75 (573.43-817.87) | 6422 (5310-7678) | 684.81 (566.38-818.42) | 0 (-0.01-0) |
| Finland | 40024 (33355-47868) | 827.94 (691.03-989.16) | 42267 (34965-50426) | 828.74 (687.47-990.5) | 0.04 (0.03-0.05) |
| French Republic | 487796 (407831-579420) | 872.54 (729.22-1036.75) | 547708 (455312-652227) | 881.28 (734.92-1049.95) | 0.05 (0.04-0.05) |
| Gabonese Republic | 9105 (7640-10762) | 886.79 (744.61-1048.69) | 16981 (14176-20233) | 905.35 (756.28-1079.37) | 0.07 (0.06-0.07) |
| Gambia | 9262 (7725-10977) | 892.96 (742.59-1059.73) | 22558 (18921-26659) | 897.96 (752.13-1063.47) | 0.02 (0.01-0.02) |
| Georgia | 49434 (41136-59053) | 902.31 (751.23-1077.69) | 31347 (26097-37274) | 910.72 (760.44-1082.46) | 0.03 (0.02-0.04) |
| Germany | 671042 (562926-804755) | 879.28 (737.68-1053.25) | 709083 (583887-846724) | 897.46 (738.7-1070.96) | 0.05 (0.04-0.05) |
| Ghana | 140039 (116549-167986) | 883.97 (733.61-1059.16) | 320038 (267854-380839) | 894.61 (748.04-1064.17) | 0.03 (0.03-0.04) |
| Grand Duchy of Luxembourg | 3273 (2738-3910) | 887.45 (742.21-1060.26) | 5594 (4662-6713) | 901.78 (749.72-1081.87) | 0.06 (0.06-0.06) |
| Great Britain and Northern Ireland | 377328 (316088-445627) | 687.64 (575.46-813.07) | 481779 (404014-573252) | 750.84 (631.59-890.28) | 0.33 (0.27-0.39) |
| Greenland | 577 (485-687) | 1008.24 (847.24-1197.17) | 565 (470-667) | 1016.89 (848.62-1199.33) | 0.04 (0.03-0.04) |
| Grenada | 606 (504-725) | 684.08 (568.63-818) | 712 (590-843) | 697.88 (578.37-825.47) | 0.07 (0.07-0.07) |
| Guam | 1035 (861-1241) | 736.09 (611.78-884.1) | 1134 (937-1363) | 725.96 (600.28-871.38) | -0.02 (-0.03--0.02) |
| Guatemala | 63386 (52476-75397) | 713.09 (591.99-849.11) | 117068 (97641-138674) | 725.96 (605.46-861.03) | 0.06 (0.06-0.06) |
| Guinea | 54608 (45601-65516) | 863.89 (721.42-1037.55) | 124211 (103674-147569) | 874.94 (729.46-1040.49) | 0.03 (0.03-0.04) |
| Guinea-Bissau | 9188 (7660-11066) | 850.53 (707.31-1023.71) | 18975 (15913-22763) | 862.51 (721.04-1036.45) | 0.05 (0.05-0.06) |
| Guyana | 5414 (4469-6471) | 662.92 (546.02-791.95) | 5142 (4284-6116) | 663.18 (552.76-788.96) | 0 (-0.01-0.01) |
| Haiti | 42181 (35138-50624) | 625.1 (519.62-749.87) | 83918 (69937-99848) | 626.99 (522.66-746.25) | 0.03 (0.02-0.03) |
| Hashemite Jordan | 30936 (25822-36773) | 791.49 (661.05-943.3) | 101898 (84712-120872) | 809.99 (672.75-960.49) | 0.1 (0.08-0.11) |
| Hellenic Republic | 89003 (74727-105660) | 883.65 (742.14-1047.04) | 82360 (68115-98856) | 881.41 (732.46-1056.8) | -0.02 (-0.03-0) |
| Honduras | 36200 (30073-43348) | 727.68 (604.45-873.32) | 75743 (63116-89760) | 727.82 (605.76-864.21) | 0 (0-0.01) |
| Hungary | 95236 (79699-112669) | 942.16 (787.85-1115.44) | 87803 (72633-104569) | 971.04 (803.53-1153.07) | 0.09 (0.08-0.1) |
| Iceland | 2590 (2178-3081) | 1028.65 (865.65-1223.78) | 3547 (2960-4182) | 1047.28 (875.08-1235.01) | 0.06 (0.05-0.07) |
| India | 6260916 (5254094-7405450) | 702.56 (589.74-830.55) | 10120042 (8503425-11876619) | 708.08 (595.12-831.15) | 0.02 (0.01-0.02) |
| Indonesia | 1213580 (1013694-1434441) | 631.25 (526.9-746.45) | 1820130 (1528694-2163015) | 647.27 (543.65-768.98) | 0.1 (0.09-0.11) |
| Iran | 458913 (387004-544339) | 771.11 (651.92-915.74) | 677140 (567413-799868) | 791.24 (662.91-933.97) | 0.08 (0.07-0.09) |
| Iraq | 133932 (109988-159429) | 694.07 (568.66-826.52) | 300186 (250707-360994) | 709.19 (590.71-852.81) | 0.06 (0.05-0.07) |
| Ireland | 36948 (30804-44058) | 1034.27 (862.31-1233.97) | 51255 (42768-60994) | 1071.93 (894.78-1274.45) | 0.13 (0.1-0.15) |
| Israel | 39570 (33067-46975) | 794.89 (664.1-943.6) | 75028 (62800-88989) | 795.14 (665.37-944.01) | 0 (-0.01-0.02) |
| Italy | 534487 (449992-631992) | 979.59 (823.65-1157.15) | 548536 (460422-649272) | 999.28 (839.61-1185.98) | 0.08 (0.06-0.09) |
| Jamaica | 16866 (14158-19940) | 700.08 (587.2-828.76) | 19465 (16351-23130) | 702.54 (590.22-834.66) | 0.02 (0.02-0.03) |
| Japan | 1797367 (1514035-2116209) | 1458.58 (1229.73-1714.68) | 1846133 (1544551-2177718) | 1586.87 (1333.18-1864.1) | 0.28 (0.25-0.31) |
| Kazakhstan | 147271 (122840-176991) | 880.02 (734.49-1057.8) | 171145 (143571-203083) | 897.74 (752.82-1064.89) | 0.09 (0.07-0.12) |
| Kenya | 221155 (185458-260230) | 897.62 (755.15-1056.98) | 474307 (398522-555166) | 906.28 (761.41-1061.04) | 0.01 (0-0.02) |
| Kiribati | 509 (424-608) | 649.98 (539.54-777.73) | 814 (677-973) | 647.32 (537.56-772.52) | -0.01 (-0.02--0.01) |
| Korea | 143960 (119718-170949) | 686.12 (571.03-815.3) | 183546 (152523-219567) | 706.1 (587.08-844.95) | 0.18 (-0.82-1.18) |
| Kuwait | 14869 (12312-17791) | 840.65 (694.22-1006.66) | 38953 (32200-46278) | 829.51 (685.6-984.33) | -0.05 (-0.07--0.02) |
| Kyrgyz Republic | 40145 (33573-47838) | 872.1 (729.38-1040.18) | 62364 (52333-74525) | 888.76 (745.97-1060.8) | 0.06 (0.06-0.07) |
| Lao Republic | 28274 (23548-33649) | 643.39 (535.82-764.53) | 50662 (42266-60801) | 668.8 (557.38-802.73) | 0.13 (0.12-0.15) |
| Latvia | 23773 (19847-28378) | 918.45 (768.27-1095.6) | 16377 (13504-19373) | 943.23 (778.73-1117.1) | 0.07 (0.06-0.08) |
| Lebanese Republic | 25906 (21693-30733) | 846.52 (709.01-1003.56) | 47951 (39803-56800) | 872.45 (724.16-1033.25) | 0.11 (0.1-0.12) |
| Lesotho | 13825 (11562-16586) | 851.6 (710.46-1020.29) | 17023 (14232-20353) | 871.04 (727.68-1041.34) | 0.07 (0.06-0.07) |
| Liberia | 22758 (19203-27013) | 877.58 (739.11-1043.62) | 51735 (43028-61381) | 905.25 (752.73-1072.19) | 0.15 (0.13-0.16) |
| Libya | 33671 (28062-39958) | 771.85 (642.93-918.83) | 53424 (44282-63557) | 769.06 (636.68-915.26) | 0.02 (0.01-0.03) |
| Lithuania | 33550 (27741-39695) | 928.8 (767.98-1098.85) | 23950 (19818-28268) | 944.8 (783.9-1115.81) | 0.04 (0.03-0.05) |
| Madagascar | 111477 (93176-132636) | 883.24 (737.85-1048.26) | 267842 (223705-317606) | 889.47 (741.97-1051.67) | 0.03 (0.02-0.03) |
| Malawi | 91231 (75905-109257) | 871.25 (723.54-1047.13) | 182220 (152822-215383) | 886.03 (741.64-1048.18) | 0.05 (0.04-0.05) |
| Malaysia | 129759 (108016-154305) | 711.35 (592.39-846.36) | 235032 (195861-280303) | 734.32 (611.89-874.55) | 0.11 (0.1-0.12) |
| Maldives | 1615 (1342-1935) | 698.49 (580.61-838.01) | 4089 (3364-4872) | 765.9 (629.95-912.24) | 0.32 (0.29-0.35) |
| Mali | 79881 (67185-94928) | 867.97 (728.32-1029.97) | 226328 (189443-269355) | 884.95 (739.11-1054.3) | 0.07 (0.06-0.08) |
| Malta | 3240 (2696-3852) | 884.33 (736.39-1050.67) | 3704 (3094-4423) | 898.1 (751.35-1069.75) | 0.06 (0.05-0.06) |
| Marshall Islands | 325 (272-388) | 677.52 (568.38-809.66) | 392 (326-467) | 677.25 (561.99-808.13) | 0 (0-0.01) |
| Mauritania | 19191 (15817-22813) | 886.21 (731.29-1052.2) | 41862 (35113-49571) | 914.27 (763.12-1085.73) | 0.1 (0.1-0.11) |
| Mauritius | 8020 (6658-9561) | 715.1 (593.86-853.3) | 9036 (7492-10706) | 728.09 (603.14-862.45) | 0.04 (0.03-0.05) |
| Mexican States | 662985 (555619-785858) | 745.02 (626.57-883.13) | 980052 (828327-1166731) | 758.52 (641.28-902.84) | 0.05 (0.05-0.06) |
| Moldova | 39947 (33239-47513) | 895.3 (744.77-1064.58) | 31504 (26231-37071) | 917.94 (766.03-1082.27) | 0.07 (0.07-0.08) |
| Monaco | 247 (206-293) | 893.2 (746.22-1056.59) | 308 (255-367) | 890.6 (744.5-1062.46) | -0.02 (-0.03-0) |
| Mongolia | 19165 (15935-22558) | 847.17 (704.09-997.56) | 29425 (24829-35121) | 861.31 (726.44-1027.39) | 0.08 (0.07-0.1) |
| Montenegro | 6037 (5035-7210) | 963.27 (803.47-1150.28) | 5858 (4865-6893) | 976.32 (811.05-1150.05) | 0.07 (0.06-0.08) |
| Morocco | 196172 (162992-235172) | 746.04 (619.88-893.93) | 282540 (237025-335302) | 756.23 (634.1-897.49) | 0.05 (0.04-0.05) |
| Mozambique | 122539 (102315-146678) | 861.8 (720.43-1030.32) | 289253 (241019-343910) | 872.61 (724.97-1038.97) | 0.02 (0.02-0.03) |
| Myanmar | 273455 (227001-326755) | 650.3 (540.24-778.49) | 381152 (316476-457387) | 668.77 (555.58-803) | 0.1 (0.09-0.11) |
| Namibia | 13093 (10931-15560) | 883.34 (735.89-1047.41) | 22796 (19064-27185) | 904.97 (757.19-1079.09) | 0.08 (0.07-0.09) |
| Nauru | 72 (60-86) | 681.69 (567.8-807.23) | 77 (64-92) | 672.26 (560.12-802.92) | -0.04 (-0.06--0.03) |
| Nepal | 127977 (106473-152739) | 623.72 (518.05-743.76) | 198854 (165182-238529) | 626.14 (519.44-750.58) | 0 (-0.01-0.01) |
| Netherlands | 130285 (107750-154426) | 894.65 (740.7-1060.63) | 146299 (120487-174361) | 899.61 (739.79-1073.19) | 0.02 (0.02-0.03) |
| New Zealand | 38633 (32090-45962) | 1140 (947.15-1356.21) | 58774 (48727-70501) | 1169.19 (969.87-1404.29) | 0.08 (0.08-0.09) |
| Nicaragua | 30344 (25373-36406) | 741.63 (620.27-889.82) | 51312 (42553-60565) | 756.13 (627.38-892.39) | 0.07 (0.06-0.07) |
| Niger | 74797 (62273-89565) | 870.38 (720.35-1047.26) | 235100 (195619-278660) | 877.2 (729.39-1038.9) | 0.04 (0.03-0.04) |
| Nigeria | 832153 (698504-974704) | 884.25 (741.08-1039.17) | 2150983 (1809589-2536050) | 881.92 (743.52-1040.43) | -0.01 (-0.02-0) |
| Niue | 16 (13-19) | 695.43 (577.24-827.38) | 12 (10-14) | 701.63 (583.96-827.24) | 0.03 (0.03-0.04) |
| North Macedonia | 18691 (15648-22053) | 933.51 (781.4-1100.96) | 20305 (17006-24110) | 957.46 (802.97-1136.94) | 0.11 (0.1-0.12) |
| Northern Mariana Islands | 339 (283-403) | 733.39 (609-868.59) | 356 (296-424) | 734.2 (611.61-874.78) | -0.01 (-0.03-0.01) |
| Norway | 32917 (27741-38806) | 809.34 (682.01-954.42) | 41725 (34854-49755) | 808.26 (677.13-963.13) | 0.04 (0.03-0.06) |
| Pakistan | 736179 (616472-871153) | 630.12 (527.23-746.56) | 1547309 (1290751-1819389) | 631.1 (526.62-741.88) | 0 (-0.01-0) |
| Palau | 109 (91-130) | 701.79 (583.41-835.15) | 130 (108-153) | 726.67 (601.89-857.42) | 0.1 (0.07-0.12) |
| Palestine | 16507 (13809-19660) | 766.87 (640.6-915.09) | 41421 (34527-49407) | 782.09 (651.43-934.14) | 0.05 (0.05-0.06) |
| Panama | 18771 (15592-22289) | 766.06 (635.86-910.16) | 33372 (28006-39445) | 779.7 (654.36-921.4) | 0.04 (0.03-0.05) |
| Papua New Guinea | 28911 (24145-34649) | 672.74 (561.65-804.6) | 72756 (61047-87244) | 669.79 (562-804.3) | -0.02 (-0.02--0.02) |
| Paraguay | 26053 (21519-30936) | 621.12 (513.19-738.29) | 45567 (37819-54286) | 628.14 (521.37-748.16) | 0.04 (0.03-0.04) |
| Peru | 149507 (125268-177563) | 666.22 (556.14-793.7) | 251558 (210109-300501) | 691.7 (577.66-826.15) | 0.13 (0.13-0.13) |
| Philippines | 446232 (375503-529658) | 679.59 (573.39-806.93) | 792254 (665372-936397) | 686.12 (576.44-811.24) | 0.02 (0.01-0.04) |
| Poland | 353459 (297907-420246) | 935.19 (787.63-1111.68) | 350686 (292313-416249) | 964.54 (806.74-1144.42) | 0.11 (0.1-0.11) |
| Portuguese Republic | 83441 (69839-99799) | 847.08 (709.21-1013.93) | 83820 (69544-99210) | 861.83 (718.31-1019.83) | 0.05 (0.04-0.06) |
| Puerto Rico | 26066 (21486-31117) | 721.76 (594.83-861.61) | 22419 (18496-26891) | 731.13 (602.82-877.6) | 0.06 (0.06-0.07) |
| Qatar | 4319 (3606-5147) | 926.74 (773.09-1106.16) | 30335 (25129-36018) | 964.65 (797.95-1145.07) | 0.24 (0.21-0.28) |
| Romania | 210327 (176039-249143) | 910.05 (762.02-1077.33) | 167952 (140239-198085) | 939 (786.04-1105.08) | 0.12 (0.11-0.13) |
| Russian Federation | 1342411 (1129810-1588282) | 902.18 (759-1067.77) | 1286225 (1079417-1526745) | 928.79 (779.96-1100.57) | 0.11 (0.09-0.12) |
| Rwanda | 66448 (55421-78980) | 861.27 (718.95-1024.94) | 123933 (103559-146886) | 890.72 (742.57-1054.95) | 0.15 (0.12-0.17) |
| Saint Kitts and Nevis | 287 (236-346) | 683.01 (562.13-825.87) | 403 (337-478) | 694.87 (581.7-824.31) | 0.06 (0.05-0.08) |
| Saint Lucia | 971 (807-1164) | 689.09 (573.05-827.34) | 1214 (1004-1452) | 702.43 (581.63-837.43) | 0.07 (0.06-0.07) |
| Saint Vincent andGrenadines | 775 (645-919) | 685.96 (570.53-816.43) | 778 (646-927) | 693.71 (575.61-825.82) | 0.04 (0.03-0.04) |
| Samoa | 1226 (1014-1459) | 696.32 (574.84-827.96) | 1524 (1271-1814) | 696.88 (580.53-829.85) | 0.01 (0-0.01) |
| San Marino | 206 (171-246) | 895.54 (745.58-1070.3) | 270 (223-322) | 890.81 (739.01-1063.57) | -0.01 (-0.01--0.01) |
| Sao Tome and Principe | 1143 (950-1363) | 897.62 (744.03-1075.17) | 2070 (1721-2435) | 923.45 (767.81-1089.22) | 0.11 (0.09-0.12) |
| Saudi Arabia | 130756 (109553-155236) | 793.47 (663.17-943.93) | 316319 (261846-377152) | 817.81 (677.69-975.73) | 0.12 (0.1-0.13) |
| Senegal | 71101 (59508-84368) | 879.54 (735.86-1044.94) | 149225 (124110-176875) | 899.7 (746.59-1068.32) | 0.07 (0.07-0.08) |
| Serbia | 89176 (74170-104678) | 932.01 (775.05-1093.18) | 82418 (67984-98177) | 968.42 (801.38-1155.37) | 0.14 (0.14-0.15) |
| Seychelles | 525 (435-630) | 709.19 (587.47-852.7) | 771 (639-917) | 733.9 (607.91-873.25) | 0.12 (0.11-0.13) |
| Sierra Leone | 38180 (31962-45394) | 874.66 (732.39-1039.03) | 82630 (68797-98693) | 889.28 (739.88-1062.46) | 0.07 (0.05-0.08) |
| Singapore | 44192 (37095-52496) | 1429.2 (1199.68-1698.53) | 83478 (70079-99156) | 1487.3 (1250.01-1767.84) | 0.14 (0.12-0.16) |
| Slovak Republic | 49394 (40880-58194) | 942.61 (780.95-1110.12) | 50365 (42150-60085) | 966.67 (809-1150.8) | 0.09 (0.08-0.09) |
| Slovenia | 18961 (15814-22353) | 976.29 (815.33-1150.31) | 19987 (16579-24003) | 1017.93 (845.74-1224.1) | 0.16 (0.15-0.17) |
| Solomon Islands | 2410 (2007-2888) | 675.84 (562.35-810.88) | 4774 (4000-5681) | 671.8 (562.75-801.57) | -0.02 (-0.03--0.02) |
| Somalia | 75133 (62671-89365) | 874.9 (727.83-1039.89) | 203174 (170536-238936) | 868.11 (725.96-1020.78) | -0.01 (-0.01-0) |
| South Africa | 344036 (289552-404948) | 892.63 (751.04-1050.07) | 524999 (440584-623272) | 911.75 (765.21-1082.26) | 0.09 (0.07-0.11) |
| South Sudan | 55807 (46632-66212) | 901.39 (753.22-1067.78) | 91201 (76433-108723) | 891.21 (745.85-1060.42) | -0.03 (-0.04--0.02) |
| Spain | 370330 (309281-439125) | 982.87 (821.86-1167.46) | 435737 (366879-512824) | 1024.75 (863.79-1204.31) | 0.15 (0.12-0.18) |
| Sri Lanka | 128014 (105636-152382) | 730.14 (602.29-870.3) | 165139 (136817-197366) | 749.9 (620.3-896.03) | 0.09 (0.07-0.11) |
| Sudan | 152596 (126295-181965) | 724.3 (599.45-862.53) | 332468 (276576-395890) | 737.46 (611.59-879.45) | 0.05 (0.05-0.06) |
| Sultanate of Oman | 16825 (14020-20102) | 814.09 (677.25-974.06) | 40757 (33695-48903) | 832.93 (687.45-998.59) | 0.07 (0.05-0.09) |
| Suriname | 2686 (2231-3202) | 674.28 (560.01-803.7) | 3873 (3215-4627) | 673.75 (559.5-804.66) | 0.01 (0-0.02) |
| Sweden | 80246 (66356-97503) | 984.03 (813.53-1197.76) | 98896 (81105-118051) | 1016.68 (839.54-1216.85) | 0.13 (0.12-0.15) |
| Swiss Confederation | 59214 (49019-70607) | 894.54 (740.55-1065.24) | 75957 (62946-90332) | 902.31 (749.22-1073.81) | 0.04 (0.04-0.04) |
| Syrian Arab Republic | 101374 (84015-121565) | 761.43 (632.33-913.31) | 107525 (89279-129371) | 758.95 (630.04-913.04) | 0 (-0.04-0.03) |
| Taiwan (Province of China) | 179069 (149586-212375) | 868.78 (725.42-1029.97) | 208211 (171450-247999) | 911.13 (751.99-1083.88) | 0.16 (0.13-0.19) |
| Tajikistan | 48444 (40372-57829) | 863.96 (719.9-1028.98) | 90860 (76072-107163) | 866.8 (726.92-1023.56) | 0.01 (0.01-0.02) |
| Tanzania | 241370 (202090-288516) | 879.69 (736.6-1049.73) | 550748 (460151-654251) | 897.53 (749.11-1067.5) | 0.05 (0.05-0.06) |
| Thailand | 406111 (336913-487825) | 698.48 (578.68-839.01) | 464142 (384301-556047) | 722.16 (597.62-864.49) | 0.11 (0.1-0.12) |
| Timor-Leste | 5503 (4605-6547) | 668.41 (558.6-793.98) | 9752 (8149-11610) | 677.29 (566.1-807.23) | 0.05 (0.04-0.06) |
| Togolese Republic | 34026 (28281-40578) | 871.78 (724.83-1040.26) | 78022 (65401-93122) | 887.25 (744.13-1059.9) | 0.06 (0.05-0.07) |
| Tokelau | 11 (9-14) | 698.5 (583.4-833.57) | 10 (8-12) | 714.48 (598.7-855.62) | 0.09 (0.08-0.1) |
| Tonga | 711 (588-846) | 692.71 (572.38-823.21) | 754 (624-899) | 694.86 (575.59-829.3) | 0 (-0.01-0.01) |
| Trinidad and Tobago | 8538 (7121-10201) | 692.06 (577.71-826.75) | 9479 (7921-11319) | 699.19 (585.67-835.94) | 0.04 (0.04-0.05) |
| Tunisia | 66759 (55500-80208) | 776.65 (644.31-932.51) | 92257 (76510-109576) | 784.98 (650.94-932.34) | 0.04 (0.04-0.04) |
| Turkey | 452114 (376966-537059) | 762.79 (635.65-908.08) | 652685 (541549-773512) | 786.61 (652.92-931.67) | 0.1 (0.1-0.11) |
| Turkmenistan | 33429 (28076-39571) | 864.9 (725.79-1024.71) | 46417 (38815-54767) | 884.64 (740.28-1043.24) | 0.08 (0.07-0.08) |
| Tuvalu | 64 (54-77) | 653.55 (544.95-783.74) | 86 (71-103) | 684.44 (566.42-820.67) | 0.15 (0.14-0.16) |
| Uganda | 162738 (135098-194245) | 881.96 (730.45-1052.78) | 415361 (347108-492485) | 900.12 (749.74-1067.56) | 0.07 (0.06-0.08) |
| Ukraine | 468573 (391469-553634) | 912.89 (764.01-1078.45) | 374354 (311842-446635) | 922.29 (770.57-1101.32) | 0.04 (0.03-0.05) |
| Union ofComoros | 4357 (3638-5121) | 887.7 (739.21-1042.14) | 6943 (5795-8222) | 906.94 (757.46-1074.45) | 0.08 (0.07-0.09) |
| Uzbekistan | 190129 (158446-225568) | 872.71 (725.84-1035.96) | 306208 (255218-361269) | 879.41 (733.15-1035.35) | 0.04 (0.04-0.05) |
| Vanuatu | 1085 (897-1289) | 680.3 (562.96-809.79) | 2176 (1802-2605) | 671.08 (555.49-802.39) | -0.04 (-0.04--0.03) |
| Venezuela | 146652 (122250-175830) | 750.73 (624.53-900.5) | 199838 (167417-237716) | 756.72 (634.16-899.52) | 0.05 (0.04-0.07) |
| Viet Nam | 486268 (400306-578674) | 687.36 (565.65-818.74) | 728725 (608388-866947) | 726.31 (606.27-863.65) | 0.17 (0.16-0.18) |
| Virgin Islands | 756 (633-898) | 702.36 (587.6-834.2) | 567 (469-675) | 709.87 (588.29-848.51) | 0.04 (0.03-0.05) |
| Yemen | 105404 (87801-126475) | 724.62 (601.45-867.3) | 255839 (214344-304154) | 726.59 (608.13-859.7) | 0.01 (0-0.02) |
| Zambia | 74097 (61614-88699) | 874.98 (725.21-1047.76) | 184523 (153717-218566) | 896.06 (747.27-1063.85) | 0.09 (0.07-0.1) |
| Zimbabwe | 97489 (81522-115928) | 890.66 (742.15-1057.56) | 143825 (120090-171167) | 873.92 (728.89-1041.39) | -0.09 (-0.11--0.08) |

UI: uncertainty interval; CI: Confidence Interval; EAPC: Estimated Annual Percentage Change; ASD: autism spectrum disorder; SDI: socio-demographic index

## **Table S2.** The number of DALYs cases and the age-standardized DALYs rate attributable to autism spectrum disorders in 1990 and 2021, and its trends from 1990 to 2021 globally.

|  | 1990 | | 2021 | |  |  |
| --- | --- | --- | --- | --- | --- | --- |
|  | Number of DALYs cases (95% UI) | The age-standardized DALYs rate/100000 (95% UI) | Number of DALYs cases (95% UI) | The age-standardized DALYs rate/100000 (95% UI) | EAPC (95% CI) |  |
|  |  |  |  |  |  |  |
| **GBD region** |  |  |  |  |  |  |
| Advanced Health System | 2423749 (1664338-3386171) | 187.77 (128.91-262.7) | 2772835 (1908510-3866620) | 192.22 (132.2-267.9) | 0.09 (0.08-0.1) |  |
| Africa | 1060855 (730074-1497762) | 157.69 (108.76-221.86) | 2364115 (1606497-3329203) | 161.73 (110.04-227.04) | 0.09 (0.09-0.1) |  |
| African Region | 880006 (604250-1237854) | 161.72 (111.32-226.62) | 2025005 (1379883-2852528) | 165.25 (112.85-232.38) | 0.08 (0.08-0.09) |  |
| America | 1158363 (793206-1624943) | 160.22 (109.72-224.82) | 1597536 (1089385-2240514) | 159.1 (108.47-223.29) | 0 (-0.01-0.01) |  |
| Andean Latin America | 49443 (33619-69538) | 124.27 (84.44-174.38) | 85530 (57762-120400) | 128.5 (86.85-180.92) | 0.12 (0.11-0.12) |  |
| Asia | 4339398 (2949456-6105993) | 132.88 (90.37-186.66) | 6224807 (4229135-8809052) | 135.85 (92.27-192.07) | 0.08 (0.08-0.09) |  |
| Australasia | 43363 (29954-60392) | 216.66 (149.69-301.73) | 66006 (45662-93212) | 222.63 (154-314.31) | 0.09 (0.08-0.09) |  |
| Basic Health System | 2980786 (2031226-4204986) | 125.18 (85.36-176.42) | 4072393 (2765675-5736160) | 132.1 (89.68-185.81) | 0.2 (0.19-0.21) |  |
| Caribbean | 46475 (31845-65212) | 128.66 (88.23-180.49) | 59774 (40541-83420) | 127.53 (86.45-178.12) | -0.01 (-0.02--0.01) |  |
| Central Africa | 115326 (80099-161196) | 160.04 (111.33-224.46) | 294822 (200530-418102) | 165.23 (112.36-233.34) | 0.11 (0.1-0.13) |  |
| Central Asia | 117929 (79958-165161) | 164.48 (111.59-230.63) | 161331 (109690-228533) | 166.43 (113.16-235.64) | 0.06 (0.05-0.07) |  |
| Central Europe | 215366 (147013-301194) | 175.07 (119.47-245.25) | 194783 (134725-272380) | 181.04 (125.2-252.95) | 0.12 (0.12-0.13) |  |
| Central Latin America | 240950 (166889-339190) | 139.53 (96.56-196.46) | 358883 (242903-507855) | 142.22 (96.18-201.27) | 0.06 (0.06-0.06) |  |
| Central Sub-Saharan Africa | 94796 (66178-133560) | 159.71 (111.53-225.55) | 241038 (164257-340305) | 164.69 (112.41-231.89) | 0.11 (0.09-0.12) |  |
| Commonwealth High Income | 182428 (125384-253145) | 169.54 (116.71-235.61) | 251642 (172361-353424) | 181.71 (124.29-256.22) | 0.24 (0.22-0.26) |  |
| Commonwealth Low Income | 302210 (207545-425270) | 134.31 (92.29-188.34) | 582912 (397386-817688) | 142.75 (97.38-199.95) | 0.19 (0.18-0.2) |  |
| Commonwealth Middle Income | 1656808 (1138023-2333264) | 133.81 (92.02-188.03) | 2945212 (2010056-4139835) | 136.79 (93.37-192.13) | 0.07 (0.06-0.07) |  |
| East Asia | 1454162 (985458-2057248) | 116.87 (79.27-165.28) | 1774089 (1197771-2499467) | 125.12 (84.47-176.31) | 0.25 (0.23-0.27) |  |
| East Asia & Pacific - WB | 2551073 (1736501-3591263) | 134.4 (91.54-189.02) | 3224267 (2201814-4549486) | 138.96 (94.98-195.63) | 0.13 (0.12-0.15) |  |
| Eastern Africa | 300720 (207490-423174) | 159.29 (110.3-223.02) | 674524 (461139-948067) | 163.87 (112.31-230.08) | 0.1 (0.1-0.11) |  |
| Eastern Europe | 374961 (256220-526611) | 169.49 (115.51-238.15) | 337179 (231683-471167) | 173.87 (119.23-243.41) | 0.1 (0.09-0.12) |  |
| Eastern Mediterranean Region | 532655 (366519-743955) | 134.33 (92.57-187.86) | 1058001 (718046-1485831) | 136.32 (92.47-191.32) | 0.06 (0.05-0.06) |  |
| Eastern Sub-Saharan Africa | 334049 (230022-470394) | 162.3 (112.1-227.7) | 757537 (516796-1069116) | 166.8 (114.11-234.89) | 0.1 (0.09-0.11) |  |
| Europe | 1296843 (885365-1825097) | 166.04 (113.38-234.04) | 1343850 (920756-1872382) | 169.27 (115.8-235.92) | 0.08 (0.08-0.08) |  |
| Europe & Central Asia - WB | 1379389 (941091-1940340) | 165.9 (113.21-233.67) | 1466805 (1003099-2049929) | 169.01 (115.38-236.38) | 0.08 (0.07-0.08) |  |
| European Region | 1390863 (948833-1955950) | 165.82 (113.15-233.5) | 1485248 (1015758-2076348) | 168.81 (115.25-236.17) | 0.08 (0.07-0.08) |  |
| High-income Asia Pacific | 464059 (321081-645771) | 271.65 (187.69-378.09) | 495601 (343282-695132) | 293.92 (203.17-413.01) | 0.25 (0.23-0.28) |  |
| High-income North America | 551460 (380091-766012) | 200.5 (138.2-278.78) | 713184 (493088-997383) | 203.95 (141.1-285.5) | 0.09 (0.06-0.11) |  |
| Latin America & Caribbean - WB | 612054 (422484-860350) | 134.55 (92.88-188.98) | 888697 (600003-1253404) | 135.91 (91.68-191.63) | 0.04 (0.03-0.04) |  |
| Limited Health System | 2226362 (1529284-3143637) | 134.04 (92.16-188.67) | 4108006 (2803118-5778011) | 138.62 (94.62-194.7) | 0.11 (0.1-0.11) |  |
| Middle East & North Africa - WB | 384066 (264358-539580) | 142.05 (97.78-198.92) | 702363 (473460-988509) | 145.04 (97.84-203.91) | 0.08 (0.07-0.08) |  |
| Minimal Health System | 230001 (158805-322074) | 158.05 (109.39-221.02) | 581731 (397090-821312) | 161.14 (110.14-227.16) | 0.07 (0.07-0.08) |  |
| North Africa and Middle East | 502914 (345825-705164) | 141.22 (97.21-197.39) | 914908 (616066-1286313) | 144.2 (97.15-202.52) | 0.08 (0.07-0.08) |  |
| North America | 551419 (380063-765950) | 200.49 (138.19-278.76) | 713150 (493063-997333) | 203.95 (141.1-285.49) | 0.09 (0.06-0.11) |  |
| Northern Africa | 178806 (122872-251767) | 141.27 (96.97-198.2) | 308777 (207538-432827) | 143.96 (96.79-201.79) | 0.07 (0.06-0.07) |  |
| Oceania | 8749 (6036-12347) | 126.72 (87.41-178.75) | 18288 (12475-25630) | 126.07 (85.71-176.76) | -0.02 (-0.02--0.02) |  |
| Region of the Americas | 1158363 (793206-1624943) | 160.22 (109.72-224.82) | 1597536 (1089385-2240514) | 159.1 (108.47-223.29) | 0 (-0.01-0.01) |  |
| South-East Asia Region | 1728285 (1188479-2435782) | 126.34 (86.96-177.59) | 2677838 (1814948-3763935) | 128.79 (87.3-180.99) | 0.06 (0.06-0.07) |  |
| South Asia | 1456554 (1002832-2044748) | 126.43 (87.11-177.07) | 2399066 (1637235-3382018) | 127.86 (87.27-180.25) | 0.03 (0.03-0.04) |  |
| South Asia - WB | 1494448 (1029068-2098845) | 126.63 (87.27-177.41) | 2474926 (1688424-3491216) | 128.11 (87.41-180.55) | 0.03 (0.03-0.04) |  |
| Southeast Asia | 604596 (412518-853227) | 124.46 (84.99-174.66) | 900293 (612066-1272740) | 128.65 (87.45-181.69) | 0.12 (0.11-0.13) |  |
| Southern Africa | 163478 (112051-231296) | 163.8 (112.48-230.07) | 319181 (216774-448730) | 165.76 (112.7-232.45) | 0.05 (0.04-0.06) |  |
| Southern Latin America | 96939 (66932-136362) | 194.17 (134.02-273.06) | 130654 (88773-184968) | 198.18 (134.67-280.3) | 0.05 (0.04-0.05) |  |
| Southern Sub-Saharan Africa | 91839 (63144-129133) | 165.99 (114.4-232.95) | 138087 (94596-194320) | 167.72 (114.91-236.11) | 0.05 (0.03-0.07) |  |
| Sub-Saharan Africa - WB | 884906 (608156-1244329) | 161.65 (111.37-226.56) | 2060971 (1405667-2904802) | 164.92 (112.77-231.89) | 0.08 (0.07-0.08) |  |
| Tropical Latin America | 179905 (124306-252234) | 113.25 (78.17-158.68) | 255968 (172573-358108) | 114.44 (77.06-160.26) | 0.05 (0.04-0.05) |  |
| Western Africa | 302524 (207127-425312) | 163.29 (112.02-229.22) | 766812 (525679-1078389) | 165.39 (113.5-231.95) | 0.05 (0.05-0.06) |  |
| Western Europe | 604909 (412912-853062) | 164.69 (112.59-232.43) | 678957 (466623-944266) | 168.26 (115.5-234.77) | 0.09 (0.08-0.09) |  |
| Western Pacific Region | 2131265 (1451929-2998175) | 136.28 (92.89-191.47) | 2647820 (1803027-3737563) | 141.71 (96.57-199.53) | 0.15 (0.13-0.17) |  |
| Western Sub-Saharan Africa | 334968 (229599-471462) | 163.21 (112.07-229.13) | 862884 (590900-1215654) | 165.49 (113.48-232.43) | 0.05 (0.05-0.06) |  |
| World Bank High Income | 1902686 (1308264-2654869) | 194.58 (133.75-271.81) | 2264178 (1561785-3159669) | 198.62 (136.94-277.08) | 0.08 (0.07-0.1) |  |
| World Bank Low Income | 547633 (377312-771424) | 153.91 (106.26-216.03) | 1230913 (840143-1742405) | 158.63 (108.44-223.55) | 0.11 (0.1-0.12) |  |
| World Bank Lower Middle Income | 2693530 (1846477-3780155) | 133.1 (91.37-186.37) | 4615574 (3131886-6484938) | 136.61 (92.72-192) | 0.08 (0.08-0.09) |  |
| World Bank Upper Middle Income | 2702475 (1834562-3809383) | 127.82 (86.82-180.18) | 3417020 (2327143-4814147) | 133.94 (91.19-188.32) | 0.18 (0.17-0.19) |  |
| **Country** |  |  |  |  |  |  |
| Afghanistan | 13486 (9305-18960) | 127.69 (88.01-180.06) | 44251 (30327-62061) | 130.4 (88.99-183.19) | 0.09 (0.08-0.1) |  |
| Albania | 5855 (4003-8261) | 171.81 (117.81-242.95) | 4525 (3081-6351) | 177.55 (121.66-249.56) | 0.14 (0.13-0.15) |  |
| Algeria | 37870 (25640-53393) | 142.32 (96.47-201.1) | 65019 (43430-90405) | 145.25 (97.29-202) | 0.07 (0.07-0.08) |  |
| America | 495965 (341955-691923) | 199.81 (137.79-278.91) | 639136 (443188-891390) | 203.32 (141.04-283.66) | 0.09 (0.07-0.12) |  |
| American Samoa | 68 (47-97) | 133.69 (92.24-190.29) | 66 (46-95) | 132.67 (91.13-189.41) | -0.03 (-0.03--0.03) |  |
| Andorra | 93 (64-131) | 171.74 (118.33-243.12) | 137 (95-192) | 170.08 (116.15-239.28) | -0.04 (-0.05--0.03) |  |
| Angola | 17892 (12655-25001) | 161.43 (113.89-226.14) | 57738 (39908-80383) | 163.75 (114.18-227.43) | 0.06 (0.04-0.07) |  |
| Antigua and Barbuda | 80 (55-112) | 132.16 (89.7-184.94) | 117 (80-163) | 133.19 (91.15-187.11) | 0.05 (0.04-0.07) |  |
| Arab Egypt | 81337 (56168-114586) | 139.78 (96.37-196.45) | 156034 (105617-220041) | 143.04 (96.73-202.42) | 0.07 (0.07-0.08) |  |
| Arab Emirates | 3097 (2113-4329) | 156.21 (106.68-217.66) | 15980 (10922-22626) | 157.17 (107.79-220.92) | 0.08 (0.05-0.1) |  |
| Argentine Republic | 64278 (43927-89923) | 193.21 (132.06-270.21) | 87303 (59403-122255) | 196.26 (133.8-274.55) | 0.03 (0.02-0.03) |  |
| Armenia | 5939 (4052-8353) | 170.29 (116.36-239.1) | 4988 (3417-6919) | 173.14 (118.22-240.44) | 0.09 (0.08-0.1) |  |
| Australia | 36204 (24960-50585) | 217.6 (150.11-303.88) | 55151 (38177-77960) | 223.52 (154.96-315.02) | 0.09 (0.08-0.09) |  |
| Austria | 12200 (8282-16943) | 165.05 (112.02-229.34) | 13943 (9445-19713) | 167.75 (113.17-238.39) | 0.06 (0.05-0.06) |  |
| Azerbaijan | 12420 (8386-17541) | 164.13 (110.9-232.06) | 17600 (12253-24508) | 168.14 (117.3-234.12) | 0.11 (0.1-0.12) |  |
| Bahamas | 346 (238-479) | 129.99 (89.21-179.9) | 498 (339-694) | 129.79 (88.24-180.93) | 0.01 (0-0.02) |  |
| Bahrain | 817 (557-1146) | 153.13 (104.96-214.6) | 2503 (1745-3484) | 158.7 (110.48-221.9) | 0.16 (0.15-0.18) |  |
| Bangladesh | 125391 (85921-176640) | 108.08 (73.99-152.02) | 183526 (125202-257161) | 110.26 (75.38-154.62) | 0.03 (0.01-0.04) |  |
| Barbados | 332 (226-473) | 132.87 (90.78-188.89) | 376 (254-528) | 133.56 (90.21-188.68) | 0.03 (0.02-0.03) |  |
| Belarus | 17589 (12181-24668) | 172.34 (119.05-242.05) | 15544 (10639-21490) | 177.3 (121.19-247.72) | 0.11 (0.1-0.12) |  |
| Belgium | 15721 (10731-21815) | 165.26 (112.9-230.15) | 17685 (12069-24824) | 166.22 (113.16-232.53) | 0.02 (0.01-0.03) |  |
| Belize | 252 (175-352) | 128.56 (89.14-179.15) | 561 (377-783) | 127.85 (86.13-179.03) | -0.01 (-0.02-0) |  |
| Benin | 8437 (5903-11898) | 160.94 (112.73-225.39) | 23975 (16662-33538) | 166.22 (115.57-231.03) | 0.1 (0.09-0.1) |  |
| Bermuda | 81 (56-112) | 136.94 (95.22-190.8) | 82 (56-115) | 139.17 (94.57-196.88) | 0.07 (0.06-0.07) |  |
| Bhutan | 802 (548-1135) | 119.11 (81.2-169.75) | 931 (651-1301) | 121.32 (84.92-169.56) | 0.09 (0.08-0.09) |  |
| Bolivia | 8185 (5523-11500) | 121 (81.87-168.79) | 14908 (10362-20975) | 123.92 (86.2-173.97) | 0.1 (0.09-0.1) |  |
| Bosnia and Herzegovina | 8060 (5525-11485) | 177.98 (121.75-253.23) | 5632 (3931-7947) | 182.25 (127.54-258.48) | 0.11 (0.09-0.13) |  |
| Botswana | 2326 (1623-3264) | 164.62 (115.16-228.24) | 4160 (2926-5843) | 168.36 (118.46-236.33) | 0.1 (0.08-0.12) |  |
| Brazil | 174993 (121003-245475) | 113.17 (78.18-158.65) | 247435 (166839-346062) | 114.35 (77.03-160.11) | 0.05 (0.04-0.05) |  |
| Brunei Darussalam | 695 (488-969) | 255.64 (178.51-358.06) | 1178 (806-1646) | 257.72 (176.33-361.1) | 0.03 (0.02-0.04) |  |
| Bulgaria | 14537 (9805-20311) | 173.31 (117.16-242.34) | 10929 (7592-15387) | 175.74 (121.76-249.13) | 0.05 (0.04-0.07) |  |
| Burkina Faso | 16387 (11349-23116) | 159.84 (110.35-224.74) | 39778 (27898-55873) | 163.69 (114.95-228.44) | 0.1 (0.09-0.1) |  |
| Burundi | 9602 (6440-13646) | 160.27 (107.91-225.36) | 23381 (16201-32782) | 165.68 (114.79-232.66) | 0.13 (0.12-0.15) |  |
| Cabo Verde | 632 (439-886) | 169.9 (117.52-236.85) | 1012 (695-1423) | 178.5 (122.85-251.32) | 0.2 (0.19-0.21) |  |
| Cambodia | 13169 (8820-18261) | 119.64 (80.75-166) | 22078 (14960-31395) | 126.39 (85.77-179.76) | 0.19 (0.18-0.2) |  |
| Cameroon | 18253 (12805-25571) | 163.06 (114.53-227.58) | 56435 (38499-79141) | 166.98 (114.28-232.94) | 0.1 (0.08-0.11) |  |
| Canada | 55374 (38372-76807) | 206.83 (143.08-286.5) | 73932 (51632-104753) | 209.56 (146.93-296.03) | 0.05 (0.04-0.05) |  |
| Central African Republic | 4613 (3183-6516) | 155.97 (108.43-218.5) | 9291 (6298-13238) | 157.38 (106.83-223.7) | 0.04 (0.03-0.04) |  |
| Chad | 10350 (7167-14469) | 159.8 (110.89-224.52) | 31313 (21435-44203) | 163.31 (112.88-229.58) | 0.08 (0.07-0.09) |  |
| Chile | 26616 (18531-37107) | 196.09 (136.64-273.59) | 36926 (25392-52667) | 202.81 (139.62-289.4) | 0.1 (0.09-0.1) |  |
| China | 1392966 (943870-1971071) | 115.81 (78.58-163.69) | 1700739 (1146590-2397647) | 124.19 (83.7-175.1) | 0.26 (0.24-0.28) |  |
| Colombia | 47678 (32948-67939) | 140.81 (97.25-200.03) | 70109 (47445-98658) | 145.02 (98.14-204.33) | 0.1 (0.09-0.1) |  |
| Congo | 4165 (2850-5884) | 161.45 (111.11-226.6) | 9415 (6499-13233) | 166.79 (115.33-234.33) | 0.12 (0.1-0.14) |  |
| Cook Islands | 27 (18-38) | 137 (93.17-194.75) | 23 (16-33) | 135.89 (92.42-189.65) | -0.03 (-0.05--0.02) |  |
| Costa Rica | 4671 (3212-6577) | 148.2 (101.83-208.6) | 6936 (4738-9759) | 148.6 (101.53-208.88) | 0.01 (0-0.01) |  |
| Croatia | 8640 (5910-11996) | 181.63 (123.84-252.17) | 7298 (4986-10302) | 187.11 (127.47-264.52) | 0.11 (0.11-0.12) |  |
| Cuba | 14633 (9974-20551) | 134.7 (91.78-189.12) | 14416 (9861-20196) | 135.7 (92.95-190.58) | 0.04 (0.03-0.04) |  |
| Cyprus | 1277 (867-1789) | 164.91 (111.8-231.08) | 2156 (1465-3032) | 166.03 (112.26-233.18) | 0.04 (0.03-0.05) |  |
| Czech Republic | 18029 (12491-25101) | 180.27 (125.04-251.76) | 18545 (12872-25998) | 186.98 (131.14-261.56) | 0.11 (0.1-0.13) |  |
| C?te d'Ivoire | 21509 (14578-29995) | 164.11 (111.44-229.52) | 50068 (35040-70057) | 169.9 (118.67-236.85) | 0.11 (0.1-0.12) |  |
| Denmark | 8410 (5849-11672) | 172.43 (119.75-238.56) | 9669 (6538-13341) | 177.7 (119.67-245) | 0.09 (0.07-0.11) |  |
| Djibouti | 755 (515-1053) | 170.34 (117.31-237.63) | 2289 (1615-3245) | 174.75 (123.32-247.28) | 0.09 (0.08-0.1) |  |
| Dominica | 96 (66-135) | 129.6 (88.52-181.27) | 85 (59-122) | 130.21 (90.09-185.36) | 0.03 (0.03-0.04) |  |
| Dominican Republic | 9365 (6365-13104) | 125.05 (85.39-175.44) | 14058 (9607-19552) | 126.94 (86.83-176.56) | 0.06 (0.05-0.08) |  |
| Eastern Uruguay | 6041 (4097-8308) | 196.22 (133.09-270.16) | 6418 (4433-9111) | 198.63 (137.54-283.63) | 0.02 (0.01-0.03) |  |
| Ecuador | 13090 (9061-18572) | 125.29 (86.68-178.02) | 23310 (15653-32407) | 128.21 (86.16-178.39) | 0.08 (0.07-0.09) |  |
| El Salvador | 7583 (5418-10626) | 136.14 (97.08-190.46) | 9025 (6119-12601) | 139.51 (94.52-194.77) | 0.08 (0.07-0.09) |  |
| Equatorial Guinea | 717 (495-1013) | 156.57 (108.58-221.58) | 2807 (1915-4027) | 172.94 (118.24-247.22) | 0.39 (0.37-0.41) |  |
| Eritrea | 5944 (4111-8298) | 159.98 (111.67-222.59) | 11664 (8068-16478) | 165.67 (114.78-233.7) | 0.12 (0.12-0.12) |  |
| Estonia | 2642 (1842-3672) | 173.47 (120.88-240.94) | 2190 (1509-3016) | 181.01 (124.08-248.97) | 0.17 (0.16-0.18) |  |
| Eswatini | 1426 (961-1991) | 163.57 (110.53-228.31) | 2009 (1388-2809) | 164.42 (114.2-229.13) | 0.02 (0-0.05) |  |
| Ethiopia | 87689 (60603-123244) | 159.73 (110.49-224.44) | 193673 (133619-271797) | 167.14 (114.81-234.15) | 0.15 (0.15-0.16) |  |
| Federated States of Micronesia | 140 (96-197) | 127.47 (87.05-179.5) | 135 (91-190) | 128.3 (87.04-180.83) | 0.03 (0.02-0.04) |  |
| Fiji | 1021 (705-1423) | 128.47 (88.25-179.23) | 1206 (829-1731) | 128.41 (88.19-184.21) | 0 (0-0.01) |  |
| Finland | 7449 (5070-10523) | 155.35 (105.68-220.82) | 7801 (5346-10900) | 155.49 (106.66-218.29) | 0.04 (0.03-0.05) |  |
| French Republic | 90966 (61087-125628) | 163.81 (110.36-227.12) | 101268 (69594-141723) | 165.16 (112.74-232.25) | 0.04 (0.04-0.05) |  |
| Gabonese Republic | 1704 (1170-2351) | 164.68 (112.46-227.33) | 3180 (2157-4428) | 168.45 (114.23-234.18) | 0.08 (0.07-0.09) |  |
| Gambia | 1746 (1170-2444) | 166.83 (112.63-233.7) | 4252 (2959-5888) | 167.63 (116.77-232.56) | 0.02 (0.01-0.03) |  |
| Georgia | 9282 (6283-12973) | 169.88 (114.83-237.67) | 5821 (4080-8209) | 170.93 (119.38-241.98) | 0.03 (0.02-0.03) |  |
| Germany | 124845 (83870-175275) | 165.26 (110.99-232.31) | 130817 (90463-181611) | 168.38 (116.19-233.52) | 0.04 (0.03-0.05) |  |
| Ghana | 26328 (18381-36764) | 164.78 (114.39-229.53) | 60404 (40600-84566) | 167.65 (111.96-234.29) | 0.06 (0.05-0.06) |  |
| Grand Duchy of Luxembourg | 609 (415-842) | 166.47 (114-231.35) | 1039 (719-1435) | 169.42 (116.73-234.65) | 0.07 (0.06-0.07) |  |
| Great Britain and Northern Ireland | 70242 (48077-98631) | 129.01 (88.25-181.08) | 89120 (60590-125116) | 140.64 (95.42-198.12) | 0.33 (0.26-0.39) |  |
| Greenland | 108 (75-154) | 188.68 (131.23-268.56) | 105 (73-148) | 190.38 (131.27-267.88) | 0.04 (0.03-0.05) |  |
| Grenada | 114 (78-160) | 128.15 (87.4-178.48) | 133 (92-185) | 130.48 (90.1-182.78) | 0.07 (0.06-0.07) |  |
| Guam | 196 (133-274) | 138.95 (93.98-194.28) | 213 (143-300) | 136.78 (92.17-192.8) | -0.02 (-0.03--0.01) |  |
| Guatemala | 11906 (8220-16751) | 132.38 (91.51-186.71) | 21944 (15139-30974) | 135.37 (93.69-191.07) | 0.07 (0.07-0.08) |  |
| Guinea | 10247 (7152-14497) | 160.95 (112.67-227.52) | 23384 (15957-32676) | 163.2 (111.02-228.79) | 0.04 (0.04-0.05) |  |
| Guinea-Bissau | 1724 (1190-2448) | 158.03 (108.1-222.88) | 3587 (2472-5031) | 161.39 (112.08-225.97) | 0.08 (0.07-0.08) |  |
| Guyana | 1016 (699-1428) | 123.29 (85.06-172.56) | 956 (647-1332) | 123.2 (83.42-171.43) | 0.01 (0-0.02) |  |
| Haiti | 7874 (5531-11110) | 115.78 (80.81-162.68) | 15683 (10695-22195) | 116.54 (79.47-164.72) | 0.04 (0.04-0.05) |  |
| Hashemite Jordan | 5876 (4019-8403) | 148.57 (101.51-212.78) | 19176 (13069-26861) | 151.72 (103.31-212.25) | 0.09 (0.08-0.1) |  |
| Hellenic Republic | 16603 (11316-23540) | 166.16 (113.5-236.36) | 15183 (10277-21413) | 165.3 (111.55-233.25) | -0.02 (-0.03--0.01) |  |
| Honduras | 6853 (4782-9638) | 136.25 (95.33-190.85) | 14263 (9709-20193) | 136.25 (92.73-192.57) | 0.01 (0-0.01) |  |
| Hungary | 17661 (12196-24839) | 176.29 (121.33-248.94) | 16201 (11131-22380) | 182.54 (124.36-253.33) | 0.1 (0.1-0.11) |  |
| Iceland | 486 (336-687) | 193.35 (133.88-273.2) | 661 (458-917) | 196.91 (136.24-273.19) | 0.06 (0.05-0.07) |  |
| India | 1168136 (803220-1647739) | 130.13 (89.56-183.12) | 1887143 (1285675-2649048) | 131.9 (89.85-185.24) | 0.04 (0.03-0.05) |  |
| Indonesia | 229457 (157216-322857) | 118.56 (81.51-166.66) | 342925 (230158-484117) | 122.03 (81.91-172.03) | 0.12 (0.1-0.13) |  |
| Iran | 86572 (58947-120628) | 143.88 (97.8-200.55) | 126162 (85751-178256) | 147.78 (100.55-208.82) | 0.09 (0.07-0.1) |  |
| Iraq | 25205 (17101-35300) | 129.08 (88.12-179.25) | 56279 (38561-78842) | 132.02 (90.57-184.87) | 0.07 (0.06-0.08) |  |
| Ireland | 6924 (4824-9672) | 194.09 (135.22-271.28) | 9519 (6520-13219) | 200.9 (138.5-279.33) | 0.12 (0.1-0.15) |  |
| Israel | 7464 (5219-10361) | 149.74 (104.66-207.75) | 14104 (9652-19849) | 149.86 (102.63-211.17) | 0 (-0.01-0.02) |  |
| Italy | 99228 (67921-138375) | 183.79 (125.64-256.6) | 101036 (69889-141835) | 187.7 (129.59-263.77) | 0.09 (0.07-0.1) |  |
| Jamaica | 3186 (2161-4472) | 131.58 (89.32-183.92) | 3639 (2552-5065) | 131.78 (92.2-182.61) | 0.02 (0.01-0.03) |  |
| Japan | 336208 (234691-465749) | 274.86 (191.68-381.38) | 340640 (236444-476164) | 299.14 (207.18-420.04) | 0.28 (0.25-0.31) |  |
| Kazakhstan | 27661 (19048-39202) | 164.75 (113.78-233.49) | 32106 (22340-45639) | 168.55 (117.33-239.33) | 0.11 (0.09-0.14) |  |
| Kenya | 41838 (28594-58781) | 167.76 (114.84-235.12) | 89582 (61198-125927) | 169.7 (116.15-238.2) | 0.04 (0.02-0.05) |  |
| Kiribati | 96 (66-134) | 121.01 (82.86-168.89) | 153 (106-215) | 121.3 (83.7-170.13) | 0.01 (0-0.01) |  |
| Korea | 27284 (18771-38170) | 129.84 (89.3-181.86) | 138184 (95735-191645) | 133.93 (93.45-187.12) | 0.18 (-0.8-1.18) |  |
| Kuwait | 2816 (1945-3993) | 157.94 (109.09-223.35) | 7266 (4864-10244) | 155.04 (104.34-218.73) | -0.06 (-0.09--0.03) |  |
| Kyrgyz Republic | 7570 (5281-10576) | 163.45 (114.36-229.62) | 11780 (7913-16664) | 167.25 (112.32-236.03) | 0.08 (0.07-0.09) |  |
| Lao Republic | 5343 (3722-7540) | 120.6 (84.4-170.03) | 9593 (6535-13525) | 126.12 (85.89-177.63) | 0.16 (0.15-0.17) |  |
| Latvia | 4421 (2961-6263) | 172.17 (115.7-243.86) | 3015 (2093-4222) | 176.87 (122.61-248.05) | 0.09 (0.08-0.1) |  |
| Lebanese Republic | 4856 (3380-6774) | 157.88 (110.17-220.26) | 8901 (5936-12446) | 162.48 (108.39-226.86) | 0.11 (0.1-0.12) |  |
| Lesotho | 2608 (1784-3641) | 159.27 (108.71-223.08) | 3175 (2193-4366) | 161.21 (111.17-222.16) | 0.04 (0.03-0.05) |  |
| Liberia | 4225 (2904-5978) | 161.19 (110.92-226.89) | 9667 (6559-13462) | 167.44 (114.24-232.26) | 0.18 (0.17-0.2) |  |
| Libya | 6383 (4458-9120) | 144.81 (101.84-205.75) | 9959 (6741-14056) | 143.55 (97.17-202.76) | 0.01 (-0.01-0.02) |  |
| Lithuania | 6256 (4243-8800) | 174.12 (117.94-245.15) | 4411 (3032-6136) | 177.3 (121.46-247.66) | 0.05 (0.04-0.06) |  |
| Madagascar | 20878 (14562-29162) | 164.06 (114.2-228.71) | 50561 (35257-70976) | 166.47 (116.2-231.55) | 0.06 (0.06-0.07) |  |
| Malawi | 17058 (11800-23896) | 161.5 (111.96-225.99) | 34398 (23413-48405) | 165.56 (112.85-232.77) | 0.09 (0.08-0.1) |  |
| Malaysia | 24570 (16656-34695) | 133.66 (90.55-189.29) | 44217 (30560-62353) | 138.15 (95.52-194.87) | 0.12 (0.1-0.13) |  |
| Maldives | 305 (204-436) | 130.71 (87.6-187.18) | 772 (515-1086) | 144.37 (97.15-203.37) | 0.35 (0.32-0.38) |  |
| Mali | 14937 (10284-20997) | 160.99 (110.82-225.6) | 42570 (29089-59967) | 164.82 (113.2-232.36) | 0.09 (0.08-0.09) |  |
| Malta | 608 (421-852) | 166.67 (115.25-233.31) | 686 (462-963) | 168.93 (113.79-236.82) | 0.05 (0.05-0.06) |  |
| Marshall Islands | 62 (42-85) | 127.12 (86.94-175) | 74 (51-104) | 126.64 (88.61-179.34) | -0.01 (-0.01-0) |  |
| Mauritania | 3613 (2484-5146) | 165.55 (114.05-235.2) | 7922 (5419-11241) | 171.58 (116.97-243.71) | 0.12 (0.11-0.12) |  |
| Mauritius | 1511 (1032-2125) | 134.11 (91.67-188.33) | 1674 (1138-2339) | 136.33 (92.47-190.59) | 0.04 (0.03-0.05) |  |
| Mexican States | 125303 (86065-174717) | 139.39 (95.91-194.01) | 183348 (123704-257839) | 142.17 (95.87-199.9) | 0.05 (0.05-0.06) |  |
| Moldova | 7487 (5081-10481) | 167.95 (114.05-235.29) | 5836 (3954-8252) | 172.53 (116.8-244.28) | 0.09 (0.08-0.09) |  |
| Monaco | 46 (31-64) | 168.42 (115.62-235.97) | 57 (39-80) | 167.26 (115.2-235.09) | -0.02 (-0.04--0.01) |  |
| Mongolia | 3625 (2489-5078) | 158.88 (108.56-221.99) | 5523 (3840-7824) | 161.24 (112-227.83) | 0.09 (0.08-0.11) |  |
| Montenegro | 1134 (779-1615) | 181.18 (124.69-258.08) | 1087 (760-1537) | 183.45 (127.73-260.1) | 0.07 (0.06-0.08) |  |
| Morocco | 36990 (24926-51673) | 139.55 (94-195.92) | 52698 (36207-73556) | 141.17 (96.96-196.95) | 0.05 (0.04-0.05) |  |
| Mozambique | 22813 (15366-31712) | 158.94 (106.87-221.44) | 54316 (37638-75615) | 161.78 (111.9-225.7) | 0.05 (0.05-0.05) |  |
| Myanmar | 51317 (35234-72162) | 121.35 (83.21-170.56) | 71764 (48698-101559) | 125.81 (85.37-178) | 0.13 (0.11-0.14) |  |
| Namibia | 2468 (1712-3435) | 165.05 (114.95-229.11) | 4279 (2930-6026) | 168.77 (115.89-237.5) | 0.08 (0.07-0.09) |  |
| Nauru | 14 (10-19) | 128.22 (89.4-178.95) | 15 (10-21) | 126.06 (85.97-177.78) | -0.05 (-0.07--0.03) |  |
| Nepal | 23950 (16558-33224) | 115.57 (79.82-161) | 37255 (25067-52394) | 116.88 (78.68-164.38) | 0.03 (0.02-0.04) |  |
| Netherlands | 24409 (16923-34472) | 168.69 (116.76-238) | 27115 (18999-38138) | 169.04 (117.42-236.86) | 0.02 (0.01-0.02) |  |
| New Zealand | 7159 (4995-10036) | 212.03 (148.14-297.71) | 10855 (7492-15250) | 218.25 (150.32-306.67) | 0.09 (0.09-0.1) |  |
| Nicaragua | 5731 (3910-8071) | 138.49 (94.23-195.76) | 9636 (6517-13458) | 141.5 (95.94-197.8) | 0.08 (0.08-0.09) |  |
| Niger | 14043 (9754-19755) | 161.91 (113.11-226.18) | 44504 (30378-61385) | 164.23 (112.49-227.73) | 0.06 (0.06-0.07) |  |
| Nigeria | 155454 (106316-218498) | 163.91 (112.37-230.6) | 405272 (277499-571892) | 164.61 (112.88-231.74) | 0.02 (0.01-0.03) |  |
| Niue | 3 (2-4) | 130.8 (90.17-184.49) | 2 (1-3) | 131.6 (89.9-184) | 0.02 (0.02-0.03) |  |
| North Macedonia | 3510 (2375-4932) | 175.36 (118.58-246.43) | 3758 (2555-5260) | 179.62 (123.06-251.72) | 0.12 (0.1-0.13) |  |
| Northern Mariana Islands | 64 (44-91) | 138.11 (94.94-193.87) | 67 (46-93) | 138.07 (94.93-193.01) | -0.01 (-0.03-0.01) |  |
| Norway | 6123 (4208-8619) | 151.85 (104.27-213.74) | 7748 (5354-10925) | 151.87 (104.72-214.36) | 0.05 (0.04-0.07) |  |
| Pakistan | 138275 (94026-191479) | 117.4 (79.95-162.78) | 290211 (200628-405965) | 117.52 (81.33-164.39) | 0 (0-0.01) |  |
| Palau | 21 (14-29) | 132.04 (90.76-187.69) | 24 (16-34) | 136.08 (91.98-191.81) | 0.09 (0.06-0.11) |  |
| Palestine | 3126 (2133-4413) | 143.41 (98.3-201.37) | 7797 (5338-10934) | 145.87 (99.88-204.48) | 0.05 (0.04-0.06) |  |
| Panama | 3546 (2394-4955) | 143.85 (97.21-201.65) | 6257 (4321-8866) | 146.33 (101.11-207.37) | 0.04 (0.04-0.05) |  |
| Papua New Guinea | 5437 (3703-7659) | 125.41 (85.74-176.98) | 13726 (9412-19165) | 125.38 (86.06-174.85) | 0 (-0.01-0) |  |
| Paraguay | 4913 (3367-6862) | 116.19 (79.85-161.99) | 8533 (5741-11754) | 117.43 (79.01-161.81) | 0.04 (0.03-0.05) |  |
| Peru | 28168 (19267-39385) | 124.73 (85.55-174.62) | 47312 (32150-66419) | 130.1 (88.38-182.65) | 0.15 (0.14-0.15) |  |
| Philippines | 84268 (56895-118949) | 127.21 (86.12-179.7) | 149400 (101109-210256) | 128.98 (87.33-181.6) | 0.04 (0.03-0.06) |  |
| Poland | 65857 (45233-91745) | 174.92 (120.23-243.57) | 64669 (44726-89944) | 180.86 (125.21-251.84) | 0.12 (0.11-0.13) |  |
| Portuguese Republic | 15511 (10693-21735) | 158.52 (109.28-222.36) | 15431 (10485-21798) | 161.47 (109.7-227.91) | 0.06 (0.05-0.07) |  |
| Puerto Rico | 4887 (3362-6930) | 135.34 (93.08-191.68) | 4125 (2833-5707) | 136.91 (93.43-190.84) | 0.06 (0.05-0.07) |  |
| Qatar | 817 (567-1148) | 174.06 (120.76-244.95) | 5693 (3911-7937) | 180.64 (123.63-251.18) | 0.24 (0.2-0.28) |  |
| Romania | 39231 (26644-55431) | 170.53 (115.91-241.1) | 31067 (21342-43185) | 176.54 (121.66-244.57) | 0.14 (0.13-0.14) |  |
| Russian Federation | 249540 (170445-349118) | 168.64 (114.93-235.92) | 237330 (163785-332074) | 173.88 (119.72-243.97) | 0.12 (0.1-0.14) |  |
| Rwanda | 12502 (8564-17492) | 160.62 (109.41-224.6) | 23353 (15913-32436) | 166.52 (113.25-232.41) | 0.18 (0.15-0.21) |  |
| Saint Kitts and Nevis | 54 (37-75) | 127.89 (88.14-178.04) | 75 (52-105) | 130.06 (89.45-182.3) | 0.06 (0.05-0.08) |  |
| Saint Lucia | 183 (123-257) | 129.04 (87.25-181.57) | 225 (153-315) | 131.25 (88.71-184.49) | 0.07 (0.06-0.07) |  |
| Saint Vincent andGrenadines | 146 (99-204) | 128.7 (86.49-179.34) | 145 (99-203) | 129.66 (88.62-182.44) | 0.03 (0.02-0.04) |  |
| Samoa | 233 (160-325) | 130.82 (89.7-182.84) | 288 (193-407) | 130.94 (87.8-184.5) | 0 (0-0.01) |  |
| San Marino | 38 (27-54) | 168.39 (116.21-235.15) | 50 (35-70) | 167.46 (114.87-234) | -0.01 (-0.02--0.01) |  |
| Sao Tome and Principe | 216 (152-306) | 168.41 (118.05-237) | 392 (261-559) | 173.56 (116.03-247.11) | 0.11 (0.1-0.13) |  |
| Saudi Arabia | 24723 (17116-34818) | 148.34 (103.11-208.99) | 59138 (40825-84470) | 152.76 (104.88-218.5) | 0.11 (0.1-0.13) |  |
| Senegal | 13329 (9205-18761) | 163.47 (112.77-228.78) | 28088 (19509-39427) | 168.06 (117.53-235.9) | 0.1 (0.09-0.1) |  |
| Serbia | 16648 (11479-23413) | 174.92 (120.61-246.06) | 15254 (10484-21344) | 182.15 (125.66-254.14) | 0.15 (0.14-0.16) |  |
| Seychelles | 99 (69-138) | 133.71 (93.18-186.17) | 145 (99-204) | 138.15 (94.31-194.64) | 0.12 (0.11-0.13) |  |
| Sierra Leone | 7143 (4868-9975) | 162.27 (111.17-226.24) | 15568 (10764-22121) | 166.03 (115.11-235.06) | 0.1 (0.08-0.12) |  |
| Singapore | 8347 (5735-11712) | 269.77 (184.86-377.53) | 15599 (10775-21490) | 280.95 (193.86-388.04) | 0.14 (0.13-0.16) |  |
| Slovak Republic | 9236 (6303-12725) | 176.8 (120.6-244.09) | 9308 (6379-13011) | 181.42 (123.93-254) | 0.1 (0.09-0.1) |  |
| Slovenia | 3526 (2421-4936) | 182.77 (125.86-256.48) | 3673 (2526-5180) | 190.86 (129.3-268.88) | 0.17 (0.16-0.17) |  |
| Solomon Islands | 456 (307-638) | 126.77 (84.7-177.2) | 903 (618-1270) | 126.07 (86.22-176.83) | -0.01 (-0.02--0.01) |  |
| Somalia | 14120 (9692-19971) | 162.88 (111.7-230.04) | 38420 (26189-54091) | 162.2 (111.26-227.45) | 0.01 (0-0.02) |  |
| South Africa | 64565 (44800-91297) | 166.25 (115.56-234.24) | 97378 (66684-136767) | 169.08 (115.77-237.68) | 0.08 (0.06-0.1) |  |
| South Sudan | 10447 (7179-14861) | 167.06 (114.72-236.25) | 17122 (11851-23751) | 165.48 (115.33-231.18) | -0.01 (-0.02-0) |  |
| Spain | 69217 (48165-97866) | 184.92 (128.76-261.31) | 80710 (56601-110405) | 192.81 (134.47-265.36) | 0.15 (0.12-0.18) |  |
| Sri Lanka | 24102 (16701-33763) | 136.77 (94.68-191.17) | 30838 (21382-43324) | 140.8 (97.36-197.86) | 0.09 (0.08-0.11) |  |
| Sudan | 28650 (19588-41205) | 134.83 (92.21-192.36) | 62565 (42927-87689) | 137.52 (94.41-193.47) | 0.07 (0.06-0.07) |  |
| Sultanate of Oman | 3187 (2159-4453) | 152.58 (103.12-212.19) | 7672 (5137-10759) | 156.13 (104.88-218.73) | 0.07 (0.05-0.09) |  |
| Suriname | 505 (348-707) | 126.17 (86.97-176.67) | 719 (492-1003) | 125.54 (85.82-175.06) | 0 (-0.01-0.01) |  |
| Sweden | 14970 (10197-21127) | 185.32 (126.06-262.14) | 18384 (12565-25850) | 191.32 (130.85-270.03) | 0.13 (0.11-0.15) |  |
| Swiss Confederation | 10972 (7484-15494) | 167.26 (114.4-236.41) | 14041 (9780-19662) | 169.35 (117.54-235.93) | 0.05 (0.04-0.05) |  |
| Syrian Arab Republic | 19203 (13129-26783) | 142.66 (98.21-198.62) | 19995 (13733-28294) | 141.13 (96.65-200.13) | -0.02 (-0.07-0.02) |  |
| Taiwan (Province of China) | 33911 (23794-48007) | 164.17 (115.16-231.9) | 38754 (26701-54538) | 172.24 (119.39-241.67) | 0.16 (0.13-0.19) |  |
| Tajikistan | 9178 (6341-13065) | 162.37 (111.57-230.33) | 17174 (11706-24280) | 162.94 (111.22-230.08) | 0.02 (0.01-0.03) |  |
| Tanzania | 45026 (31290-62592) | 162.73 (113.86-225.11) | 103772 (71114-146584) | 167.6 (115.09-236.77) | 0.11 (0.1-0.12) |  |
| Thailand | 76503 (53266-109395) | 130.98 (90.97-187.71) | 86247 (59117-122373) | 135.98 (93.04-192.68) | 0.13 (0.12-0.14) |  |
| Timor-Leste | 1037 (696-1448) | 124.82 (84.39-174.2) | 1842 (1293-2602) | 126.86 (89.23-179.55) | 0.08 (0.06-0.1) |  |
| Togolese Republic | 6382 (4326-9032) | 162.05 (109.61-227.96) | 14682 (10186-20530) | 165.83 (115.38-230.24) | 0.08 (0.07-0.09) |  |
| Tokelau | 2 (1-3) | 131.54 (90.27-186.12) | 2 (1-3) | 134.14 (92.16-189.26) | 0.08 (0.07-0.09) |  |
| Tonga | 135 (93-189) | 130.64 (90.09-182.88) | 142 (98-202) | 130.72 (89.46-185.48) | -0.01 (-0.02-0) |  |
| Trinidad and Tobago | 1609 (1098-2240) | 129.8 (88.61-180.7) | 1756 (1199-2439) | 130.73 (89.32-181.38) | 0.04 (0.03-0.04) |  |
| Tunisia | 12613 (8634-17545) | 145.71 (99.55-202.6) | 17144 (11666-24099) | 146.59 (99.65-206.83) | 0.04 (0.03-0.04) |  |
| Turkey | 85264 (58627-119175) | 143.04 (98.49-200.64) | 121955 (82458-170704) | 147.64 (99.71-206.95) | 0.11 (0.1-0.12) |  |
| Turkmenistan | 6330 (4395-8992) | 162.48 (112.12-229.49) | 8750 (5872-12284) | 166.46 (111.7-233.72) | 0.09 (0.08-0.09) |  |
| Tuvalu | 12 (8-17) | 122.88 (84.59-171.24) | 16 (11-23) | 128.97 (87.06-180.95) | 0.16 (0.15-0.17) |  |
| Uganda | 30437 (21032-43111) | 163.12 (113.52-229.53) | 78396 (54902-109955) | 167.89 (118.19-234.37) | 0.11 (0.1-0.12) |  |
| Ukraine | 87026 (59098-122686) | 170.9 (115.91-240.37) | 68852 (47411-95561) | 172.66 (119.13-240.27) | 0.05 (0.04-0.06) |  |
| Union ofComoros | 822 (572-1169) | 165.93 (114.92-234.83) | 1308 (913-1831) | 170.12 (118.73-238.08) | 0.1 (0.09-0.11) |  |
| Uzbekistan | 35925 (24469-50449) | 163.65 (111.71-230.35) | 57588 (39254-81168) | 165.12 (112.32-232.69) | 0.06 (0.05-0.06) |  |
| Vanuatu | 205 (141-284) | 127.42 (87.69-176.7) | 411 (280-587) | 125.96 (85.86-178.95) | -0.03 (-0.03--0.02) |  |
| Venezuela | 27679 (19114-38459) | 140.6 (97.22-194.84) | 37364 (25892-52416) | 142.05 (98.52-199.82) | 0.06 (0.04-0.07) |  |
| Viet Nam | 92039 (63221-129977) | 129.2 (88.78-182.5) | 137543 (95424-192945) | 137.33 (95.21-193.05) | 0.19 (0.18-0.2) |  |
| Virgin Islands | 142 (95-202) | 131.88 (88.66-187.25) | 104 (71-145) | 132.86 (91.06-185.55) | 0.03 (0.02-0.04) |  |
| Yemen | 19751 (13801-28035) | 134.38 (93.77-190.84) | 47867 (33123-68346) | 134.83 (93.42-191.54) | 0.02 (0.01-0.03) |  |
| Zambia | 13876 (9592-19187) | 162.3 (112.51-224.93) | 34642 (23757-48771) | 166.44 (114.64-234.31) | 0.11 (0.09-0.13) |  |
| Zimbabwe | 18444 (12568-25882) | 166.62 (114.4-234.43) | 27087 (18603-38384) | 162.99 (111.9-229.65) | -0.09 (-0.11--0.08) |  |

UI: uncertainty interval; CI: Confidence Interval; DALYs: disability-adjusted life years; EAPC: Estimated Annual Percentage Change; ASD: autism spectrum disorder; SDI: socio-demographic index

## **Table S3.** The predicted results in the autism spectrum disorders-related numbers and age-standardized rates of prevalence and DALYs by sex globally from 2022 to 2046 of the APC model.

| year | sex | Age-standardized prevalence rate | Number of prevalence cases | Age-standardized DALYs rate | Number of DALYs cases |
| --- | --- | --- | --- | --- | --- |
| 2022 | Female | 510.87 | 20146138.15 | 95.08 | 3735227.129 |
| 2023 | Female | 510.79 | 20313369.26 | 95.06 | 3764659.238 |
| 2024 | Female | 510.72 | 20474904.8 | 95.03 | 3792979.461 |
| 2025 | Female | 511.1 | 20646213.57 | 95.1 | 3823152.559 |
| 2026 | Female | 511.49 | 20812254.26 | 95.16 | 3852312.751 |
| 2027 | Female | 511.88 | 20973771.55 | 95.22 | 3880612.065 |
| 2028 | Female | 512.26 | 21130067.37 | 95.29 | 3907909.048 |
| 2029 | Female | 512.65 | 21280188.71 | 95.35 | 3934015.415 |
| 2030 | Female | 512.9 | 21419423.92 | 95.39 | 3957941.978 |
| 2031 | Female | 513.16 | 21553439.99 | 95.42 | 3980881.678 |
| 2032 | Female | 513.42 | 21682833.27 | 95.46 | 4002952.848 |
| 2033 | Female | 513.67 | 21807299.89 | 95.5 | 4024111.653 |
| 2034 | Female | 513.93 | 21925686.69 | 95.54 | 4044116.485 |
| 2035 | Female | 514.04 | 22032720.56 | 95.54 | 4061837.908 |
| 2036 | Female | 514.15 | 22134702.84 | 95.55 | 4078613.419 |
| 2037 | Female | 514.26 | 22232350.24 | 95.56 | 4094583.732 |
| 2038 | Female | 514.37 | 22324788.66 | 95.57 | 4109578.84 |
| 2039 | Female | 514.48 | 22411299.72 | 95.57 | 4123457.668 |
| 2040 | Female | 514.62 | 22495554.45 | 95.59 | 4137085.451 |
| 2041 | Female | 514.76 | 22575003.88 | 95.61 | 4149824.849 |
| 2042 | Female | 514.9 | 22650248.69 | 95.63 | 4161788.722 |
| 2043 | Female | 515.05 | 22720628.66 | 95.64 | 4172859.73 |
| 2044 | Female | 515.19 | 22785103.02 | 95.66 | 4182826.416 |
| 2045 | Female | 515.33 | 22844126.05 | 95.68 | 4191778.293 |
| 2046 | Female | 515.47 | 22898167.02 | 95.7 | 4199807.622 |
| 2022 | Male | 1071.85 | 43192210.5 | 201.25 | 8097824.64 |
| 2023 | Male | 1071.22 | 43550814.51 | 201.1 | 8161695.558 |
| 2024 | Male | 1070.59 | 43898307.54 | 200.95 | 8223342.621 |
| 2025 | Male | 1070.87 | 44264885.62 | 201 | 8289283.199 |
| 2026 | Male | 1071.14 | 44620538.93 | 201.04 | 8353123.163 |
| 2027 | Male | 1071.41 | 44966302.89 | 201.08 | 8415094.784 |
| 2028 | Male | 1071.69 | 45301194.41 | 201.12 | 8474968.823 |
| 2029 | Male | 1071.96 | 45623942.85 | 201.17 | 8532462.388 |
| 2030 | Male | 1071.9 | 45919254.93 | 201.15 | 8585097.309 |
| 2031 | Male | 1071.85 | 46203564.56 | 201.14 | 8635655.981 |
| 2032 | Male | 1071.79 | 46477629.1 | 201.13 | 8684310.246 |
| 2033 | Male | 1071.73 | 46741313.01 | 201.12 | 8731019.011 |
| 2034 | Male | 1071.68 | 46993127.68 | 201.11 | 8775442.646 |
| 2035 | Male | 1071.28 | 47216965.25 | 201.04 | 8814949.327 |
| 2036 | Male | 1070.88 | 47430326.35 | 200.97 | 8852511.153 |
| 2037 | Male | 1070.48 | 47634240.78 | 200.9 | 8888347.253 |
| 2038 | Male | 1070.08 | 47826919.79 | 200.83 | 8922102.532 |
| 2039 | Male | 1069.69 | 48007802.13 | 200.76 | 8953620.299 |
| 2040 | Male | 1069.48 | 48187929.81 | 200.72 | 8984911.516 |
| 2041 | Male | 1069.28 | 48357921.91 | 200.69 | 9014334.731 |
| 2042 | Male | 1069.08 | 48518448.35 | 200.65 | 9042036.33 |
| 2043 | Male | 1068.87 | 48668525.13 | 200.62 | 9067812.191 |
| 2044 | Male | 1068.67 | 48806521.75 | 200.59 | 9091312.707 |
| 2045 | Male | 1068.47 | 48933014.67 | 200.55 | 9112668.308 |
| 2046 | Male | 1068.26 | 49048492.63 | 200.52 | 9131991.983 |

DALYs: disability-adjusted life years;

## **Table S4.** The predicted results in the autism spectrum disorders-related numbers and age-standardized rates of prevalence and DALYs by sex globally from 2022 to 2046 of the ARIMA model.

| year | sex | Age-standardized prevalence rate | Number of prevalence cases | Age-standardized DALYs rate | Number of DALYs cases |
| --- | --- | --- | --- | --- | --- |
| 2022 | Male | 1064.181562 | 42409207.41 | 199.7309088 | 7949376.882 |
| 2023 | Male | 1063.852969 | 42677651.8 | 199.7471233 | 8006446.598 |
| 2024 | Male | 1063.880208 | 42965866.44 | 199.8421477 | 8071358.004 |
| 2025 | Male | 1064.235683 | 43289409.13 | 199.950213 | 8144349.073 |
| 2026 | Male | 1064.80414 | 43653577.93 | 200.0582782 | 8223738.144 |
| 2027 | Male | 1065.461125 | 44055630.05 | 200.1663435 | 8307057.959 |
| 2028 | Male | 1066.115855 | 44487617.19 | 200.2744088 | 8391921.861 |
| 2029 | Male | 1066.722706 | 44939202.66 | 200.3824741 | 8476531.569 |
| 2030 | Male | 1067.27316 | 45400017.65 | 200.4905394 | 8559852.542 |
| 2031 | Male | 1067.780253 | 45861319.63 | 200.5986047 | 8641547.841 |
| 2032 | Male | 1068.264003 | 46316897.21 | 200.7066699 | 8721780.296 |
| 2033 | Male | 1068.741818 | 46763302.62 | 200.8147352 | 8800979.419 |
| 2034 | Male | 1069.224409 | 47199575.32 | 200.9228005 | 8879639.582 |
| 2035 | Male | 1069.715768 | 47626652.59 | 201.0308658 | 8958182.805 |
| 2036 | Male | 1070.21523 | 48046654.23 | 201.1389311 | 9036891.789 |
| 2037 | Male | 1070.719958 | 48462192.64 | 201.2469964 | 9115900.737 |
| 2038 | Male | 1071.226881 | 48875810.44 | 201.3550616 | 9195223.345 |
| 2039 | Male | 1071.733756 | 49289596.97 | 201.4631269 | 9274797.037 |
| 2040 | Male | 1072.239453 | 49704991.34 | 201.5711922 | 9354527.062 |
| 2041 | Male | 1072.743758 | 50122747.57 | 201.6792575 | 9434320.55 |
| 2042 | Male | 1073.246993 | 50543019.15 | 201.7873228 | 9514106.763 |
| 2043 | Male | 1073.749649 | 50965513.79 | 201.8953881 | 9593844.387 |
| 2044 | Male | 1074.252158 | 51389672.93 | 202.0034533 | 9673519.321 |
| 2045 | Male | 1074.754784 | 51814839.98 | 202.1115186 | 9753137.167 |
| 2046 | Male | 1075.257626 | 52240394.02 | 202.2195839 | 9832714.232 |
| 2047 | Male | 1075.760668 | 52665837.85 | 202.3276492 | 9912269.636 |
| 2048 | Male | 1076.26384 | 53090840.15 | 202.4357145 | 9991819.885 |
| 2049 | Male | 1076.767066 | 53515238.66 | 202.5437798 | 10071376.17 |
| 2050 | Male | 1077.270292 | 53939015.57 | 202.651845 | 10150943.91 |
| 2022 | Female | 508.6621476 | 19857522.24 | 94.50999128 | 3675192.907 |
| 2023 | Female | 509.0639664 | 20016974.78 | 94.56935894 | 3702248.458 |
| 2024 | Female | 509.2803854 | 20171070.07 | 94.61344333 | 3728752.96 |
| 2025 | Female | 509.3266361 | 20321752.15 | 94.65326004 | 3754933.565 |
| 2026 | Female | 509.2301361 | 20470259.63 | 94.67528087 | 3780923.791 |
| 2027 | Female | 509.0258155 | 20617381.62 | 94.69204272 | 3806802.116 |
| 2028 | Female | 508.7516469 | 20763620.89 | 94.69236554 | 3832614.668 |
| 2029 | Female | 508.4447334 | 20909297.77 | 94.68964376 | 3858388.559 |
| 2030 | Female | 508.1381986 | 21054616.33 | 94.67421685 | 3884139.726 |
| 2031 | Female | 507.8590016 | 21199706.61 | 94.65952015 | 3909877.537 |
| 2032 | Female | 507.6267016 | 21344651.44 | 94.63657895 | 3935607.498 |
| 2033 | Female | 507.453102 | 21489503.61 | 94.61816699 | 3961332.843 |
| 2034 | Female | 507.3426458 | 21634296.74 | 94.59536993 | 3987055.477 |
| 2035 | Female | 507.2933858 | 21779052.25 | 94.57986484 | 4012776.516 |
| 2036 | Female | 507.298339 | 21923783.8 | 94.56247324 | 4038496.618 |
| 2037 | Female | 507.3470346 | 22068500.08 | 94.55364359 | 4064216.169 |
| 2038 | Female | 507.4270827 | 22213206.63 | 94.5438951 | 4089935.397 |
| 2039 | Female | 507.5256219 | 22357906.98 | 94.54256389 | 4115654.434 |
| 2040 | Female | 507.630541 | 22502603.39 | 94.54003052 | 4141373.36 |
| 2041 | Female | 507.7314075 | 22647297.28 | 94.54478051 | 4167092.219 |
| 2042 | Female | 507.8200774 | 22791989.56 | 94.54731074 | 4192811.04 |
| 2043 | Female | 507.8909896 | 22936680.83 | 94.55555441 | 4218529.839 |
| 2044 | Female | 507.9411778 | 23081371.44 | 94.56037123 | 4244248.624 |
| 2045 | Female | 507.9700523 | 23226061.64 | 94.56939224 | 4269967.401 |
| 2046 | Female | 507.9790119 | 23370751.58 | 94.57401192 | 4295686.173 |

DALYs: disability-adjusted life years;

## **Table S5.** The predicted results in the autism spectrum disorders-related numbers and age-standardized rates of prevalence and DALYs by sex globally from 2022 to 2046 of the ES model

| year | sex | Age-standardized prevalence rate | Number of prevalence cases | Age-standardized DALYs rate | Number of DALYs cases |
| --- | --- | --- | --- | --- | --- |
| 2022 | Male | 1064.448469 | 42420668.3 | 199.6928123 | 7946470.099 |
| 2023 | Male | 1064.217419 | 42678775.02 | 199.5939888 | 7991138.366 |
| 2024 | Male | 1064.009475 | 42911071.07 | 199.5050477 | 8031339.806 |
| 2025 | Male | 1063.822325 | 43120137.52 | 199.4250007 | 8067521.102 |
| 2026 | Male | 1063.65389 | 43308297.31 | 199.3529585 | 8100084.269 |
| 2027 | Male | 1063.502299 | 43477641.13 | 199.2881204 | 8129391.119 |
| 2028 | Male | 1063.365867 | 43630050.57 | 199.2297661 | 8155767.284 |
| 2029 | Male | 1063.243078 | 43767219.07 | 199.1772473 | 8179505.832 |
| 2030 | Male | 1063.132568 | 43890670.71 | 199.1299804 | 8200870.526 |
| 2031 | Male | 1063.033109 | 44001777.19 | 199.0874401 | 8220098.75 |
| 2032 | Male | 1062.943596 | 44101773.02 | 199.0491539 | 8237404.152 |
| 2033 | Male | 1062.863034 | 44191769.27 | 199.0146963 | 8252979.013 |
| 2034 | Male | 1062.790528 | 44272765.89 | 198.9836844 | 8266996.389 |
| 2035 | Male | 1062.725273 | 44345662.86 | 198.9557738 | 8279612.027 |
| 2036 | Male | 1062.666543 | 44411270.12 | 198.9306542 | 8290966.101 |
| 2037 | Male | 1062.613687 | 44470316.66 | 198.9080466 | 8301184.768 |
| 2038 | Male | 1062.566116 | 44523458.54 | 198.8876997 | 8310381.568 |
| 2039 | Male | 1062.523302 | 44571286.24 | 198.8693875 | 8318658.688 |
| 2040 | Male | 1062.484769 | 44614331.17 | 198.8529065 | 8326108.096 |
| 2041 | Male | 1062.45009 | 44653071.6 | 198.8380737 | 8332812.563 |
| 2042 | Male | 1062.418879 | 44687937.99 | 198.8247241 | 8338846.584 |
| 2043 | Male | 1062.390789 | 44719317.74 | 198.8127095 | 8344277.202 |
| 2044 | Male | 1062.365507 | 44747559.52 | 198.8018963 | 8349164.759 |
| 2045 | Male | 1062.342754 | 44772977.12 | 198.7921645 | 8353563.56 |
| 2046 | Male | 1062.322277 | 44795852.96 | 198.7834058 | 8357522.48 |
| 2047 | Male | 1062.303847 | 44816441.21 | 198.775523 | 8361085.509 |
| 2048 | Male | 1062.28726 | 44834970.64 | 198.7684285 | 8364292.235 |
| 2049 | Male | 1062.272331 | 44851647.13 | 198.7620434 | 8367178.288 |
| 2050 | Male | 1062.258896 | 44866655.97 | 198.7562969 | 8369775.736 |
| 2022 | Female | 508.7498038 | 19852616.3 | 94.50849729 | 3673829.016 |
| 2023 | Female | 509.3559553 | 19999275.91 | 94.55958673 | 3697795.316 |
| 2024 | Female | 509.9014917 | 20131269.56 | 94.60556722 | 3719364.986 |
| 2025 | Female | 510.3924745 | 20250063.84 | 94.64694966 | 3738777.689 |
| 2026 | Female | 510.8343589 | 20356978.7 | 94.68419386 | 3756249.122 |
| 2027 | Female | 511.232055 | 20453202.07 | 94.71771364 | 3771973.411 |
| 2028 | Female | 511.5899814 | 20539803.1 | 94.74788144 | 3786125.272 |
| 2029 | Female | 511.9121152 | 20617744.03 | 94.77503246 | 3798861.946 |
| 2030 | Female | 512.2020356 | 20687890.86 | 94.79946838 | 3810324.953 |
| 2031 | Female | 512.4629639 | 20751023.02 | 94.82146071 | 3820641.659 |
| 2032 | Female | 512.6977995 | 20807841.95 | 94.8412538 | 3829926.695 |
| 2033 | Female | 512.9091514 | 20858979 | 94.85906759 | 3838283.227 |
| 2034 | Female | 513.0993682 | 20905002.34 | 94.87509999 | 3845804.106 |
| 2035 | Female | 513.2705633 | 20946423.34 | 94.88952916 | 3852572.897 |
| 2036 | Female | 513.4246389 | 20983702.25 | 94.90251541 | 3858664.809 |
| 2037 | Female | 513.5633069 | 21017253.26 | 94.91420303 | 3864147.53 |
| 2038 | Female | 513.6881082 | 21047449.17 | 94.9247219 | 3869081.978 |
| 2039 | Female | 513.8004293 | 21074625.5 | 94.93418887 | 3873522.982 |
| 2040 | Female | 513.9015183 | 21099084.19 | 94.94270915 | 3877519.885 |
| 2041 | Female | 513.9924983 | 21121097.01 | 94.9503774 | 3881117.098 |
| 2042 | Female | 514.0743804 | 21140908.54 | 94.95727882 | 3884354.59 |
| 2043 | Female | 514.1480743 | 21158738.93 | 94.96349011 | 3887268.333 |
| 2044 | Female | 514.2143988 | 21174786.28 | 94.96908026 | 3889890.701 |
| 2045 | Female | 514.2740908 | 21189228.89 | 94.9741114 | 3892250.833 |
| 2046 | Female | 514.3278137 | 21202227.24 | 94.97863943 | 3894374.951 |

DALYs: disability-adjusted life years;

# **Figure legend**

## **Figure S1.** Numbers and age-standardized rates of autism spectrum disorders-related prevalence and DALYs for both sexes in 2021. Abbreviations: DALYs, disability-adjusted life years.


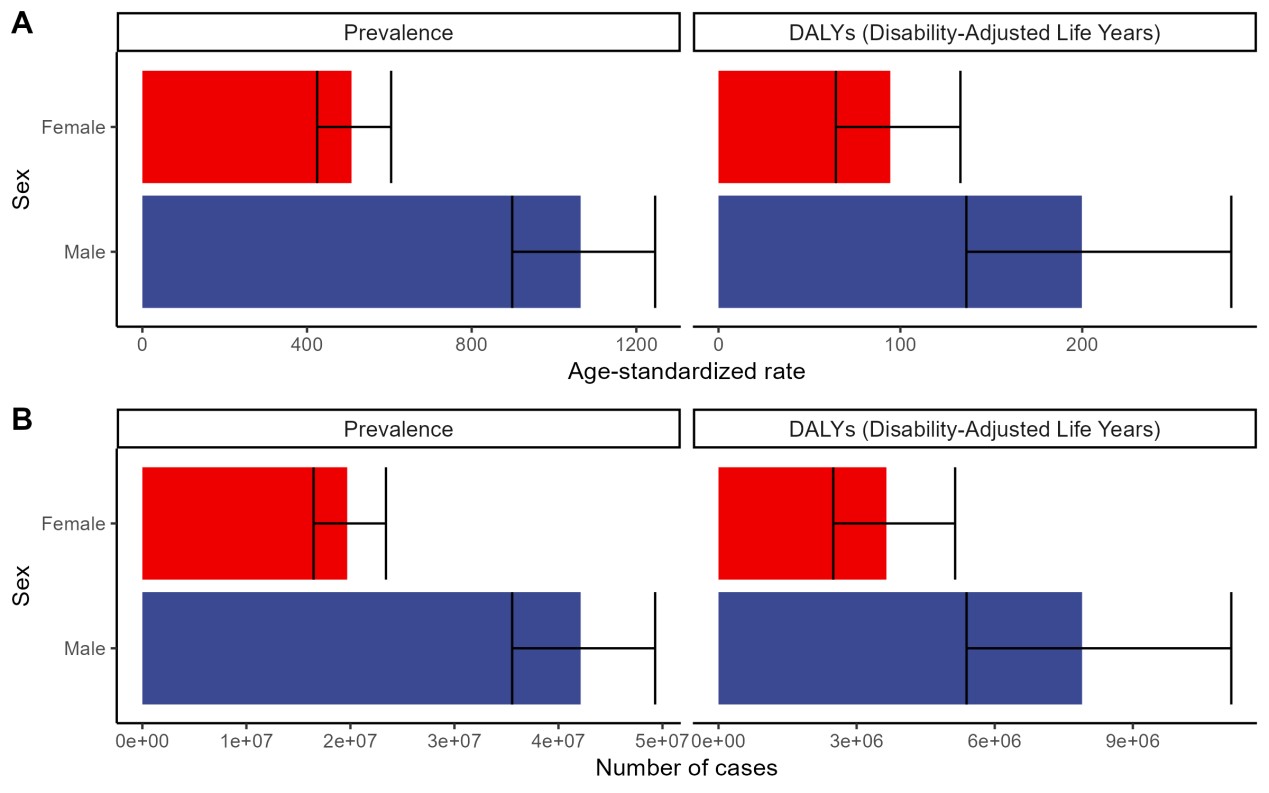


## **Figure S2.** Numbers and age-standardized rates of autism spectrum disorders-related prevalence and DALYs for different age groups in 2021. Abbreviations: DALYs, disability-adjusted life years.


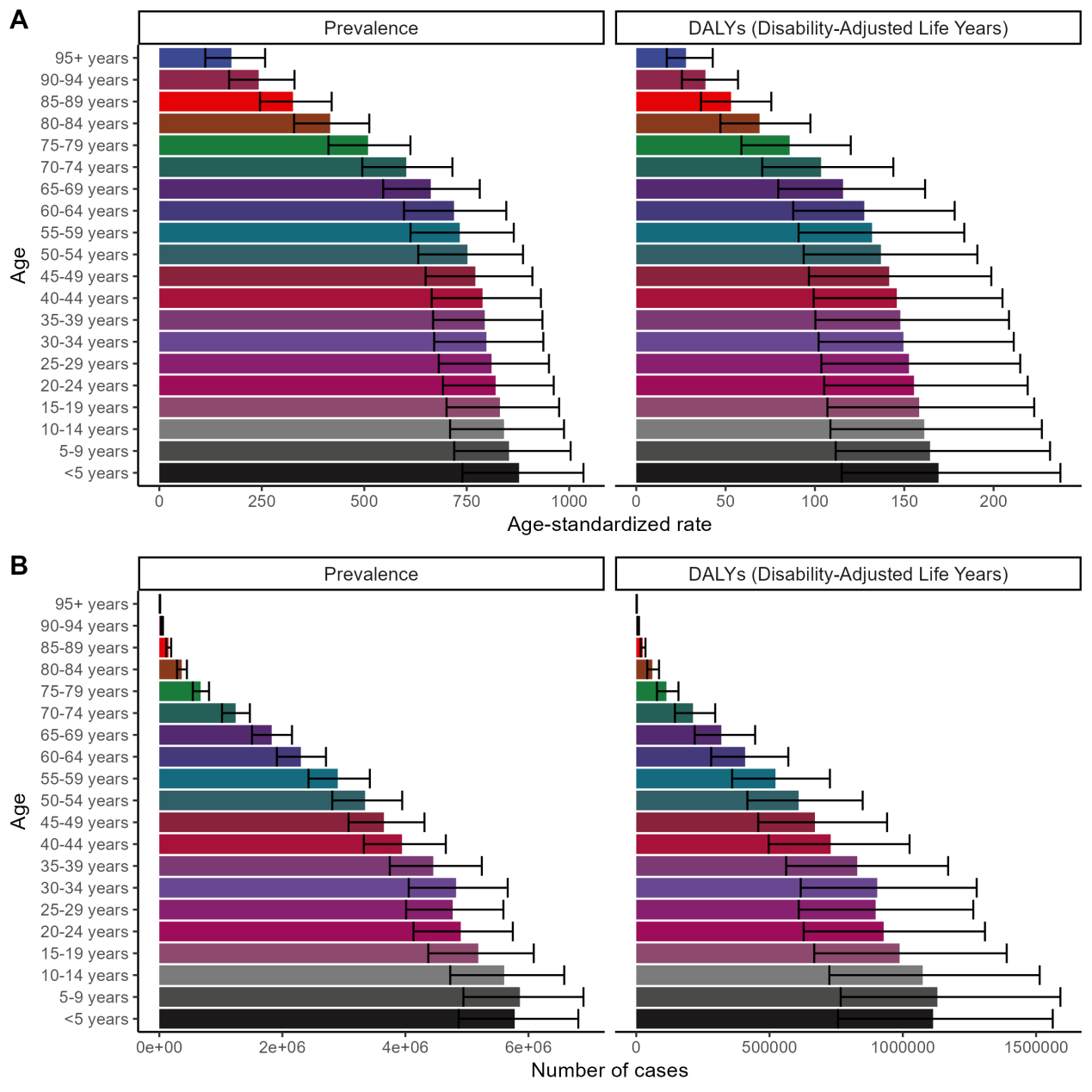


## **Figure S3.** Numbers and age-standardized rates of autism spectrum disorders-related prevalence and DALYs for different SDI regions in 2021. Abbreviations: DALYs, disability-adjusted life years.


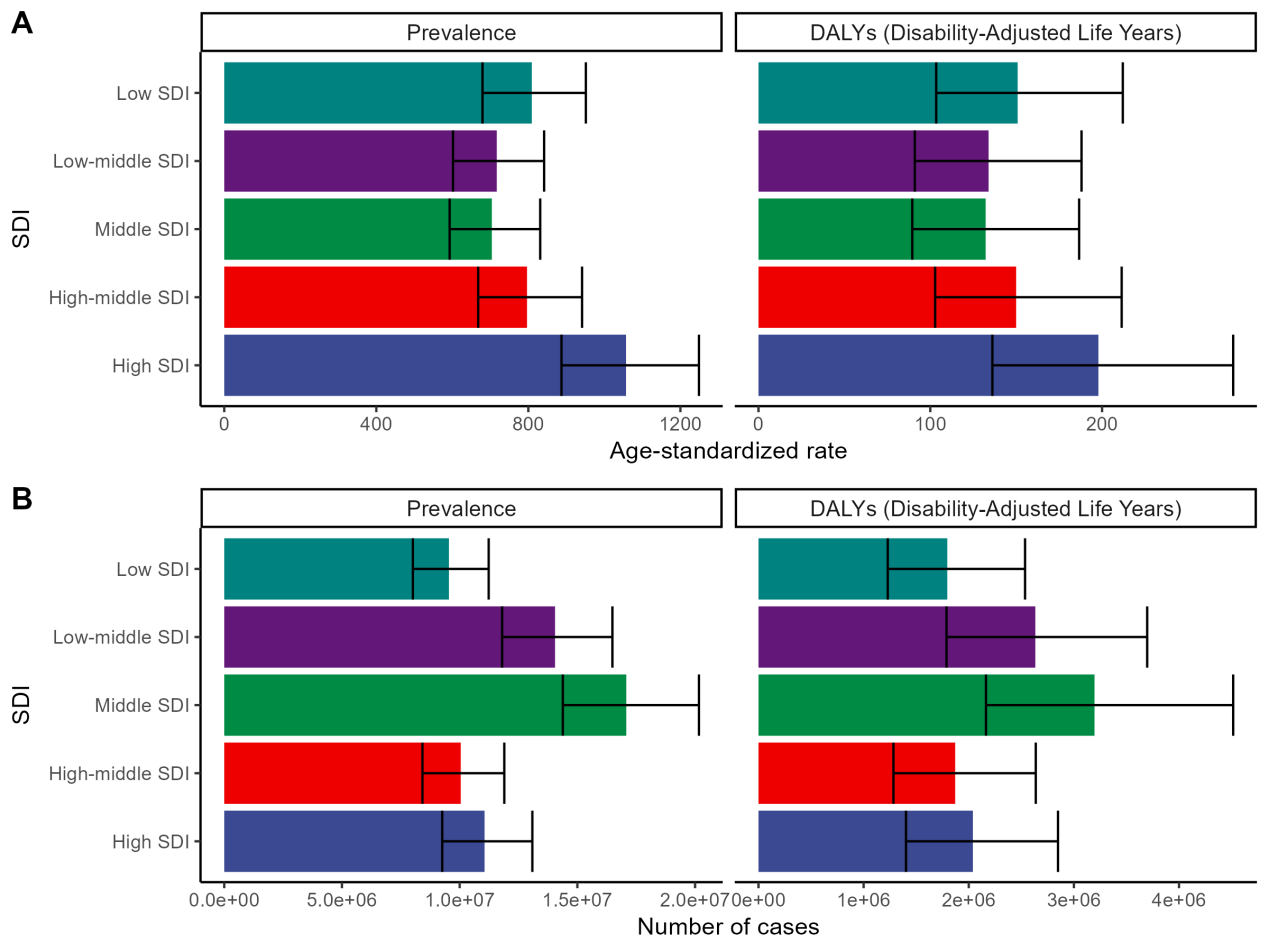


## **Figure S4.** Age-standardized rates of prevalence and DALYs attributable to autism spectrum disorders across countries and territories by socio-demographic index for both sexes, 1990-2021. The black line was an adaptive association fitted with adaptive Loess regression based on all data points. Abbreviations: DALYs, disability-adjusted-life-years.

**
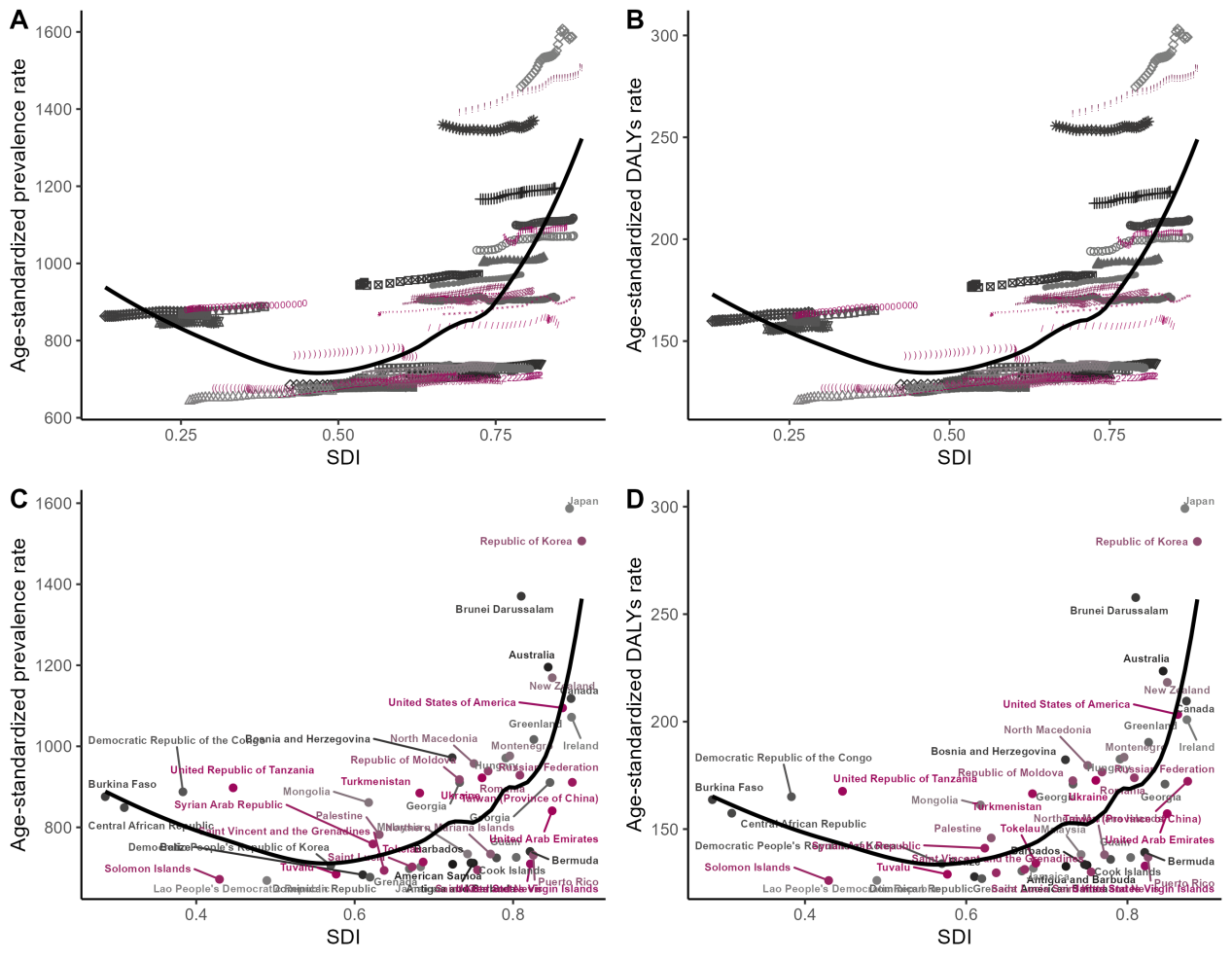
**

## **Figure S5.** Numbers and age-standardized rates of autism spectrum disorders-related prevalence and DALYs for different GBD regions in 2021. Abbreviations: DALYs, disability-adjusted life years.


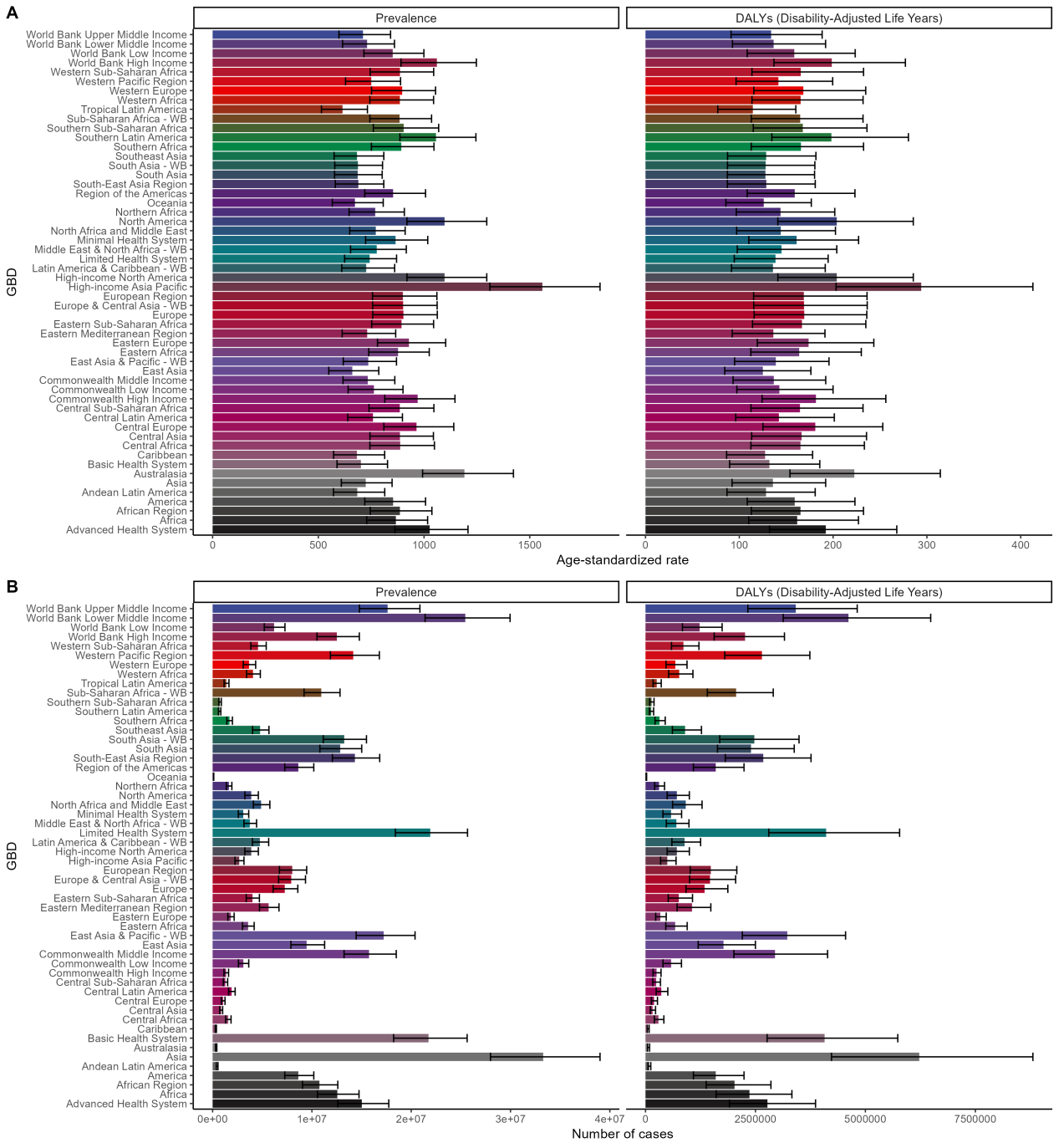


## **Figure S6.** Trends in the numbers and age-standardized rates of autism spectrum disorders-related prevalence and DALYs globally from 1990 to 2021. Abbreviations: DALYs, disability-adjusted-life-years.


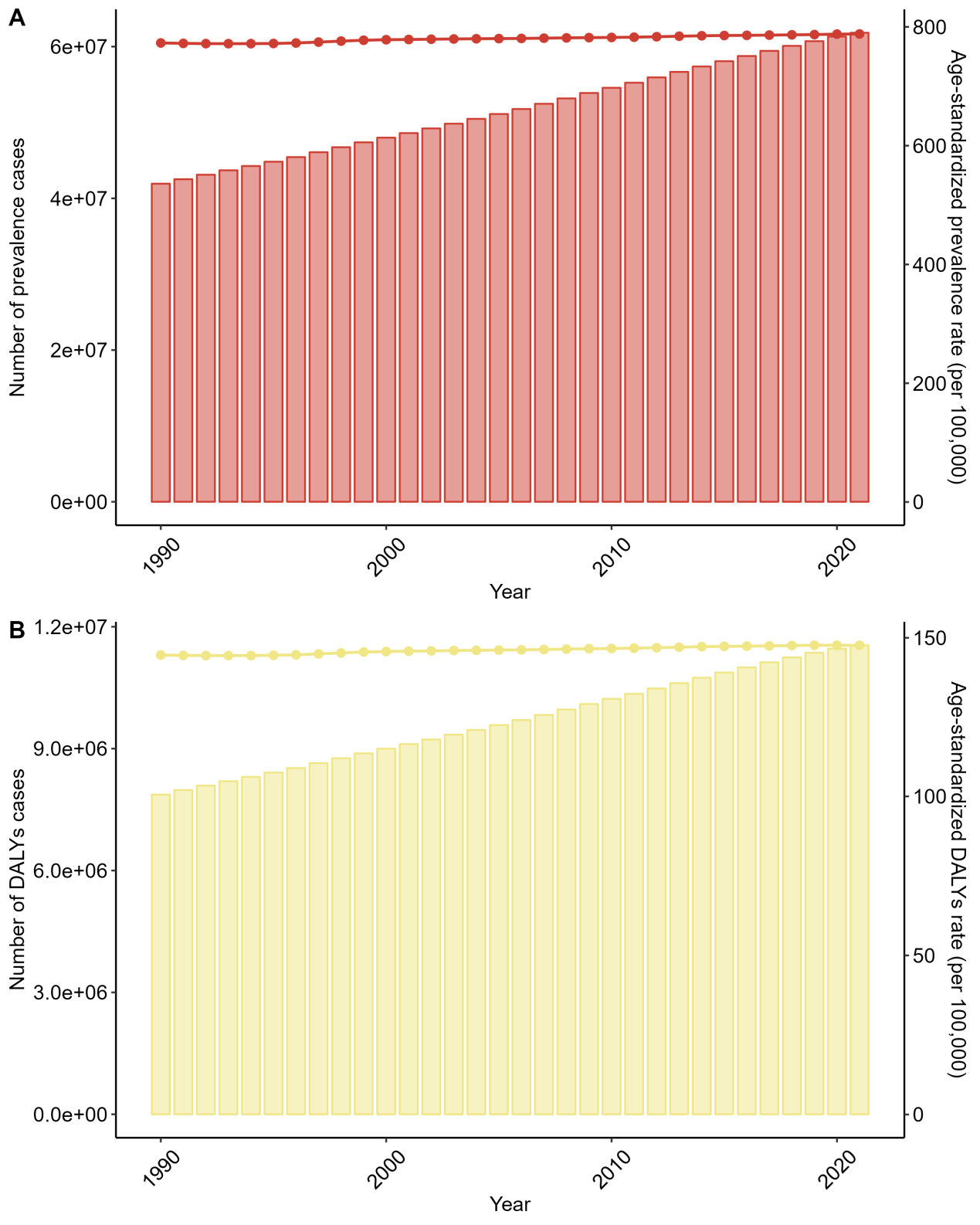


## **Figure S7.** Trends in the numbers and age-standardized rates of autism spectrum disorders-related prevalence and DALYs globally by sexes from 1990 to 2021. Abbreviations: DALYs, disability-adjusted-life-years.


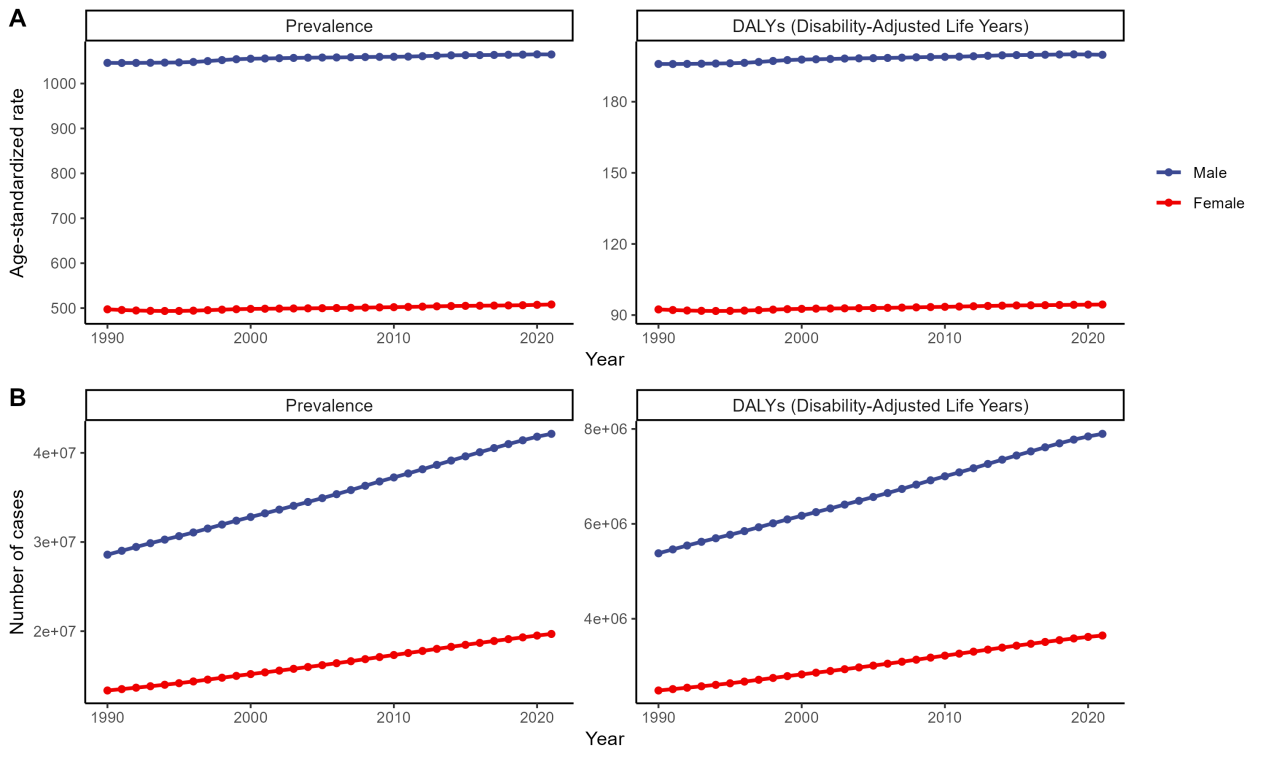


## **Figure S8.** Trends in the numbers and age-standardized rates of autism spectrum disorders-related prevalence and DALYs globally by age groups from 1990 to 2021. Abbreviations: DALYs, disability-adjusted-life-years.


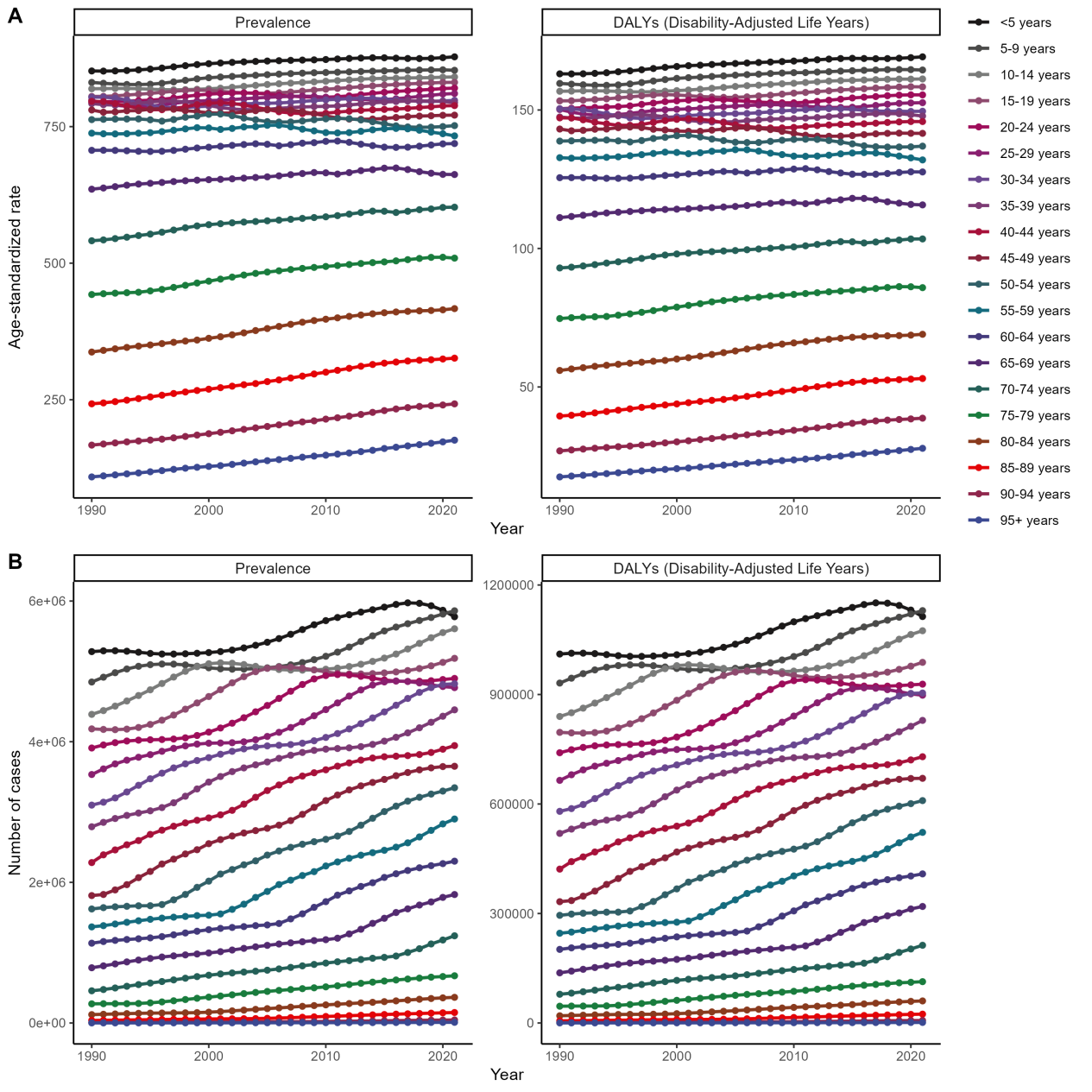


## **Figure S9.** Trends in the numbers and age-standardized rates of autism spectrum disorders-related prevalence and DALYs globally by SDI regions from 1990 to 2021. Abbreviations: DALYs, disability-adjusted-life-years.


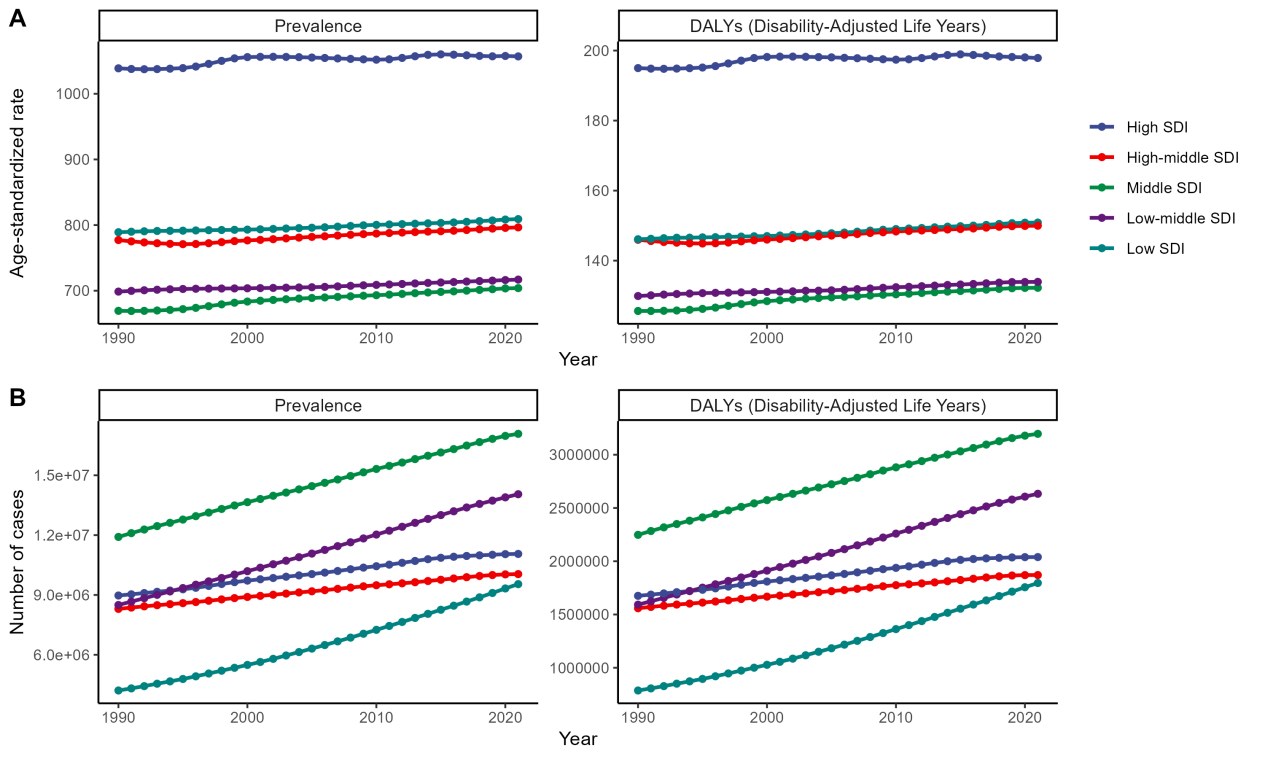


## **Figure S10.** Results of cluster analysis based on the EAPC values of the autism spectrum disorders-related age-standardized rates for prevalence and DALYs from 1990 to 2021. Abbreviations: EAPC, estimated annual percentage change; DALYs, disability-adjusted-life-years.


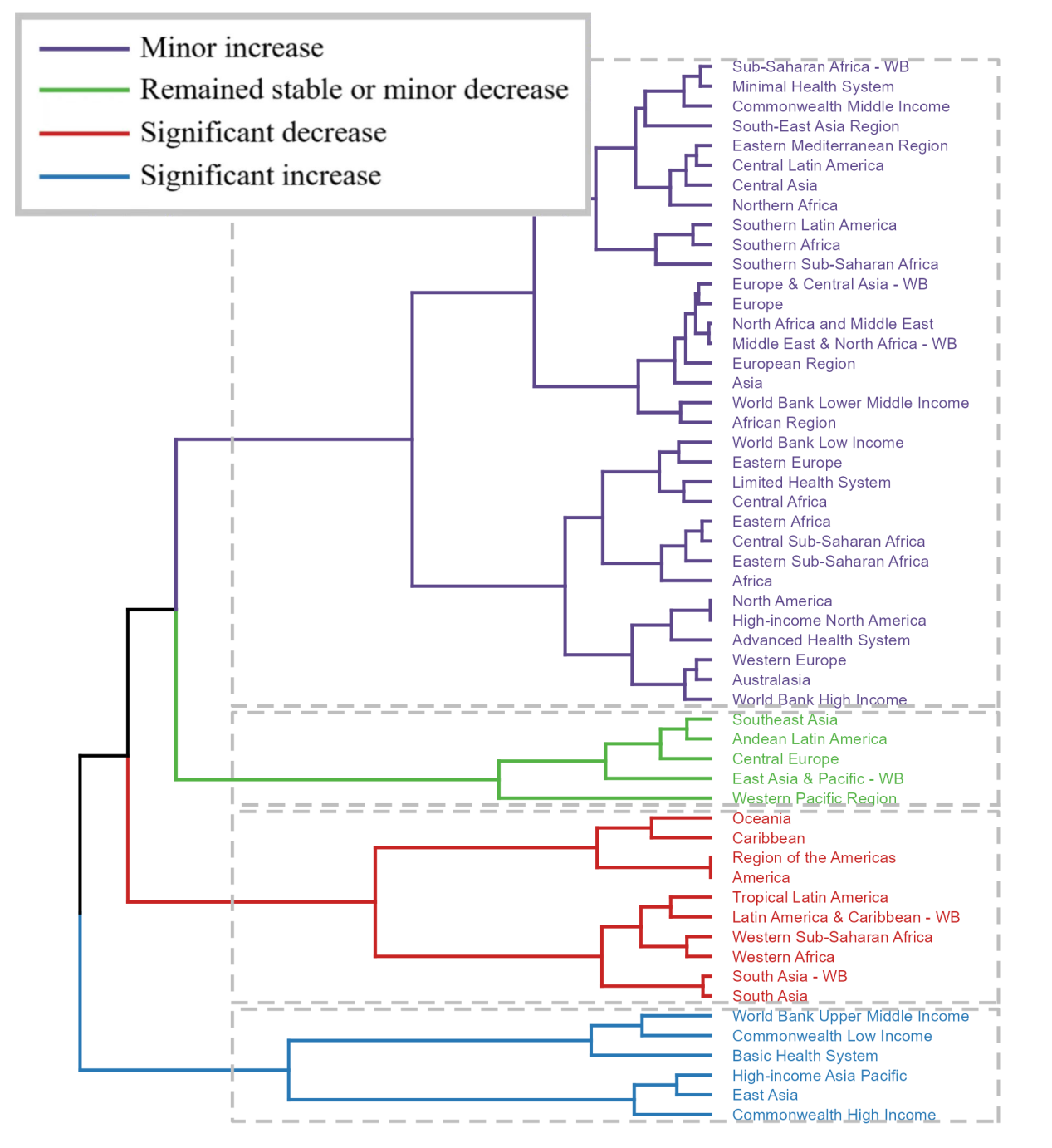


## **Figure S11.** The association between EAPCs and autism spectrum disorders-related ASRs in 1990 and HDIs in 2021. The circles represent countries that were available on HDI data. The size of the circle is increased with the cases of autism spectrum disorders. The ρ indices and p values presented were derived from Spearman correlation analysis. Abbreviations: EAPC, estimated annual percentage change; ASR, age-standardized rate; HDI, human development index.


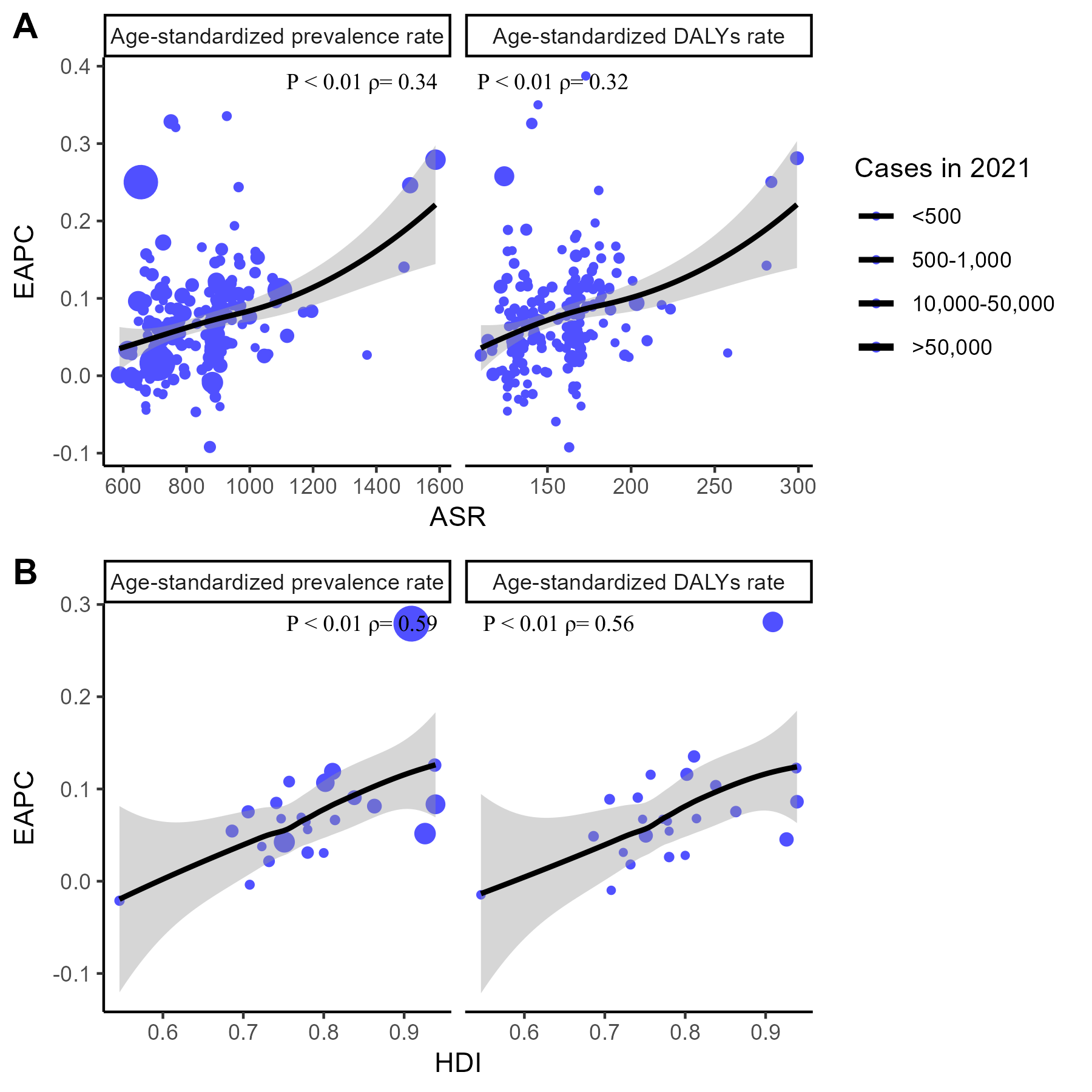


## **Figure S12.** Frontier analysis based on SDI and ASRs of the autism spectrum disorders in 2021. The frontier is delineated in solid black color; countries and territories are represented as dots. The top 15 countries with the largest effective difference (largest ASRs gap from the frontier) are labeled in black; examples of frontier countries with low SDI (< 0.5) and low effective difference are labeled in blue, and examples of countries and territories with high SDI (> 0.85) and relatively high effective difference for their level of development are labeled in red. Red dots indicate an increase in age-standardized ASRs from 1990 to 2021; blue dots indicate a decrease in ASRs between 1990 and 2021. Abbreviations: ASRs: Age-Standardized Rates; SDI: Socio-demographic index.


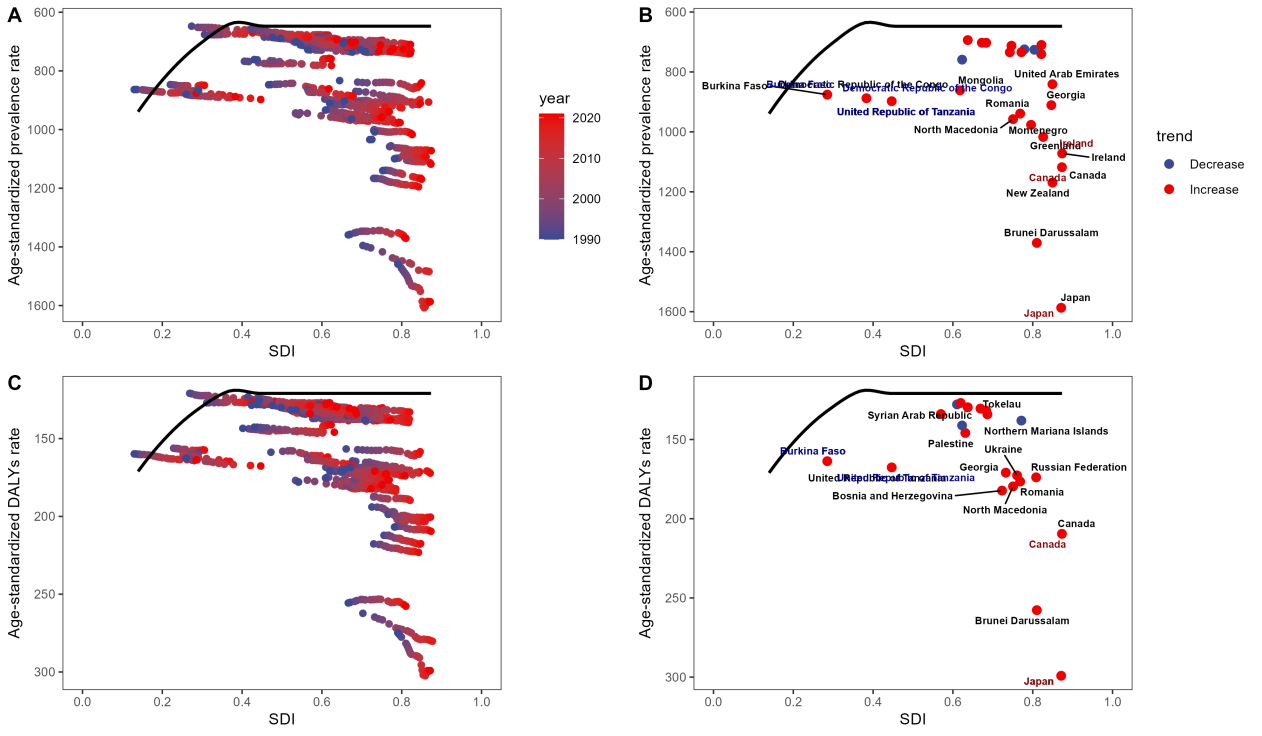

Supplement: Supplementary file 1 [file DataSheet1.docx]
